# Deep sequencing, profiling and detailed annotation of microRNAs in *Takifugu rubripes*

**DOI:** 10.1186/s12864-015-1622-1

**Published:** 2015-06-16

**Authors:** Chaninya Wongwarangkana, Kazuhiro E. Fujimori, Masaki Akiba, Shigeharu Kinoshita, Morimi Teruya, Maiko Nezuo, Tsukahara Masatoshi, Shugo Watabe, Shuichi Asakawa

**Affiliations:** Department of Aquatic Bioscience, Graduate School of Agricultural and Life Sciences, The University of Tokyo, Bunkyo, 113-8657 Tokyo Japan; Bio-production Research Institute, National Institute of Advanced Industrial Science and Technology (AIST), Central 6, 1-1-1 Higashi, Tsukuba, 305-8566 Japan; Okinawa Cutting-edge Genome Project, Okinawa, Japan; Okinawa Industrial Technology Center, Okinawa, 904-2234 Japan; Biojet Co., Ltd, 315 Shioya, Uruma, 904-2231 Okinawa, Japan; School of Marine Bioscience, Kitasato University, Minami, Sagamihara, 252-0373 Kanagawa Japan

**Keywords:** Small RNA, miRNA, piRNA, *T. rubripes*, Next-generation sequencing, miRNA expression profile, Transcriptome

## Abstract

**Background:**

microRNAs (miRNAs) in fish have not been as extensively studied as those in mammals. The fish species *Takifugu rubripes* is an intensively studied model organism whose genome has been sequenced. The *T. rubripes* genome is approximately eight times smaller than the human genome, but has a similar repertoire of protein-coding genes. Therefore, it is useful for identifying non-coding genes, including miRNA genes. To identify miRNA expression patterns in different organs of *T. rubripes* and give fundamental information to aid understanding of miRNA populations in this species, we extracted small RNAs from tissues and performed deep sequencing analysis to profile *T. rubripes* miRNAs. These data will be of assistance in functional studies of miRNAs in *T. rubripes*.

**Results:**

After analyzing a total of 139 million reads, we found miRNA species in nine tissues (fast and slow muscles, heart, eye, brain, intestine, liver, ovaries, and testes). We identified 1420 known miRNAs, many of which were strongly expressed in certain tissues with expression patterns similar to those described for other animals in previous reports. Most miRNAs were expressed in tissues other than the ovaries or testes. However, some miRNA families were highly abundant in the gonads, but expressed only at low levels in somatic tissue, suggesting specific function in germ cells. The most abundant isomiRs (miRNA variants) of many miRNAs had identical sequences in the 5′ region. However, isomiRs of some miRNAs, including fru-miR-462-5p, varied in the 5′ region in some tissues, suggesting that they may target different mRNA transcripts. Longer small RNAs (26–31 nt), which were abundant in the gonads, may be putative piRNAs because of their length and their origin from repetitive elements. Additionally, our data include possible novel classes of small RNAs.

**Conclusions:**

We elucidated miRNA expression patterns in various organs of *T. rubripes*. Most miRNA sequences are conserved in vertebrates, indicating that the basic functions of vertebrate miRNAs share a common evolution. Some miRNA species exhibit different distributions of isomiRs between tissues, suggesting that they have a broad range of functions.

**Electronic supplementary material:**

The online version of this article (doi:10.1186/s12864-015-1622-1) contains supplementary material, which is available to authorized users.

## Background

MicroRNAs (miRNAs) are a dominant class of short transcripts that regulate gene expression in both animals and plants [1]. Most miRNA genes are transcribed by RNA polymerase II, producing a long hairpin structure called a primary miRNA (pri-miRNA) [2]. The pri-miRNA is subsequently processed to a precursor miRNA (pre-miRNA) by a microprocessor called Drosha [3–5]. Exportin-5 then mediates the transport of the pre-miRNA to the cytoplasm where it is further processed to a ~22 nt miRNA duplex by Dicer [6]. Usually, one strand of the duplex is loaded onto an AGO protein [7, 8] and this complex then binds to the 3′-untranslated region of mRNAs and functions in transcript degradation or translational repression [1, 9]. In recent years, many studies of miRNAs in animals have been published. In miRBase 19.0, there are 2042 human, 1281 mouse (*Mus musculus*), 791 chicken (*Gallus gallus*), and 247 zebrafish (*Danio rerio*) mature miRNAs recorded [10]. Although the functions of most miRNAs have not yet been fully elucidated, the majority of miRNA sequences are evolutionarily conserved among species, indicating that they have a conserved biological function [11]. Studies of model organisms, such as small fish, provide convenient ways to understand how miRNAs function, which is often difficult in humans [12].

The zebrafish, whose draft genome was reported recently, is the most popular species in which to analyze fish miRNAs [12]. Most studies of miRNAs in zebrafish have explored their roles in tissue development and differentiation [13, 14]; there has been less focus on expression in specific tissues. Therefore, tissue-specific miRNAs remain to be identified in fish. In addition, although there are 344 miRNA genes encoded in the zebrafish genome, as reported in miRBase 19.0 [10], this number is far lower than that in humans (1600) and mice (855), suggesting that miRNAs in fish have not been fully catalogued. The investigation of miRNAs in *T. rubripes*, in addition to that in zebrafish, would be a significant contribution to our knowledge of fish miRNAs and would provide essential information concerning the biological function of many conserved miRNAs in vertebrates. However, even though most miRNA sequences are conserved in vertebrates, some miRNAs with low expression levels seem to be non-conserved or organism specific. The identification and characterization of fish-specific miRNAs would contribute to our understanding of how fish are different from other vertebrates.

In this study, we performed next-generation sequencing of small RNAs in *T. rubripes* to identify miRNA expression patterns in different organs of *T. rubripes* and give fundamental information to aid understanding of miRNA populations in this species. These data will be of assistance in functional studies of miRNAs in *T. rubripes*.

## Results

### Sequencing of *T. rubripes* small RNAs

We obtained a total of 138,786,706 reads of 35 nucleotides, with additional exact barcode sequences. Low-quality reads were eliminated using our own Perl scripts. After the trimming of 3′ adaptors, 50,653,035 small RNA reads of 1–35 nucleotides were retrieved (Additional files 1 and 2).

### Size distribution of small RNAs

**Fig. 1:**
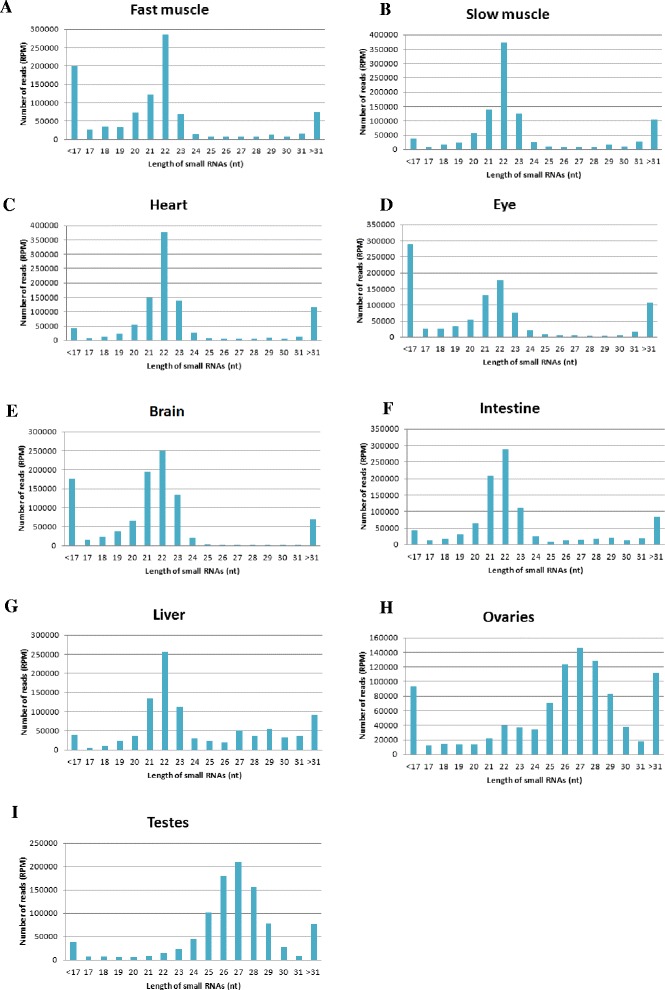
Size distribution pattern of small RNAs in different tissues of *T. rubripes*. Size distribution pattern of small RNA reads after normalizing against 1 million 1–35 nt small RNA reads; fast muscle (**a**), slow muscle (**b**), heart (**c**), eye (**d**), brain (**e**), intestine (**f**), liver (**g**), ovaries (**h**), and testes (**i**)

We constructed a small RNA size distribution profile for each tissue. In somatic tissue, small RNAs of 22 nucleotides were the most abundant, followed by those of 21 and 23 nucleotides. In the ovaries and testes, the vast majority of small RNAs were 26–28 nucleotides in length, with most having 27 nucleotides (Fig. 1).

### Proportion of miRNAs in individual tissues

**Fig. 2:**
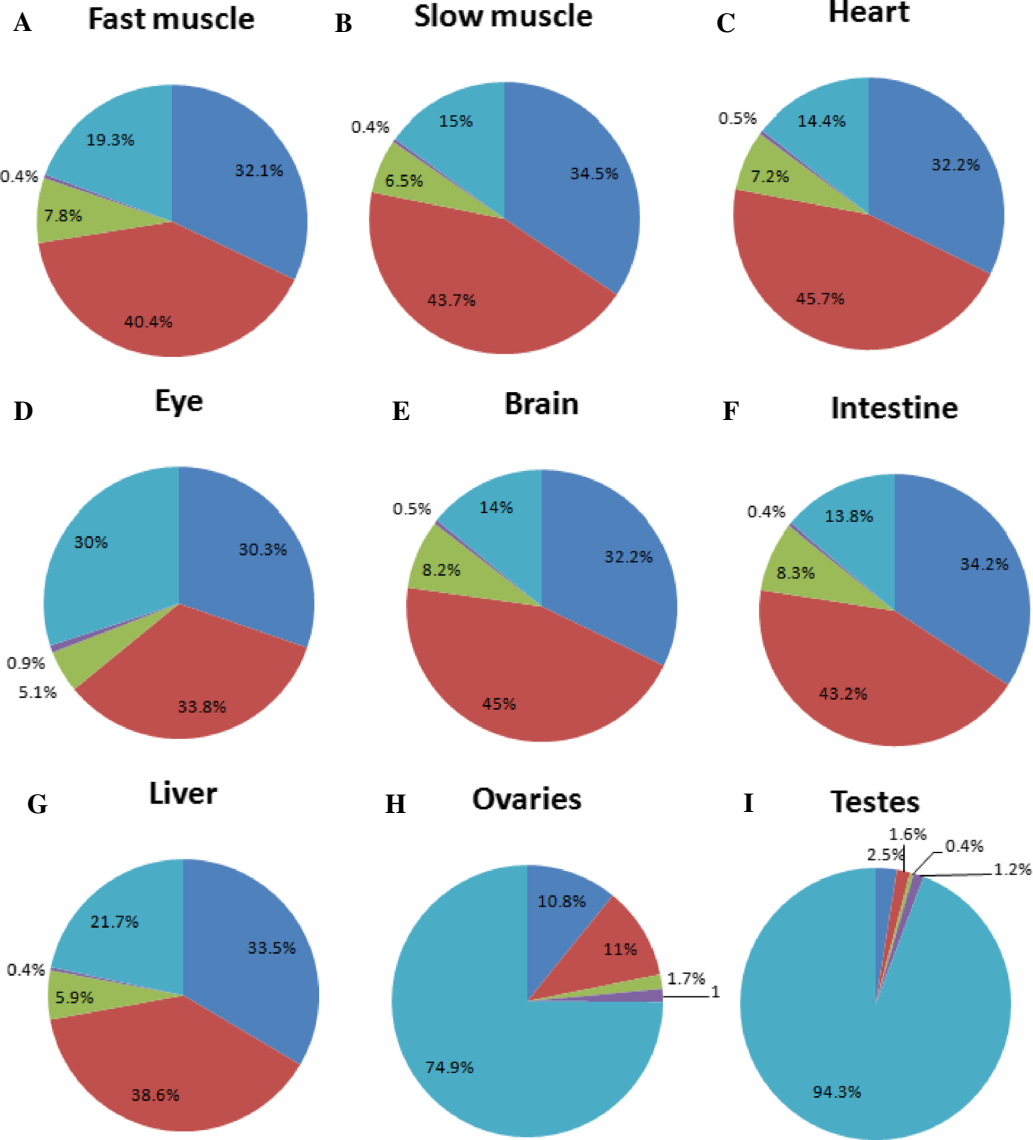
miRNA proportions as percentages of 18–25 nt small RNAs. The dark blue color indicates reads that exactly matched the reference sequences, 1–2 nt extended or shortened at the 5′ end, or 1–4 nt extended or shortened at the 3′ end of known miRNAs. The red color indicates the complete match of continuous sequences of 16 nt or more. The green color indicates the matching of continuous sequences of less than 16 nt with a Smith-Waterman score equal to or more than 70. The purple color indicates the matching of continuous sequences of less than 16 nt with a Smith-Waterman score less than 70. The light blue color indicates 18–25 nt unidentified small RNAs. Fast muscle (**a**), slow muscle (**b**), heart (**c**), eye (**d**), brain (**e**), intestine (**f**), liver (**g**), ovaries (**h**), and testes (**i**)

Because miRNA sequences are conserved in vertebrates, we identified miRNAs by conducting a homology search against the miRNA database. After investigating the length distributions of known miRNAs deposited in miRBase 19.0 [10], we found that almost every miRNA was 18–25 nt in length. Therefore, small RNA sequences (18–25 nucleotides in length) were subjected to a BLAST search against mature miRNAs deposited in miRBase 19.0 [10]. The criteria for miRNA annotation were as follows (Additional file 1): 1. Query reads exactly match the reference sequences; 2. Reads with 1–2 nt extended or shortened at the 5′ end, and/or reads with 1–4 nt extended or shortened at the 3′ end of known miRNAs. Using these criteria, among the small RNA sequence reads approximately 32.1 % in fast muscle, 34.5 % in slow muscle, 32.2 % in heart, 30.3 % in eye, 32.2 % in brain, 34.2 % in intestine, and 33.5 % in liver were identified as miRNAs. However, the proportion of small RNA sequence reads that were miRNAs in the ovaries and testes was only 10.8 and 2.5 %, respectively (Fig. 2, Additional file 3).

Because of these stringent criteria, large numbers small RNAs were not identified, because of possible 3′ variations or sequence errors. We then employed more flexible criteria (Additional file 1). Specifically, if sequences of the first 10 nucleotides were identical to those of known miRNAs, we performed further analysis. Among these sequences, we first investigated those that completely matched a continuous miRNA sequence of 16 nt or more. Following these criteria, among the small RNA sequence reads, approximately 40.4 % in fast muscle, 43.7 % in slow muscle, 45.7 % in heart, 33.8 % in eye, 45 % in brain, 43.2 % in intestine, 38.6 % in liver, 11 % in ovaries, and 1.6 % in testes were identified as miRNAs. If the continuous sequences of perfect match were less than 16 nt, we used a Smith-Waterman algorithm in FASTA v36 to evaluate the reads. We set the parameters as ‘5’ for match and ‘−4’ for mismatch. According to this algorithm, we considered the sequences with Smith-Waterman scores of at least 70 as miRNAs. According to these criteria, among the small RNA sequences, 7.8 % in fast muscle, 6.5 % in slow muscle, 7.2 % in heart, 5.1 % in eye, 8.2 % in brain, 8.3 % in intestine, 5.9 % in liver, 1.7 % in ovaries, and 0.4 % in testes, were retrieved as miRNAs. Among the reads in which the first 10 nucleotides were identical with those of known miRNAs, the remaining sequences were annotated as putative miRNAs, which represented 0.4 % in fast muscle, 0.4 % in slow muscle, 0.5 % in heart, 0.9 % in eye, 0.5 % in brain, 0.4 % in intestine, 0.4 % in liver, 1.5 % in ovaries, and 1.2 % in testes (Fig. 2, Additional files 4 and 5).

The vast majority of miRNAs in all tissues was approximately 21–23 nt in length. In the eye, brain, and intestine, the numbers of miRNAs that were 21 nt in length were similar to the numbers that were 22 nt in length (Additional file 6). For putative miRNAs, sequences that were 22 nt were the most abundant in skeletal muscle, heart, and liver. Putative miRNAs that were 18 nt in size were most abundant in the brain and intestine. In the eye, putative miRNAs that were 20 nt in length were the most highly expressed (Additional file 6).

We also investigated the proportion of miRNAs in small RNAs of 18–25 nt in each tissue. Approximately 50–90 % of small RNAs of the nt length 20–24 in fast muscle, 19–24 in slow muscle, 19–25 in heart, 21–23 in eye, 18–25 in brain, 19–24 in intestine, and 19–23 in liver were categorized as miRNAs, including putative miRNAs. In the gonads, for each length of small RNA between 18 and 25 nt, less than 50 % were identified as miRNAs, including putative miRNAs, except for small RNAs of 22 nt in the ovaries (Additional file 7).

We constructed a pie chart of the proportion of miRNAs in small RNA populations of 1–35 nt. More than 50 % of small RNAs in skeletal muscle, heart, brain, and intestine were identified as miRNAs, and in the eye and liver, 36.6 and 49.1 %, respectively, were categorized as miRNAs. In the ovaries and testes, only 5.8 and 1 %, respectively, of the small RNAs were annotated as miRNAs. In addition, the major proportion of small RNAs in the gonads was 26–31 nt (Additional file 8).

### Expression profile of *T. rubripes* miRNAs in different tissues

**Fig. 3:**
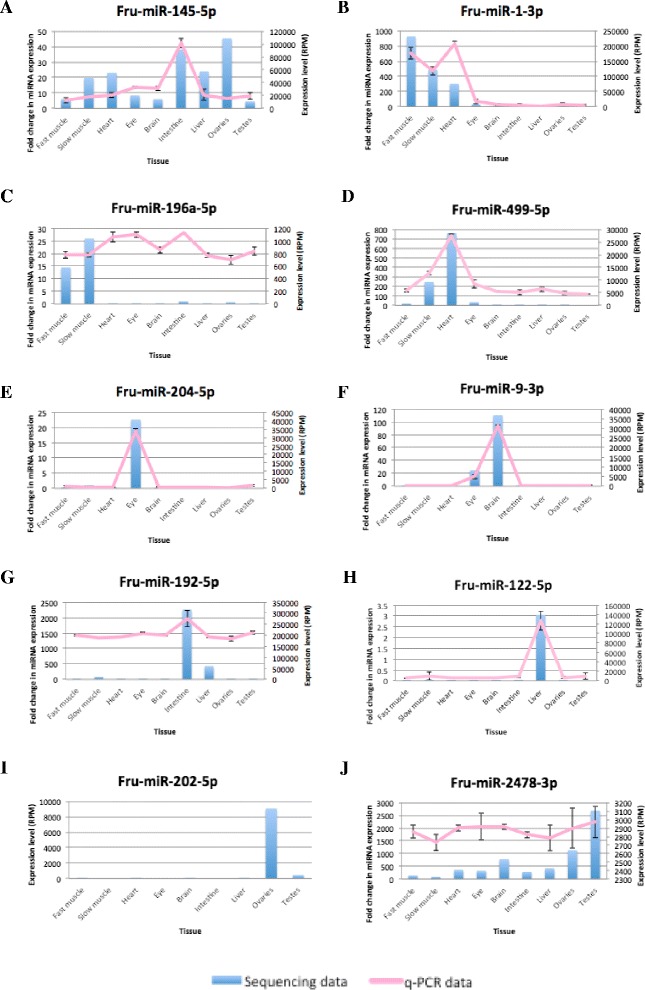
Comparison of next-generation sequencing and qPCR-based profiling of representative ubiquitous and tissue-specific miRNAs. Expression profiles of ubiquitous (**a**), muscle-specific (**b**), skeletal muscle-specific (**c**), heart and slow muscle-specific (**d**), eye-specific (**e**), brain and eye-specific (**f**), intestine-specific and liver-specific (**g**), liver-specific (**h**), ovary-specific (**i**), and testes-abundant (**j**) miRNAs. The left-hand y axis shows the result from q-PCR and right-hand y axis shows the result from next-generation sequencing

For each tissue, we counted the number of each miRNA and generated a miRNA expression profile across the tissues. The number of reads of individual miRNAs was normalized against the total number of 18–25 nt small RNA reads in each tissue, giving the number of reads for an individual as miRNAs per million (Additional file 9). The expression profile showed that many miRNAs were highly expressed in more than one tissue, or were ubiquitously expressed, such as fru-miR-145-5p, which exhibited a high expression level in all tissues. However, some miRNAs exhibited a biased expression in specific tissues. For example, fru-miR-1-3p was muscle specific, fru-miR-196a-5p was skeletal muscle specific, fru-miR-499-5p was heart and slow muscle specific, fru-miR-204-5p was eye specific, fru-miR-9-3p was brain and eye specific, fru-miR-192-5p was intestine and liver specific, fru-miR-122-5p was liver specific, and fru-miR-202-5p was ovary specific. Even though the ovaries and testes had a much lower proportion of miRNAs than somatic tissue, some miRNAs, such as fru-miR-202-5p and fru-miR-2478-3p, had higher levels of expression in the ovaries and testes, respectively, than in somatic cells. These results were confirmed by stem-loop reverse-transcription polymerase chain reaction (RT-PCR) (Fig. 3).

### Quantitative PCR of representative ubiquitous and tissue-specific miRNAs in *T. rubripes*

Ten miRNAs, identified above, were selected for stem-loop quantitative PCR analysis to confirm the sequencing results (Fig. 3). The next-generation sequencing results of fru-miR-145-5p, fru-miR-1-3p, fru-miR-204-5p, fru-miR-9-3p and fru-miR-122-5p correlated well with those of q-PCR. However, for fru-miR-499-5p, fru-miR-192-5p, fru-miR-196a-5p, fru-miR-202-5p (data not shown) and fru-miR-2478-3p, the results from q-PCR were not consistent with the sequencing results. These inconsistencies may be caused by the primers that were not specific enough to the target miRNAs.

### Putative differentially expressed miRNAs in muscle tissue

To identify miRNAs that exhibited specific expression in fast, slow, or cardiac muscles, the relative ratios of normalized reads were used to identify differentially expressed miRNAs among the muscle tissues. First, we compared the miRNA expression profile between fast and cardiac muscle. Fru-miR-206-3p, fru-miR-10b-5p, fru-miR-10d-5p, fru-miR-133b-3p, and fru-miR-133-3p exhibited 434, 77, 60, 17, and 15 times higher levels of expression, respectively, in fast muscle compared with cardiac muscle, while fru-miR-144-5p, fru-miR-499-5p, fru-miR-187-3p, fru-miR-499a-5p, and fru-miR-140-3p exhibited 51-, 41-, 37-, 33-, and 17-fold higher expression levels, respectively, in heart muscle compared with fast muscle. Second, we compared miRNA expression profiles between slow and cardiac muscle. Expression levels of fru-miR-196a-5p, fru-miR-206-3p, fru-miR-194-5p, fru-miR-192-5p, and fru-miR-10b-5p in slow muscle were 1305-, 529-, 93-, 85-, and 77-fold higher, respectively, compared with cardiac muscle, while expression levels of fru-miR-187-3p, fru-miR-30e-3p, fru-miR-140-3p, fru-miR-218a-5p, and fru-miR-140-5p were 16-, 16-, 10-, 8-, and 6-fold higher, respectively, in cardiac muscle compared with slow muscle. We also compared miRNA expression levels between the skeletal muscles. Fru-miR-30e-3p exhibited an approximately 4-fold higher expression level in fast muscle compared with slow muscle. Fru-miR-133a-3p, fru-miR-133a-5p, and fru-miR-3571-5p exhibited an approximately 3-fold higher level of expression, in fast muscle compared with slow muscle. In contrast, fru-miR-126b-5p, fru-miR-194-5p, fru-miR-499-5p, fru-miR-30e-5p, and fru-miR-30c-5p exhibited 121-, 21-, 13-, 11-, and 4-fold higher levels of expression, respectively, in slow muscle compared with fast muscle (Table 1). However, these differential results are putative because they are base on one transcriptome analysis.

**Table 1:** Top five putative differentially expressed miRNAs compared between 2 muscle tissues

Fast muscle > cardiac muscle			
miRNA	Fast muscle (RPM)	Fast muscle/heart	Heart (RPM)
fru-miR-206-3p	94,778	434	218
fru-miR-10b-5p	2310	77	30
fru-miR-10d-5p	1516	60	25
fru-miR-133b-3p	16,735	17	1003
fru-miR-133-3p	58,952	15	3951
Cardiac muscle > fast muscle			
miRNA	Heart (RPM)	Heart/fast muscle	Fast muscle (RPM)
fru-miR-144-5p	1012	51	20
fru-miR-499-5p	28,506	41	699
fru-miR-187-3p	1483	37	40
fru-miR-499a-5p	1179	33	36
fru-miR-140-3p	10,721	17	613
Slow muscle > cardiac muscle			
miRNA	Slow muscle (RPM)	Slow muscle/heart	Heart (RPM)
fru-miR-196a-5p	1035	1305	1
fru-miR-206-3p	115,467	529	218
fru-miR-194-5p	1065	93	12
fru-miR-192-5p	11 732	85	138
fru-miR-10b-5p	2329	77	30
Cardiac muscle > slow muscle			
miRNA	Heart (RPM)	Heart/slow muscle	Slow muscle (RPM)
fru-miR-187-3p	1483	16	92
fru-miR-30e-3p	4473	16	280
fru-miR-140-3p	10,721	10	1108
fru-miR-218a-5p	1380	8	168
fru-miR-140-5p	4288	6	673
Fast muscle > slow muscle			
miRNA	Fast muscle (RPM)	Fast muscle/slow muscle	Slow muscle (RPM)
fru-miR-30e-3p	1239	4	280
fru-miR-133a-3p	48,921	3	14,148
fru-miR-133a-5p	14,675	3	5286
fru-miR-3571-5p	3257	3	1176
fru-miR-133-5p	2581	2	1089
Slow muscle > fast muscle			
miRNA	Slow muscle (RPM)	Slow muscle/fast muscle	Fast muscle (RPM)
fru-miR-126b-5p	1350	121	11
fru-miR-194-5p	1065	21	50
fru-miR-499-5p	9193	13	699
fru-miR-30e-5p	6361	11	591
fru-miR-30c-5p	6669	4	1601

miRNAs with more than a 1.5-fold difference in expression between muscle tissues and expression levels of more than 1000 RPM were selected

### Putative differentially expressed miRNAs in the gonads

We used the relative ratios of normalized reads to find differentially expressed miRNAs in the ovaries and testes, to identify sex-specific or sexually dimorphic miRNAs. Of the miRNAs that exhibited higher expression levels in the ovaries compared with the testes, fru-miR-214-3p and fru-miR-143-3p exhibited the greatest differences in expression. They exhibited an approximately 24-fold higher expression level, in the ovaries compared with the testes, followed by fru-miR-202-5p, fru-miR-24-3p, and fru-miR-145b-5p, whose expression levels were approximately 22-, 20-, and 20-fold higher, respectively, in the ovaries compared with the testes. Of the miRNAs that exhibited greater expression in the testes compared with the ovaries, fru-miR-2478-3p and fru-miR-2898-3p exhibited levels in the testes that were twice those in the ovaries (Table 2). However, we could only claim these results as putative differential expression patterns as described above.

**Table 2:** Putative differentially expressed miRNAs compared between ovaries and testes

Ovaries > testes			
miRNA	Ovaries (RPM)	Ovaries/testes	Testes (RPM)
fru-miR-214-3p	3055	24	125
fru-miR-143-3p	6965	24	293
fru-miR-202-5p	9054	22	409
fru-miR-24-3p	4170	20	208
fru-miR-145b-5p	5933	20	302
Testes > ovaries			
miRNA	Testes (RPM)	Testes/ovaries	Ovaries (RPM)
fru-miR-2478-3p	2691	2	1133
fru-miR-2898-3p	1503	2	753

miRNAs with more than a 1.5-fold difference in expression between ovaries and testes and expression levels of more than 1000 RPM were selected

### IsomiRs in *T. rubripes*

IsomiRs refer to mature miRNA sequences in which a few nucleotides vary at the 5′/3′ termini compared with the major mature miRNAs that originated from the same miRNA hairpin. We chose fru-miR-145-5p, which was ubiquitously expressed, as a representative example to demonstrate the variation in mature miRNA sequences. The sequence of fru-miR-145-5p was identical in every tissue. However, the sequence was different at the 3′ terminus compared with the miR-145-5p entries deposited in miRBase 19.0 [10]. Fru-miR-145-5p exhibited more nucleotide variation in the 3′ region than in the 5′ region. Among isomiRs, approximately 47.7 % in fast muscle, 48.2 % in slow muscle, 56.7 % in heart, 58.1 % in eye, 49.2 % in brain, 48.2 % in intestine, 45.1 % in liver, 53.8 % in ovaries, and 44.3 % in testes exhibited 3′ variation compared with the most abundant fru-miR-145-5p. Approximately 0.4 % in fast muscle, 0.4 % in slow muscle, 0.5 % in heart, 0.4 % in eye, 0.4 % in brain, 0.4 % in intestine, 0.6 % in liver, 0.8 % in ovaries, and 1.3 % in testes exhibited variation at both the 3′ and 5′ ends. Less than 0.5 % of isomiRs exhibited only 5′ variation in fast muscle, slow muscle, heart, eye, brain, intestine, liver, ovaries, and testes. A sliding of the Drosha cleavage site at the 5′ terminus occurred in a small population of fru-pri-miR-145 (Additional file 10). This led to the production of different seed sequences in mature fru-miR-145 isomiRs, and they may recognize different target mRNAs. We investigated the major sequence of each miRNA species across the tissues; fru-miR-462-5p, fru-miR-101b-3p, and fru-miR-133-3p exhibited significant variation at the 5′ terminus in some tissues (Table 3, Additional files 11, 12 and 13).

**Table 3:** miRNAs in which major isomiRs exhibited 5′ variation among tissues and their expression levels

miRNA	Major sequence	Seed sequence	Expression level	Tissue
fru-miR-462-5p	GTAACGGAACCCATAATGCAGCT	TAACGGA	47	Fast muscle
fru-miR-462-5p	GTAACGGAACCCATAATGCAGCT	TAACGGA	119	Slow muscle
fru-miR-462-5p	**TAACGGAACCCATAATGCAGCT**	**AACGGAA**	**253**	**Heart**
fru-miR-462-5p	GTAACGGAACCCATAATGCAGCT	TAACGGA	30	Eye
fru-miR-462-5p	**TAACGGAACCCATAATGCAGCT**	**AACGGAA**	**50**	**Brain**
fru-miR-462-5p	GTAACGGAACCCATAATGCAGCT	TAACGGA	1803	Intestine
fru-miR-462-5p	GTAACGGAACCCATAATGCAGCT	TAACGGA	119	Liver
fru-miR-462-5p	GTAACGGAACCCATAATGCAGCT	TAACGGA	87	Ovaries
fru-miR-462-5p	GTAACGGAACCCATAATGCAGCT	TAACGGA	19	Testes
fru-miR-101b-3p	GTACAGTACTATGATAACTGA	TACAGTA	85	Fast muscle
fru-miR-101b-3p	GTACAGTACTATGATAACTGA	TACAGTA	75	Slow muscle
fru-miR-101b-3p	GTACAGTACTATGATAACTGA	TACAGTA	131	Heart
fru-miR-101b-3p	GTACAGTACTATGATAACTGA	TACAGTA	97	Eye
fru-miR-101b-3p	**TACAGTACTATGATAACTGA**	**ACAGTAC**	**106**	**Brain**
fru-miR-101b-3p	GTACAGTACTATGATAACTGA	TACAGTA	72	Intestine
fru-miR-101b-3p	GTACAGTACTATGATAACTGA	TACAGTA	1774	Liver
fru-miR-101b-3p	**TACAGTACTATGATAACTGA**	**ACAGTAC**	**24**	**Ovaries**
fru-miR-101b-3p	GTACAGTACTATGATAACTGA	TACAGTA	15	Testes
fru-miR-133-3p	TTGGTCCCCTTCAACCAGCCGT	TGGTCCC	1958	Fast muscle
fru-miR-133-3p	**TTTGGTCCCCTTCAACCAGCC**	**TTGGTCC**	**785**	**Slow muscle**
fru-miR-133-3p	TTGGTCCCCTTCAACCAGCCGT	TGGTCCC	225	Heart
fru-miR-133-3p	TTGGTCCCCTTCAACCAGCCGT	TGGTCCC	17	Eye
fru-miR-133-3p	TTGGTCCCCTTCAACCAGCCGT	TGGTCCC	4	Brain
fru-miR-133-3p	TTGGTCCCCTTCAACCAGCCGT	TGGTCCC	4	Intestine
fru-miR-133-3p	**TTTGGTCCCCTTCAACCAGCCG**	**TTGGTCC**	**1**	**Liver**
fru-miR-133-3p	TTGGTCCCCTTCAACCAGCCGT	TGGTCCC	6	Ovaries

Bold indicates isomiRs with variation at the 5′ terminus

### Distribution of different small RNA categories in each tissue

**Fig. 4:**
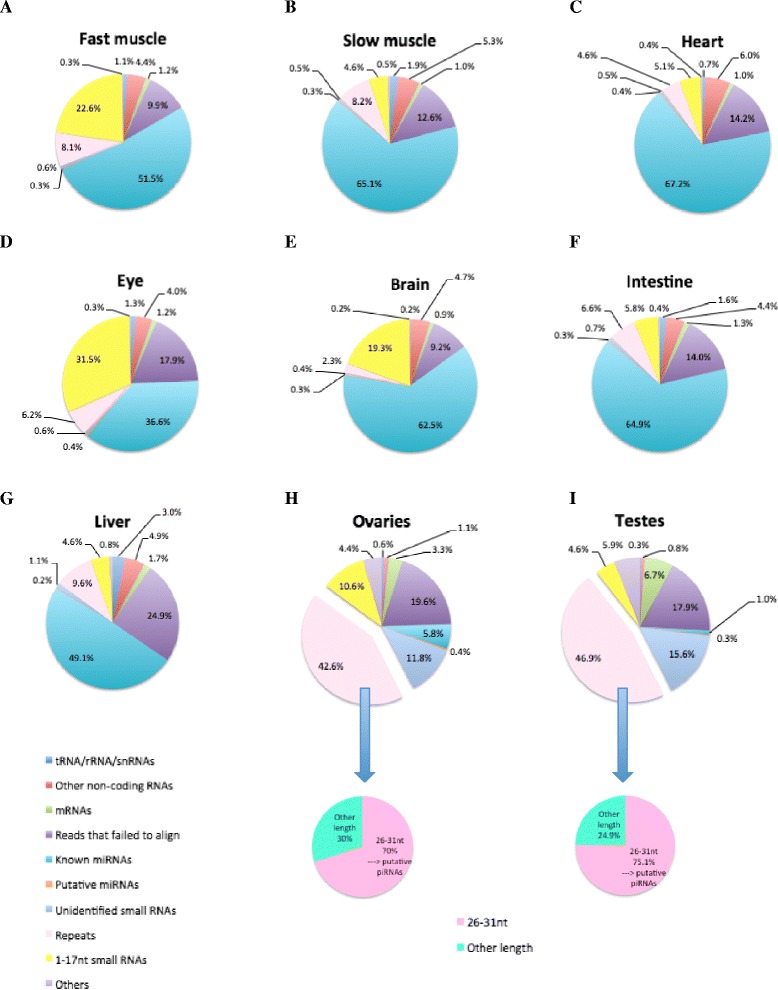
Proportions of small RNA components. Fast muscle (**a**), slow muscle (**b**), heart (**c**), eye (**d**), brain (**e**), intestine (**f**), liver (**g**), ovaries (**h**), and testes (**i**)

To classify the remaining unidentified small RNAs (18–25 nt unidentified small RNAs and 26–35 nt small RNAs), we performed homology searches against the NCBI database (Fig. 4). In the 1–35 nt population, the majority of small RNAs in the ovaries and testes were derived from repetitive elements. Additionally, 70–75 % of them were identified to be 26–31 nt. The major proportion of small RNAs of each length in the ovaries and testes (except for 21–22 nt in the ovaries) were also derived from repetitive elements (Additional file 7). Finally, in each somatic tissue, only about 1 % of small RNAs were unidentified; while in the ovaries and testes 11.8 and 15.6 % were unidentified, respectively.

## Discussion

Most miRNAs are conserved among species; therefore, we identified miRNAs by conducting a homology search against mature miRNAs deposited in miRBase 19.0 [10]. In the 18–25 nt population, 69–86 % of small RNAs in somatic tissue were annotated as miRNAs, whereas only 24 and 5 % of small RNAs in the ovaries and the testes, respectively, were categorized as miRNAs. Although only a small number of small RNAs in the gonads were identified as miRNAs, the number of miRNA species in the ovaries and testes was 591 and 548, respectively, which is similar to the number of miRNAs in somatic tissue. Among miRNA species, we identified 629 in fast muscle, 608 in slow muscle, 488 in heart, 539 in eye, 531 in brain, 519 in intestine, and 538 in liver. Our results suggest that many types of miRNA might be expressed in the gonads at a low level of expression. We also identified small RNA sequences as putative miRNAs that have many variations at the 3′ terminus compared with known miRNAs. These results show that, in every tissue, not more than 1 % of small RNAs was annotated as putative miRNAs. However, numbers of putative miRNA species were higher than those of miRNAs in every tissue except for the brain, and those in the ovaries and testes were much higher. The number of putative miRNAs was 1397 in fast muscle, 1242 in slow muscle, 532 in heart, 855 in eye, 508 in brain, 683 in intestine, 910 in liver, 2787 in ovaries, and 2651 in testes, indicating that various species of putative miRNA might be expressed in each tissue at a low level of expression. It may also be possible that some putative miRNAs might be other types of small RNA, fragment contaminants of longer RNAs, or novel miRNAs.

The RNAs of 26–31 nt that originated from repetitive elements were highly expressed in the ovaries and testes. These small RNAs may be piRNAs, considering their length and high abundance in gonads. Our results are similar to those of many previous studies; piRNAs that are 26–31 nt in size are mainly expressed in the ovaries and testes [15–20]. piRNAs are associated with PIWI proteins, which are members of the Argonaute family [19, 20]. piRNA precursors are transcribed from an intergenic repetitive element or a piRNA cluster [17, 21–23]. The main function of piRNAs is to maintain the integrity of the genome by silencing transposable elements [24, 25].

Considering the proportion of miRNAs of each length from 18 to 25 nt, we found that the brain generated more variation in miRNA sequence length than did the other tissues (Additional file 7). This is possibly because the brain is the most complex organ in the vertebrate body. The human brain comprises 10,000 difference types of neuron [26]. Therefore, a wide range of miRNA variants in the brain may be involved in brain function. Modifications of miRNAs at the 5′ terminus affect the seed region (2–8 nt), and may lead to differential target recognition [27–30]. Furthermore, 3′ modifications have been reported to be involved in the stability of miRNAs and the efficiency of target repression [31–33].

After constructing the miRNA expression profiles and validating the results with stem-loop RT-qPCR, we found that many miRNAs were ubiquitously expressed. For example, fru-miR-145-5p was highly expressed in every tissue. A previous study demonstrated that miR-145-5p plays an important role in enhancing smooth muscle cell differentiation and suppressing smooth muscle cell proliferation [34]. miR-145-5p targets and negatively regulates the pluripotency factors, OCT4, SOX2, and KLF4, in human embryonic stem cells [35]. In humans, miR-145-5p is highly abundant in the gonads and tissues arising from the mesoderm, such as the heart, spleen, uterus, and prostate [36]. However, some miRNAs exhibit tissue-specific expression patterns, suggesting that they play important roles in those tissues. Our results (highly expressed miRNAs in some tissues) are in agreement with previous studies. For example, fru-miR-1 was abundant in skeletal muscle and the heart, as it is in many animals. Previous studies have reported that in skeletal muscle, miR-1 is modulated by serum response factors (SRFs), such as MyoD and Mef2, which are muscle differentiation regulators [37]. miR-1 plays a role in repressing embryonic stem cell differentiation into non-muscle cells in mice and humans [38]. In the heart, miR-1 targets the transcription factor Hand2, which accelerates the expansion of the cardiac muscle [37]. In mice, over-expression of miR-1 inhibits muscle proliferation [39]. miR-1 also regulates the development of cardiac hypertrophy [40]. In addition, arrhythmogenesis in infarcted rat hearts can be treated by injection with an antisense strand of miR-1 [41].

Fru-miR-192-5p was the most abundant miRNA in the intestine. miR-192-5p belongs to the miR-192/215 family [10]. miR-192 and miR-215 share identical seed sequences, and only differ by two nucleotides [10]. They are tumor suppressor miRNAs, modulated by p53 (also a tumor suppressor gene), and they play a role in cell-cycle arrest to prevent tumorigenesis [42–44]. They are biomarkers that are down-regulated in colon cancer [42, 43]; however, both are up-regulated in gastric cancer, where they target the adhesion molecule ALCAM [45].

In this study, fru-miR-122 was a liver-specific miRNA. This result is similar to those from previous studies in other organisms, such as *Drosophila*, *C. elegans,* humans, and mice. The levels of miR-122 in blood plasma correlate with aminotransferase levels, which is an indicator of drug-induced liver injury [46]. Moreover, changes in miRNA levels can be detected earlier than changes in aminotransferase levels [46]. Therefore, miR-122 is a potential diagnostic and prognostic marker for liver pathology [46]. miR-122 is a tumor suppressor miRNA that negatively regulates *ADAM17*, resulting in the repression of hepatocellular carcinoma angiogenesis and metastasis [47]. Down-regulation of miR-122 can be used as a biomarker for hepatic cancer [48].

Some miRNAs exhibit special expression patterns. For example, fru-miR-202-5p exhibited much higher expression levels in the ovaries than in the somatic tissue, although the proportion of miRNAs in somatic tissue is much greater than that in the gonads, suggesting that they play crucial roles in germ cells. miR-202-5p exhibits sexual dimorphism during gonadal sex differentiation [49, 50]. In embryonic chickens, it is up-regulated during testes differentiation from the onset of testes development [51]. During development of the mouse testes, miR-202-5p is a direct transcriptional target of SOX9, a transcription factor that plays a role in male sex determination [50]. It is highly expressed in Sertoli cells when the male primordial germ cells differentiate into the testes [50]. However, in our study, fru-miR-202-5p was detected in both the ovaries and the testes because our sample was at a mature stage, a result that is consistent with previous studies; miR-202-5p is a postnatal gonad-specific miRNA in Atlantic halibut (*Hippoglossus hippoglossus*), *Xenopus*, mice, pigs, and humans [52–56]. These results suggest that miR-202-5p has an important function that involves sex determination during testes development, and also plays a crucial role in postnatal ovaries and testes [50].

Most of the miRNA species exhibited variation at the 3′ terminus, while variation at the 5′ terminus was not as prevalent as that at the 3′ terminus [29]. Shifts in the seed region (nucleotide positions 2–8 from the 5′ terminus) lead to differential target recognition and a role in gene regulation [27–30]. In general, the major variant of each miRNA species was common to all the tissues, but for some miRNA species the most abundant variants had different seed sequences among the nine tissues, suggesting that miRNAs may have different targets and functions in different tissues.

In this study, we identified many miRNAs in *T. rubripes*. To validate the putative piRNAs, more information regarding associated proteins [19, 20] and examination of nucleotide bias at the specific positions are required [19, 21, 22]. Our data also showed other classes of small RNAs that may have important, but yet unidentified, functions.

## Conclusions

In this study, we investigated miRNA expression patterns in various organs of *T. rubripes*. We identified 1420 known miRNA species. Many miRNAs exhibited conserved tissue-specific expression patterns, indicating that their basic functions share a common evolution among vertebrates. miRNAs were mainly expressed in tissues other than the gonads, whereas putative piRNAs were highly abundant in the ovaries and testes; however, some miRNA species were highly abundant in the gonads. The most abundant isomiRs of specific miRNAs may have different functions in different tissues. Our data may also include new kinds of small RNAs. Our data may also include new kinds of small RNAs.

## Methods

### Preparation and sequencing of small RNA libraries

All procedures in this study were performed according to the Animal Experimental Guidelines of the Ethical Committee of The University of Tokyo. Total RNA was extracted from 0.1 g of each tissue (fast and slow muscle, heart, eye, brain, intestine, liver, ovaries, and testes dissected from two individual adult *T. rubripes*) that had been stored in RNA*later* solution for 1 h (Applied Biosystems, Foster City, CA, USA). Total RNA was extracted using a mirVana miRNA Isolation Kit (Applied Biosystems). To purify small RNA, total RNA was fractionated through a flashPAGE Fractionator (Applied Biosystems), and small RNA was retrieved. Nine small RNA libraries were constructed, using Small RNA Library Preparation for SOLiD Sequencing (Applied Biosystems). The entire libraries were sequenced using the SOLiD 3 next-generation sequencer (Applied Biosystems).

### Small RNA data annotation

After the acquisition of small RNA data from the sequencer, low-quality reads and 3′ sequencing adapters were eliminated and trimmed, respectively, by our own Perl script. However, some adapter sequences were shorter than 4 nt, and we could not judge whether they were the real adaptor sequences or belonged to the 3′ part of small RNA sequences. We decided not to remove the short sequences; therefore, many small RNAs were 35 nt in length. In addition, the population of 32–34 nt sequences was absent after removal of the adaptor. Small RNAs (18–25 nt) were BLAST-searched against mature miRNAs deposited in miRBase 19.0 (www.mirbase.org/), using CLC genomics workbench software (CLC bio, Aarhus, Denmark). The sequences with non-base pair changes, 1–2 nt extended or shortened at the 5′ end, or 1–4 nt extended or shortened at the 3′ end were annotated as miRNAs. Owing to 3′ variations or sequence errors, many small RNAs could not be annotated. Therefore, we separated reads that had identical nucleotides in positions 1–10 with known miRNAs and performed downstream analysis. Sequences that had an alignment length that was equal to or more than 16 nt with known miRNAs were regarded as miRNAs. In cases where small RNAs had an alignment length of less than 16 nt, we used the Smith-Waterman score in FASTA v36 (http://faculty.virginia.edu/wrpearson/fasta/fasta36/) to evaluate the alignment. The parameter was set to add ‘5’ and ‘−4’ scores for match and mismatch cases, respectively. We identified sequences with a score equal to or more than 70 as miRNAs. We identified sequences that had a Smith-Waterman score of less than 70 as putative miRNAs.

### miRNA expression profile

The expression levels of individual miRNAs were calculated as RPM (read per million) and compared among the tissues. The individual miRNA expression level (RPM) was calculated as: (the number of reads of the individual miRNA/total number of 18–25 nt small RNA reads in the tissue) × 10^6^.

### Data accession numbers

Sequence data sets used in this study were deposited in the GEO database [57] under the accession number GSE65404.

### miRNA Stem loop real time PCR

We choose stem-loop real-time PCR for miRNA quantification. This method is more specific than linear PCR as stem-loop primers are designed to be complementary to the 6 nt of 3′ termini of mature miRNAs [58]. First, 1 μg of total RNA from fast and slow muscles, heart, eye, brain, intestine, liver (all *n* = 3), ovaries, and testes (both *n* = 2) were used for first-strand cDNA synthesis. Stem loop primer was added to total RNA (Additional file 14) and reversed transcribed using Superscript III (Invitrogen). Subsequently, cDNA was used as a template for real-time PCR. The PCR mixture contained EXPRESS SYBR GreenER qPCR SuperMix Universal 10 μL (Invitrogen), 0.4 μL of 10 μM miRNA-specific forward and universal reverse primer (Additional file 14), 0.4 μL of ROX Reference Dye (25 μM), and was made up to 20 μL with nuclease free water. Reactions were performed on an Applied Biosystems 7300 Real-Time PCR System. Each sample was run in triplicate. miRNA expression was normalized against that of *T. rubripes* 5 s ribosomal RNA (Additional file 14).

## Additional files

Additional file 1: Figure S1. Small RNA data pipeline analysis. The diagram represents the data pipeline used to analyze data from next-generation sequencing and the miRNA annotation criteria. Additional file 2: Table S1. Number of small RNA reads at each step of the data pipeline analysis. The number of small RNAs retrieved after passing small RNA reads through each step of the data pipeline analysis Additional file 3: Table S2. List of miRNAs and their read numbers in each tissue of *T. rubripes*. The order of miRNAs is based on their expression levels after normalizing reads against 1 million 18–25 nt small RNAs. Additional file 4: Table S3. List of putative miRNAs and their read numbers in each tissue of *T. rubripes*. The order of putative miRNAs is based on their expression levels after normalizing reads against 1 million 18–25 nt small RNAs. Additional file 5: Figure S2. Number of miRNA and putative miRNA species found in each tissue. Additional file 6: Figure S3. Size distribution patterns of miRNAs and putative miRNAs in each tissue. The size distribution patterns of 18–25 nt miRNAs (A) and putative miRNAs (B) reads after normalizing against 1 million 18–25 nt small RNAs, from nine tissues of *T. rubripes*. Additional file 7: Figure S4. Ratio of reads of each 18–31 nt RNA species identified in this study to total small RNA reads in each tissue. The ratios of miRNAs, putative miRNAs, mRNAs, tRNA/rRNA/snRNAs, other non-coding RNAs, repeats, reads that failed to align, others and unidentified small RNAs of 18–31 nt to total small RNA reads. Additional file 8: Figure S5. miRNA proportions as percentages of 1–35 nt small RNAs. Proportions of 1–17 nt small RNAs, 18–25 nt miRNAs, 18–25 nt putative miRNAs, 18–25 nt unidentified small RNAs, 26–31 nt small RNAs, and 32–35 nt small RNAs are shown as percentages of total 1–35 nt small RNAs. Additional file 9: Table S4. Expression profiles of miRNAs and putative miRNAs in *T. rubripes* in all tissues examined. List of known miRNAs and putative miRNAs, their most abundant sequences, and their read numbers, after normalization against reads with 1 million 18–25 nt small RNAs in each tissue. Additional file 10: Figure S6. Proportions of isomiR variation types as percentages of total fru-miR-145-5p in each tissue. Proportions of the isomiRs that exhibited nucleotide variation only in the 3′ region, the isomiRs that exhibited variation at both the 3′ and 5′ ends, the isomiRs that exhibited only 5′ variation, and the major sequence of the isomiRs are shown as percentages of total fru-miR-145-5p. Additional file 11: Figure S7. IsomiRs of fru-miR-462-5p. The diagrams represent repertoires of fru-miR-462-5p isomiRs mapped to their miRNA precursors in each tissue. Additional file 12: Figure S8. IsomiRs of fru-miR-101b-3p. The diagrams represent repertoires of fru-miR-101b-3p isomiRs mapped to their miRNA precursors in each tissue. Additional file 13: Figure S9. IsomiRs of fru-miR-133-3p. The diagrams represent repertoires of fru-miR-133-3p isomiRs mapped to their miRNA precursors in each tissue. Additional file 14: Table S5. Primers for RT-qPCR. List of stem loop primers and miRNA gene-specific primers, including internal control forward primers and a universal reverse primer.
