# Supplementary material for: Deep sequencing, profiling and detailed annotation of microRNAs in Takifugu rubripes
Source: BMC Genomics. 2015 Jun 16;16(1):457. doi: 10.1186/s12864-015-1622-1 (PMC4469249; doi:10.1186/s12864-015-1622-1)
Supplement: Additional file 1: Figure S1. — Small RNA data pipeline analysis. The diagram represents the data pipeline used to analyze data from next-generation sequencing and the miRNA annotation criteria. [file 12864_2015_1622_MOESM1_ESM.pdf]

Additional file 1: Figure S1

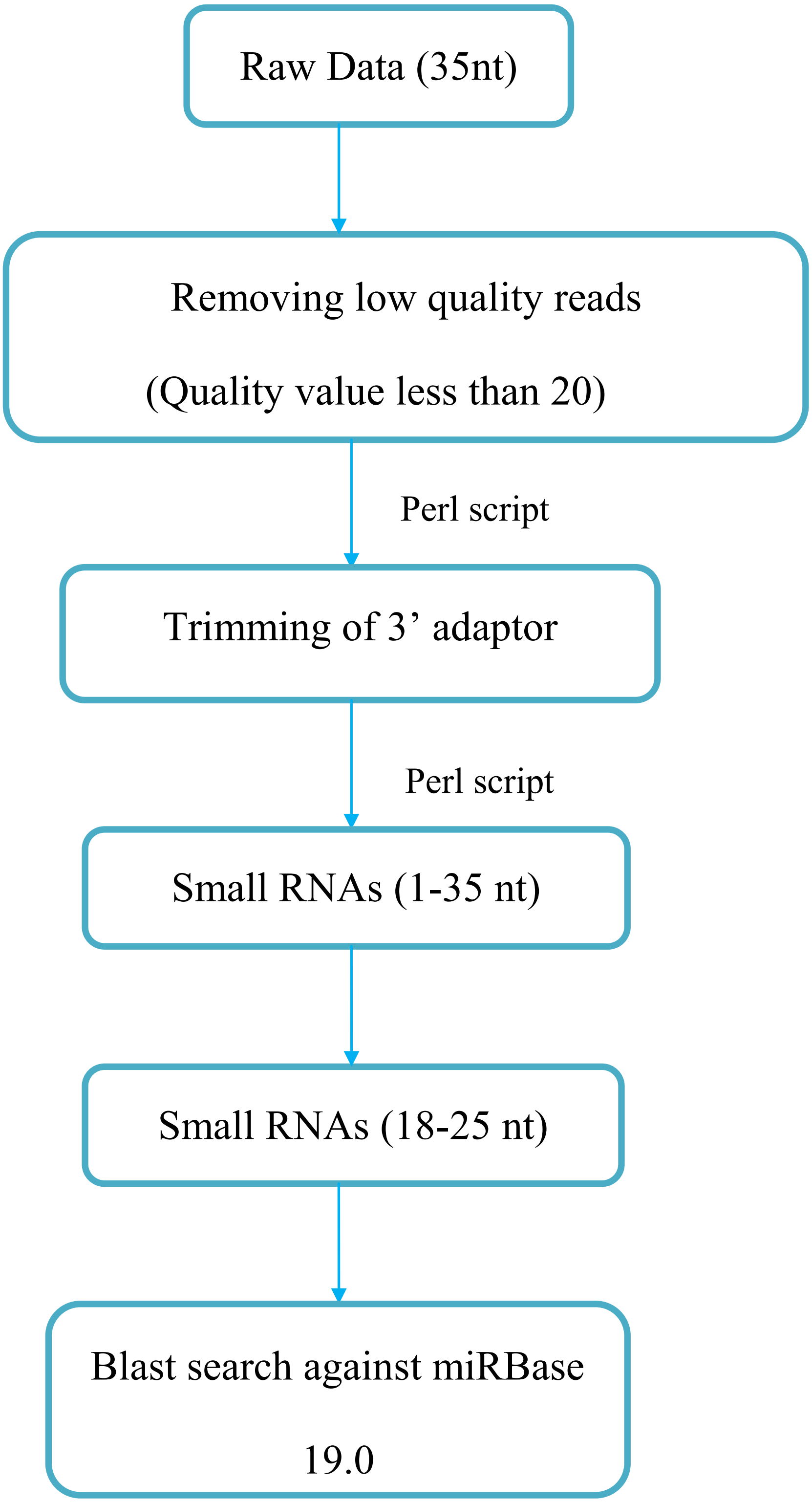

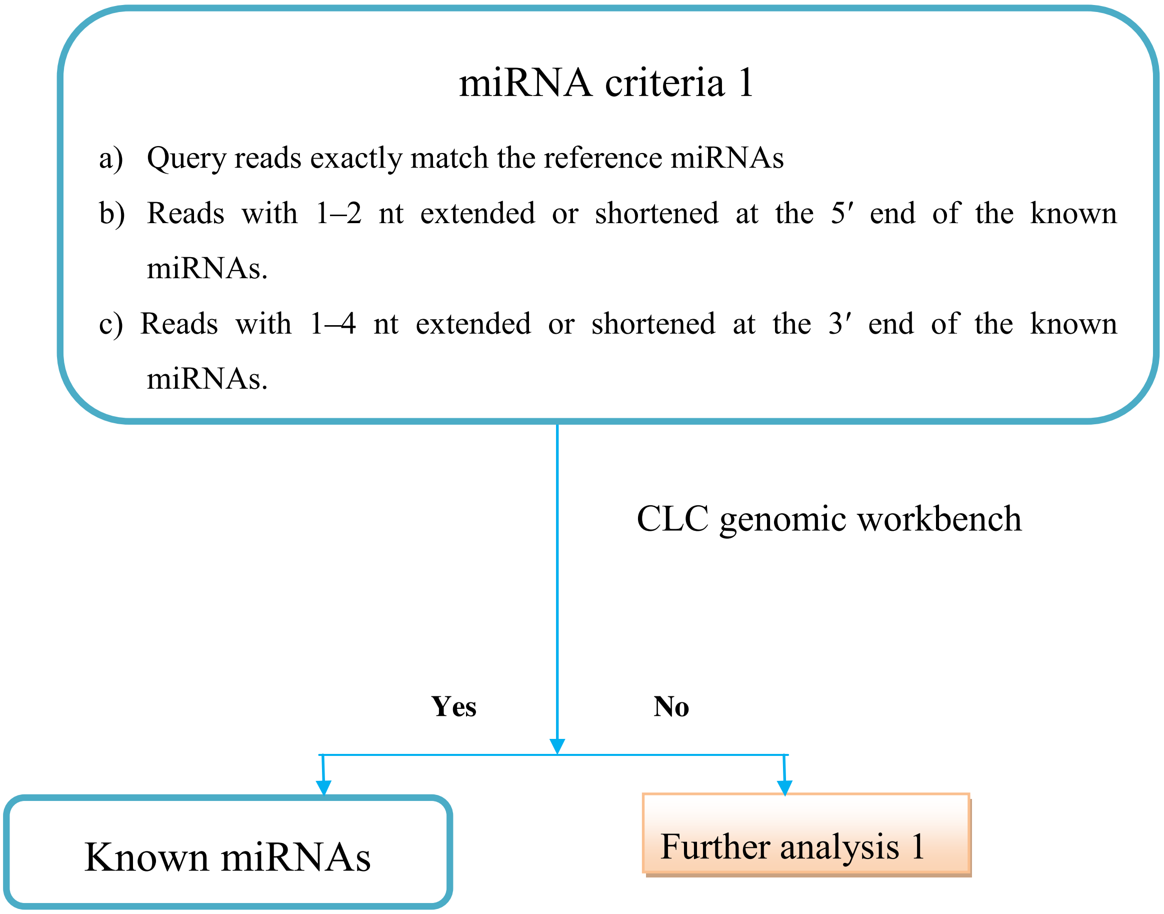
miRNA criteria 1
a) Query reads exactly match the reference miRNAs
b) Reads with 1–2 nt extended or shortened at the 5′ end of the known miRNAs.
c) Reads with 1–4 nt extended or shortened at the 3′ end of the known miRNAs.
CLC genomic workbench
Yes
No
Known miRNAs
Further analysis 1

miRNA criteria 1

a) Query reads exactly match the reference miRNAs

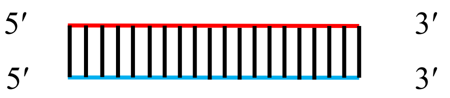

b) Reads with 1–2 nt extended or shortened at the 5′ end of the known miRNAs.

extended

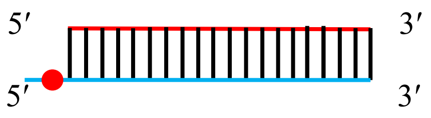

shortened

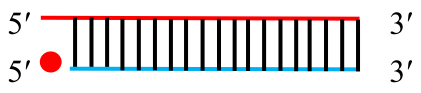

c) Reads with 1–4 nt extended or shortened at the 3′ end of the known miRNAs.

extended

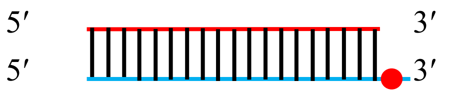

shortened

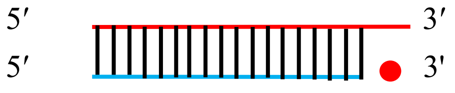

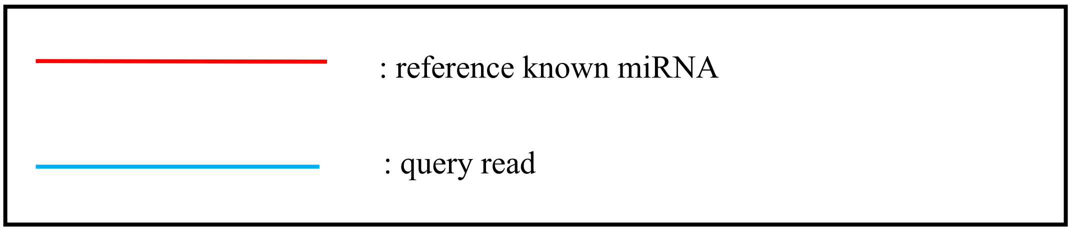

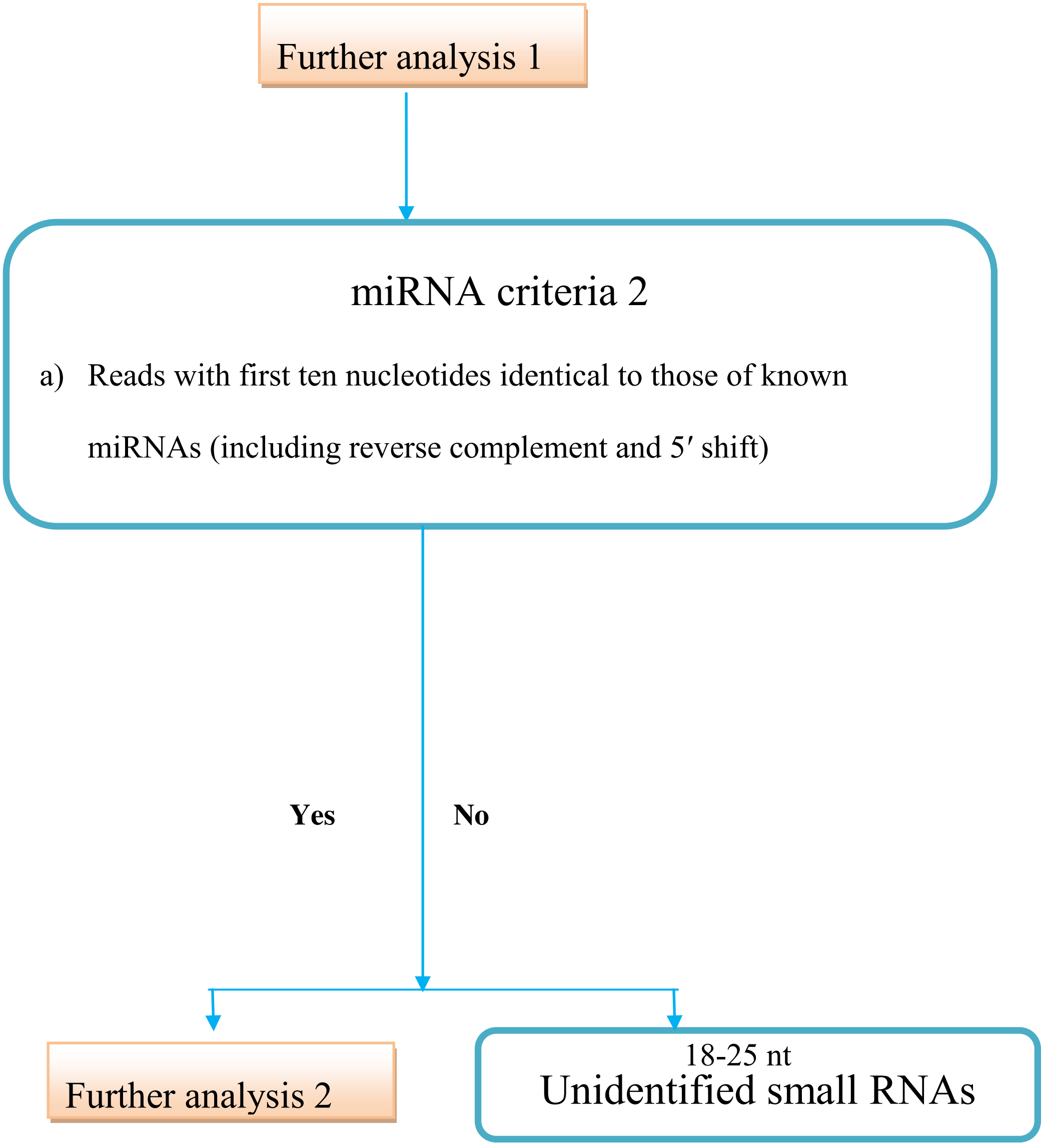
Further analysis 1
miRNA criteria 2
a) Reads with first ten nucleotides identical to those of known miRNAs (including reverse complement and 5′ shift)
Yes
No
Further analysis 2
18-25 nt
Unidentified small RNAs

miRNA criteria 2

a) Reads with first ten nucleotides identical to those of known miRNAs (including reverse complement and 5′ shift)

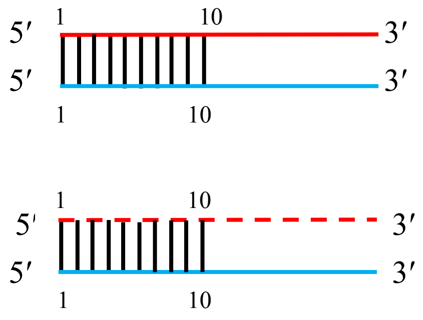

5′ shift

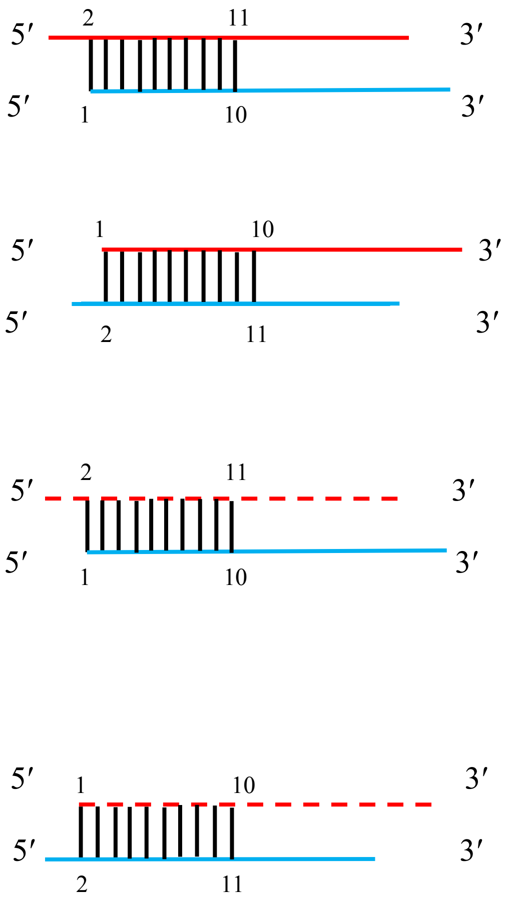

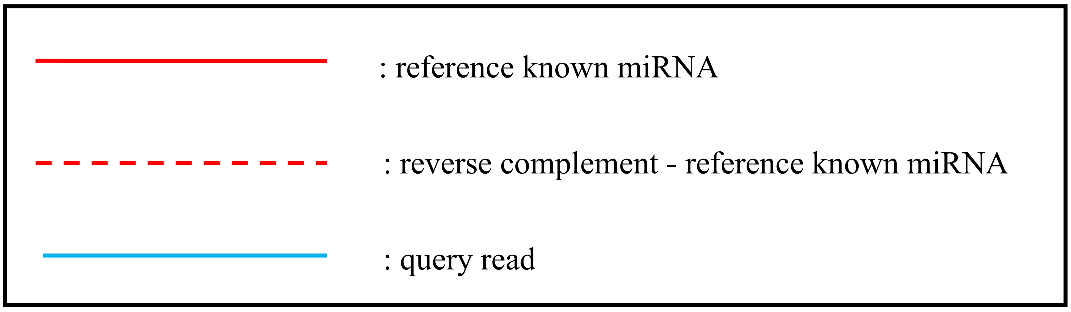

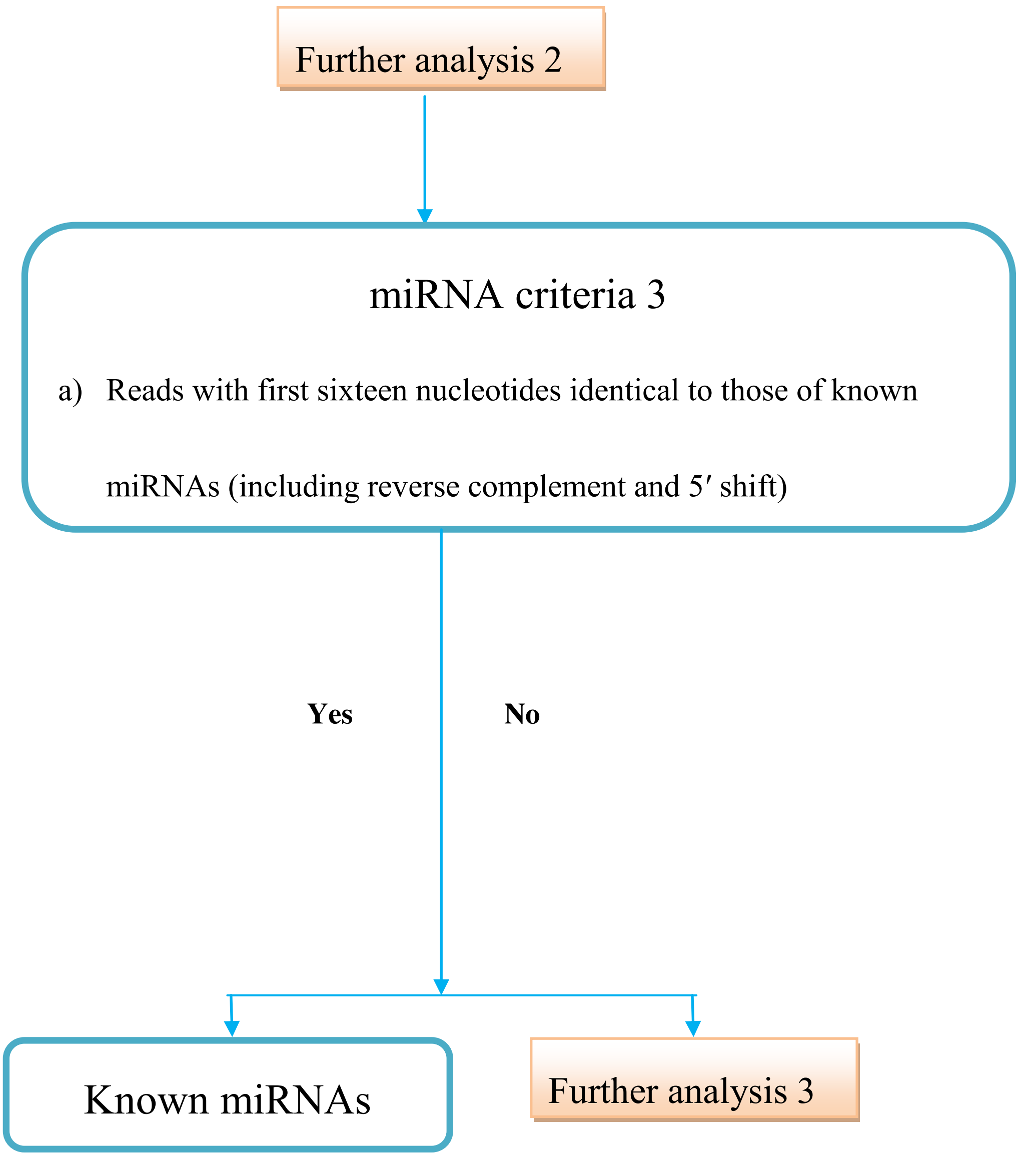
Further analysis 2
miRNA criteria 3
a) Reads with first sixteen nucleotides identical to those of known miRNAs (including reverse complement and 5′ shift)
Yes
No
Known miRNAs
Further analysis 3

miRNA criteria 3

a) Reads with first sixteen nucleotides identical to those of known miRNAs (including reverse complement and 5′ shift)

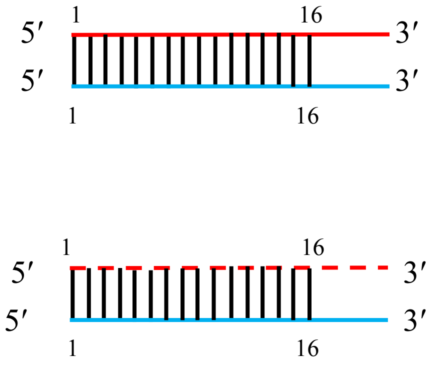

5′ shift

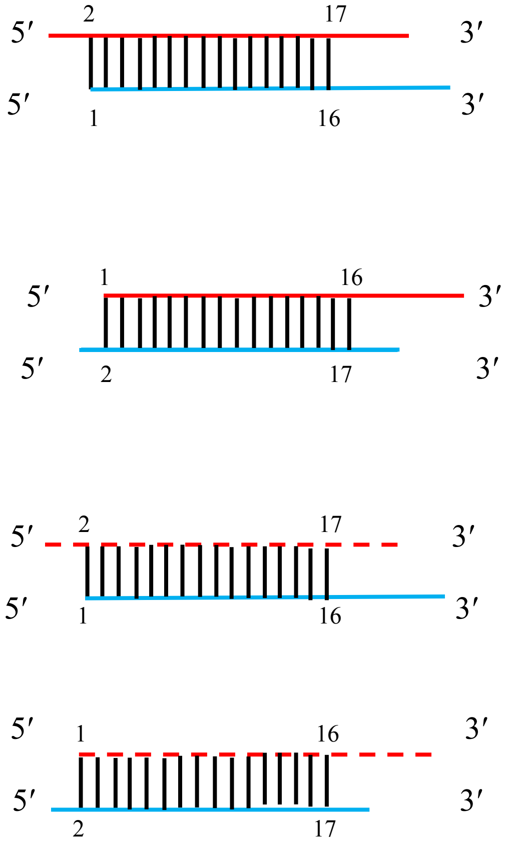

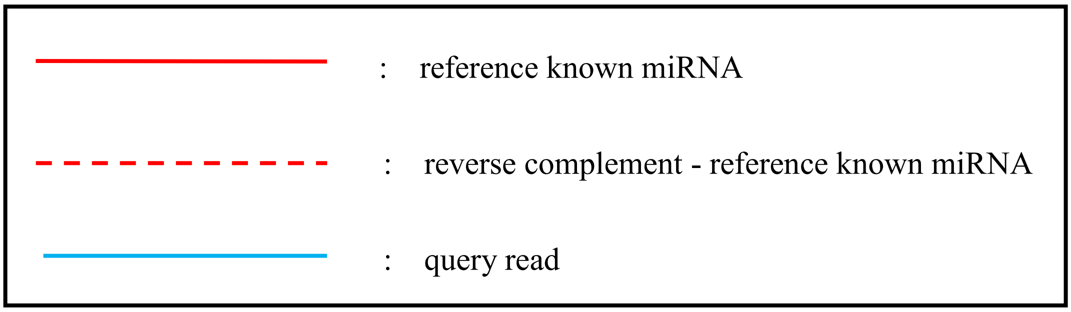

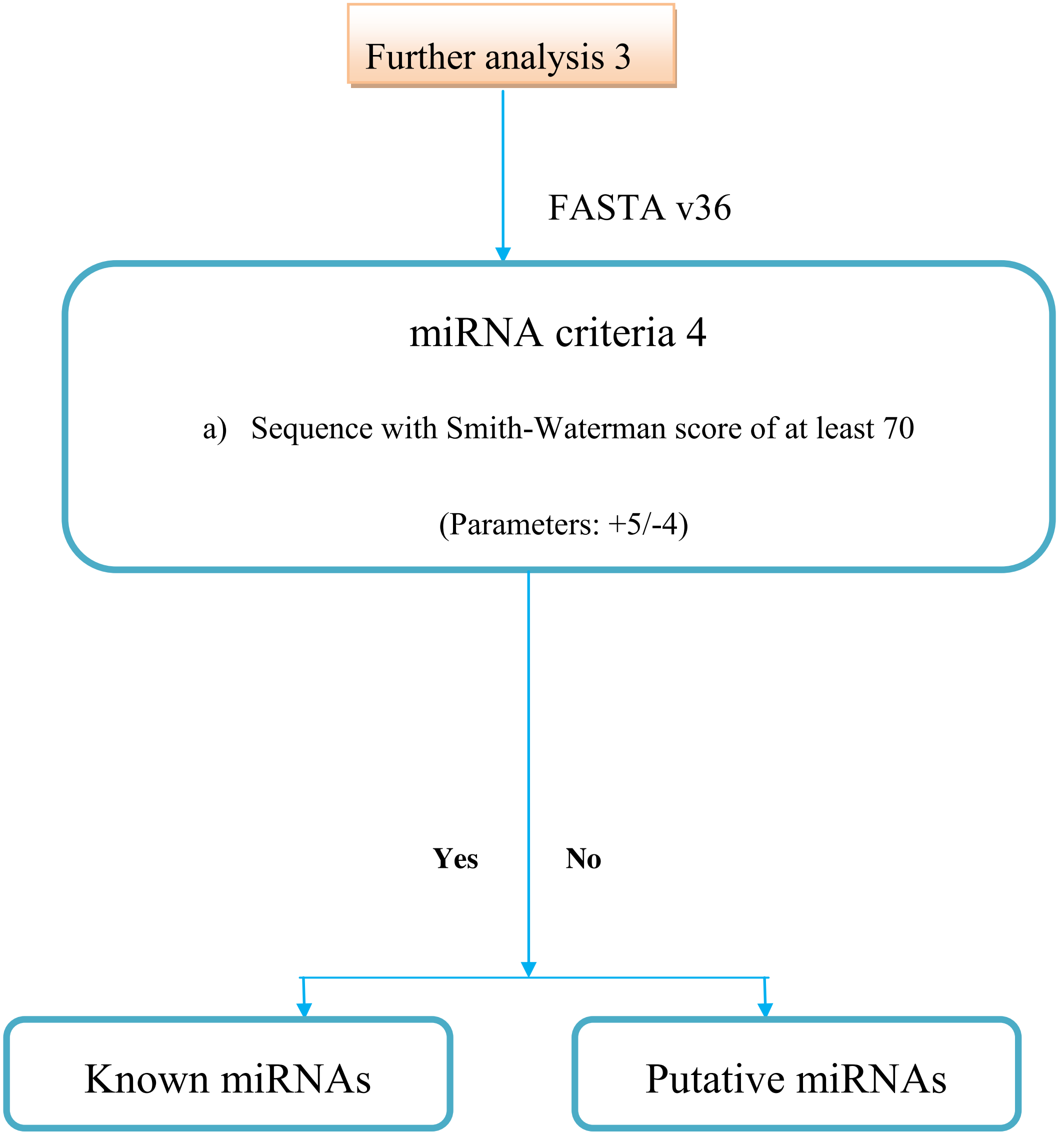
Further analysis 3
FASTA v36
miRNA criteria 4
a) Sequence with Smith-Waterman score of at least 70
(Parameters: +5/-4)
Yes
No
Known miRNAs
Putative miRNAs
