# Supplementary material for: Deep sequencing, profiling and detailed annotation of microRNAs in Takifugu rubripes
Source: BMC Genomics. 2015 Jun 16;16(1):457. doi: 10.1186/s12864-015-1622-1 (PMC4469249; doi:10.1186/s12864-015-1622-1)

Additional file 5: Figure S2

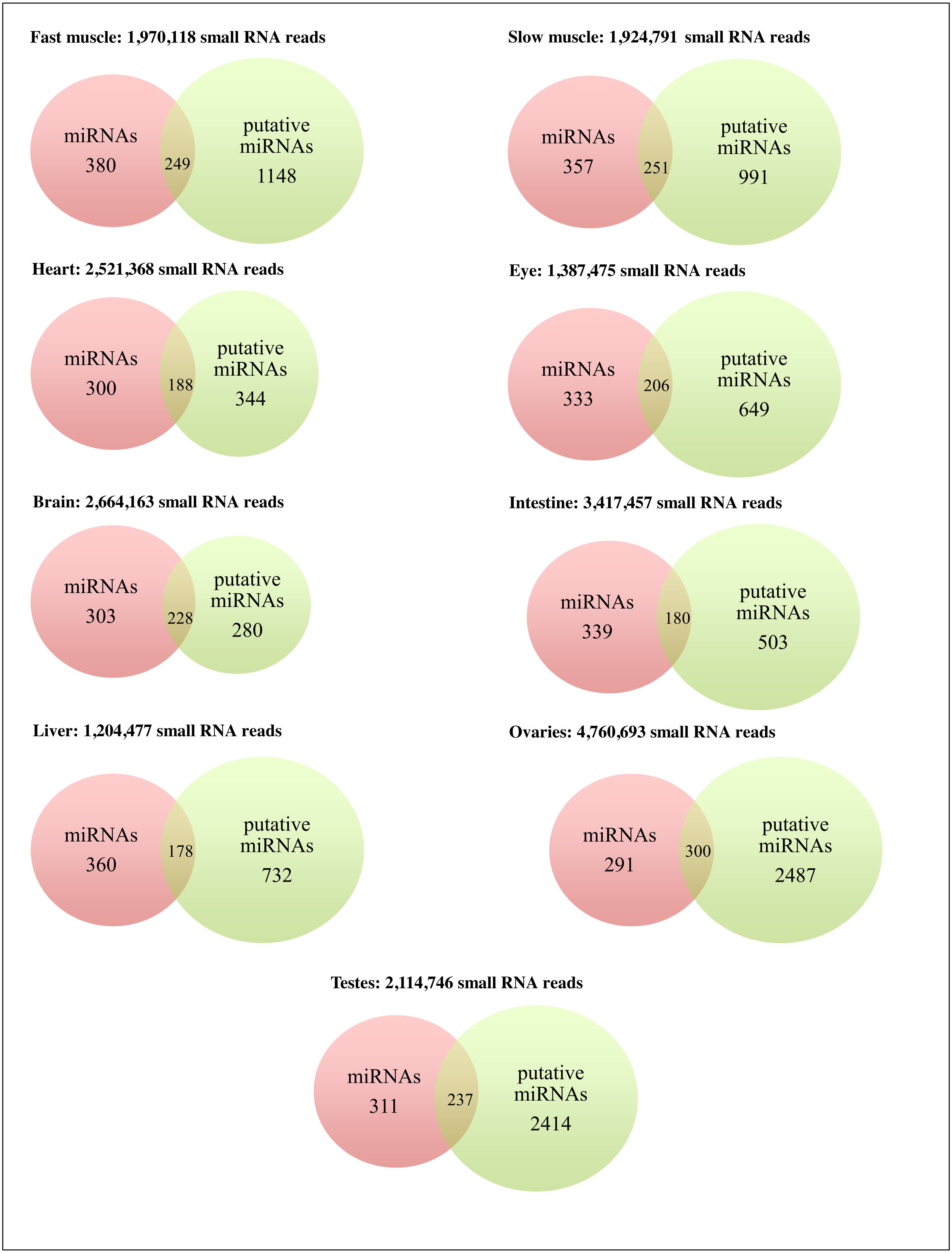

Supplement: Additional file 5: Figure S2. — Number of miRNA and putative miRNA species found in each tissue. [file 12864_2015_1622_MOESM5_ESM.pdf]
