# Supplementary material for: Deep sequencing, profiling and detailed annotation of microRNAs in Takifugu rubripes
Source: BMC Genomics. 2015 Jun 16;16(1):457. doi: 10.1186/s12864-015-1622-1 (PMC4469249; doi:10.1186/s12864-015-1622-1)
Supplement: Additional file 6: Figure S3. — Size distribution patterns of miRNAs and putative miRNAs in each tissue. The size distribution patterns of 18–25 nt miRNAs (A) and putative miRNAs (B) reads after normalizing against 1 million 18–25 nt small RNAs, from nine tissues of T. rubripes. [file 12864_2015_1622_MOESM6_ESM.pdf]

Additional file 6: Figure S3

A) miRNAs reads

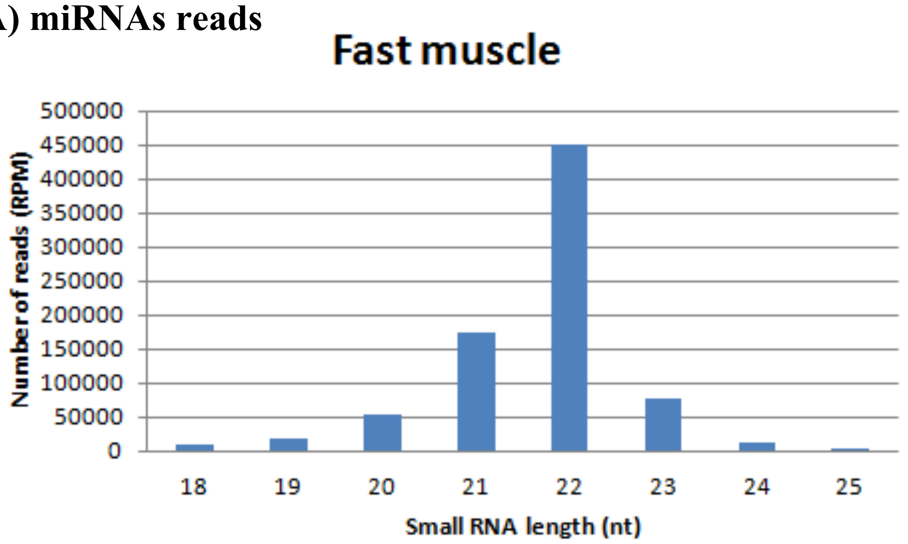

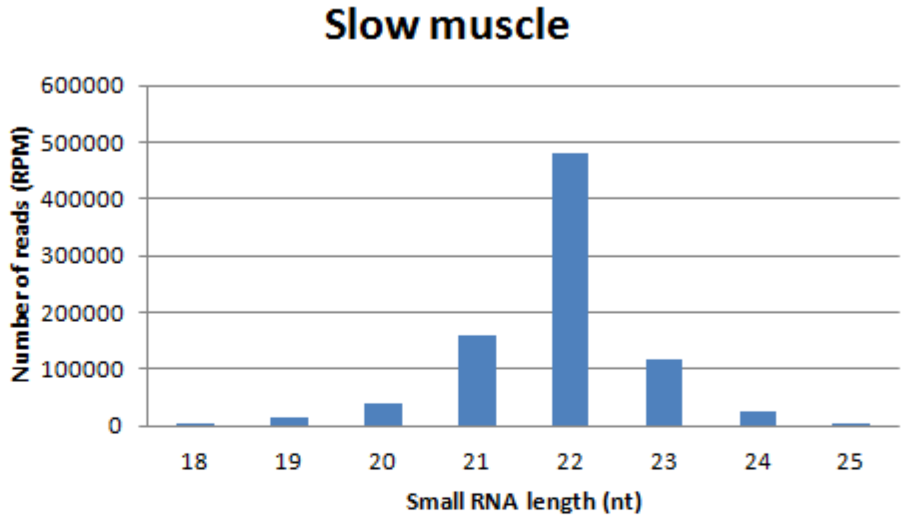

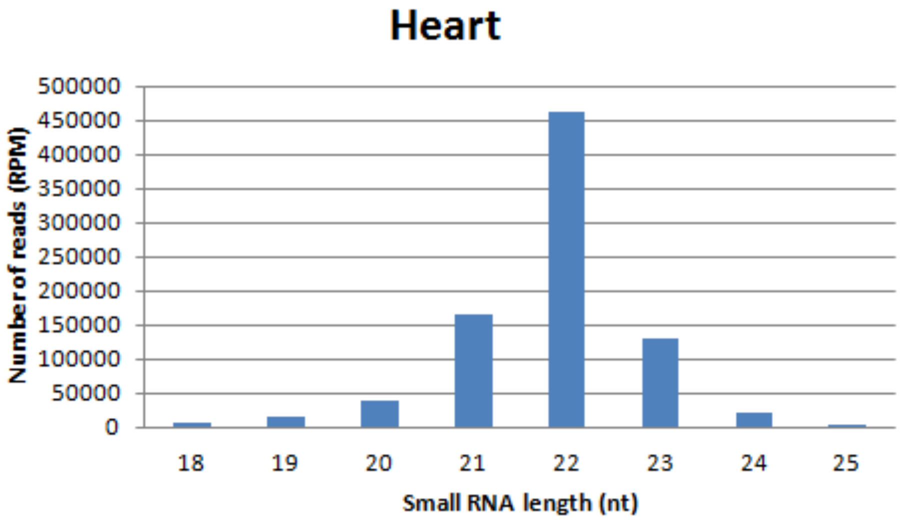

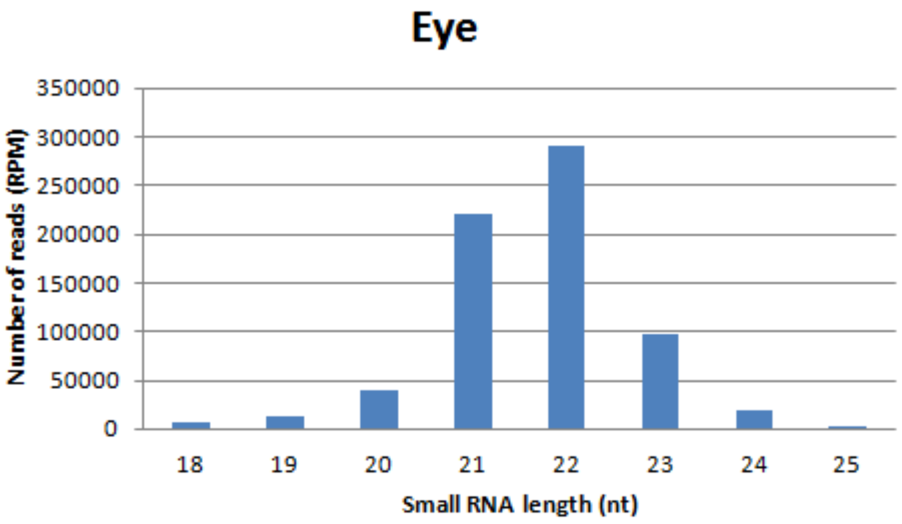
Eye
350000
300000
250000
200000
150000
100000
50000
0
Number of reads (RPM)
18
19
20
21
22
23
24
25
Small RNA length (nt)

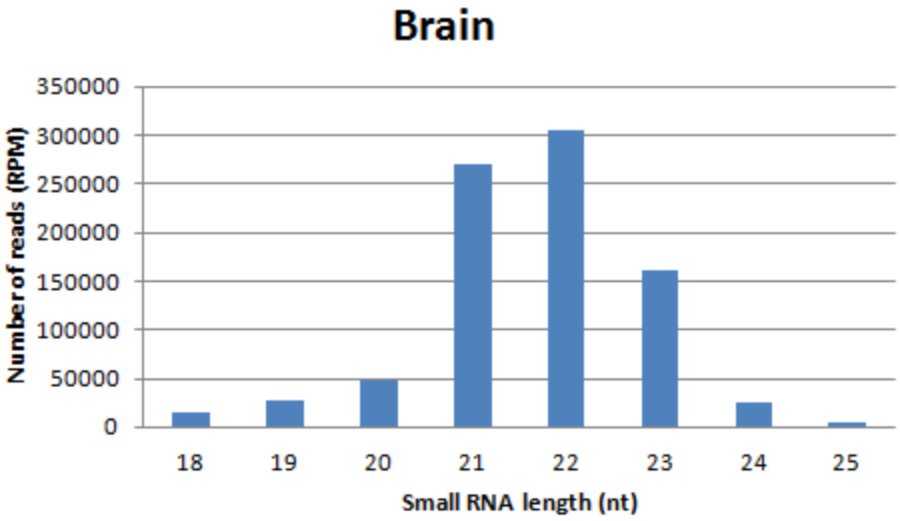
Brain
350000
300000
250000
200000
150000
100000
50000
0
Number of reads (RPM)
18
19
20
21
22
23
24
25
Small RNA length (nt)

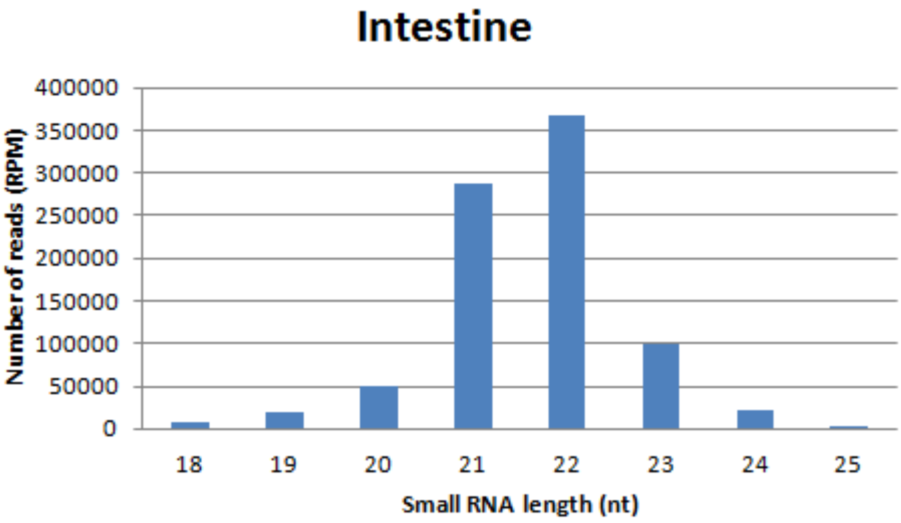
Intestine
400000
350000
300000
250000
200000
150000
100000
50000
0
Number of reads (RPM)
18
19
20
21
22
23
24
25
Small RNA length (nt)

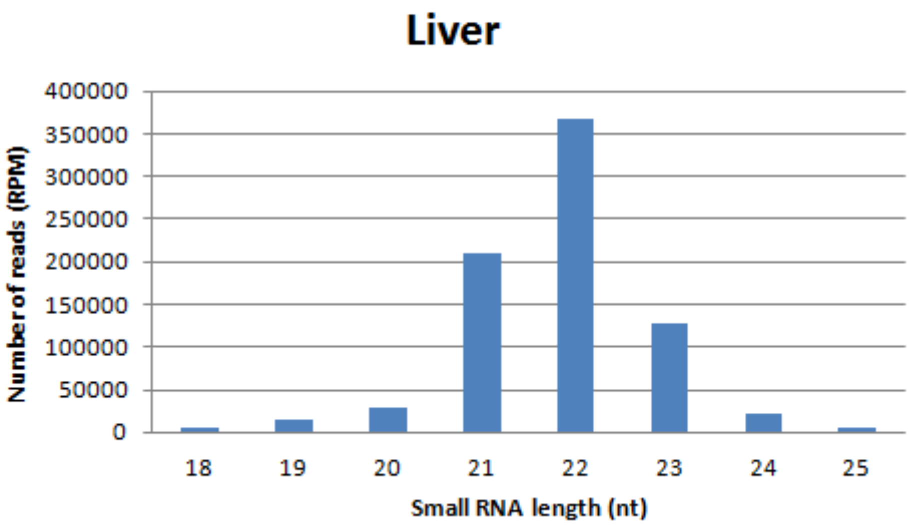
Liver
400000
350000
300000
250000
200000
150000
100000
50000
0
Number of reads (RPM)
18
19
20
21
22
23
24
25
Small RNA length (nt)

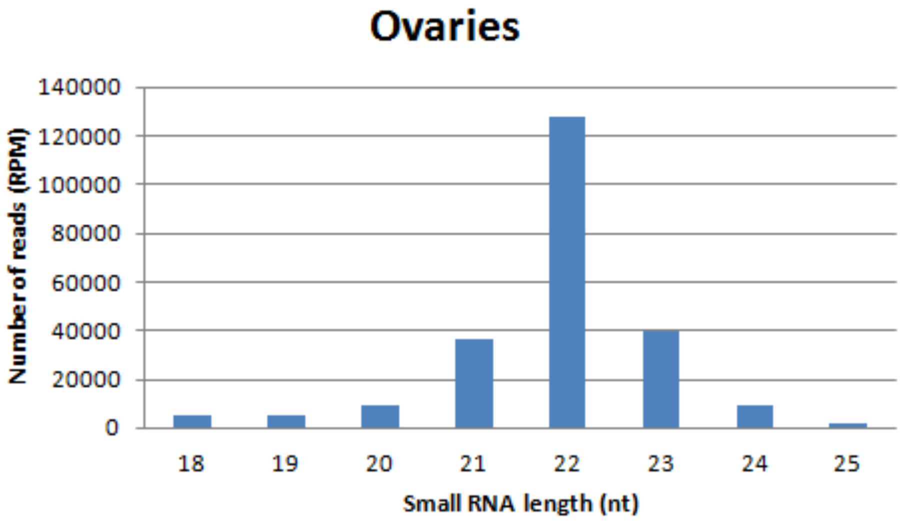
Ovaries
140000
120000
100000
80000
60000
40000
20000
0
Number of reads (RPM)
18
19
20
21
22
23
24
25
Small RNA length (nt)

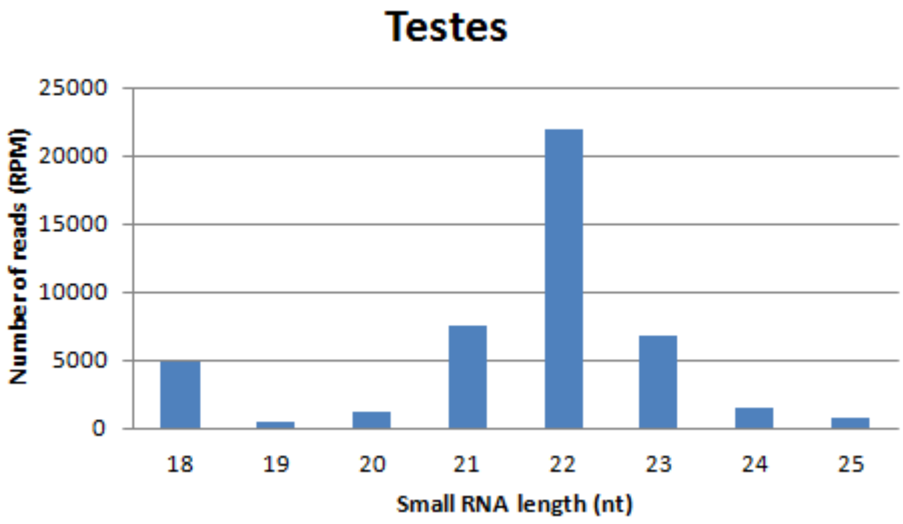
Testes
25000
20000
15000
10000
5000
0
Number of reads (RPM)
18
19
20
21
22
23
24
25
Small RNA length (nt)

B) Putative miRNAs reads

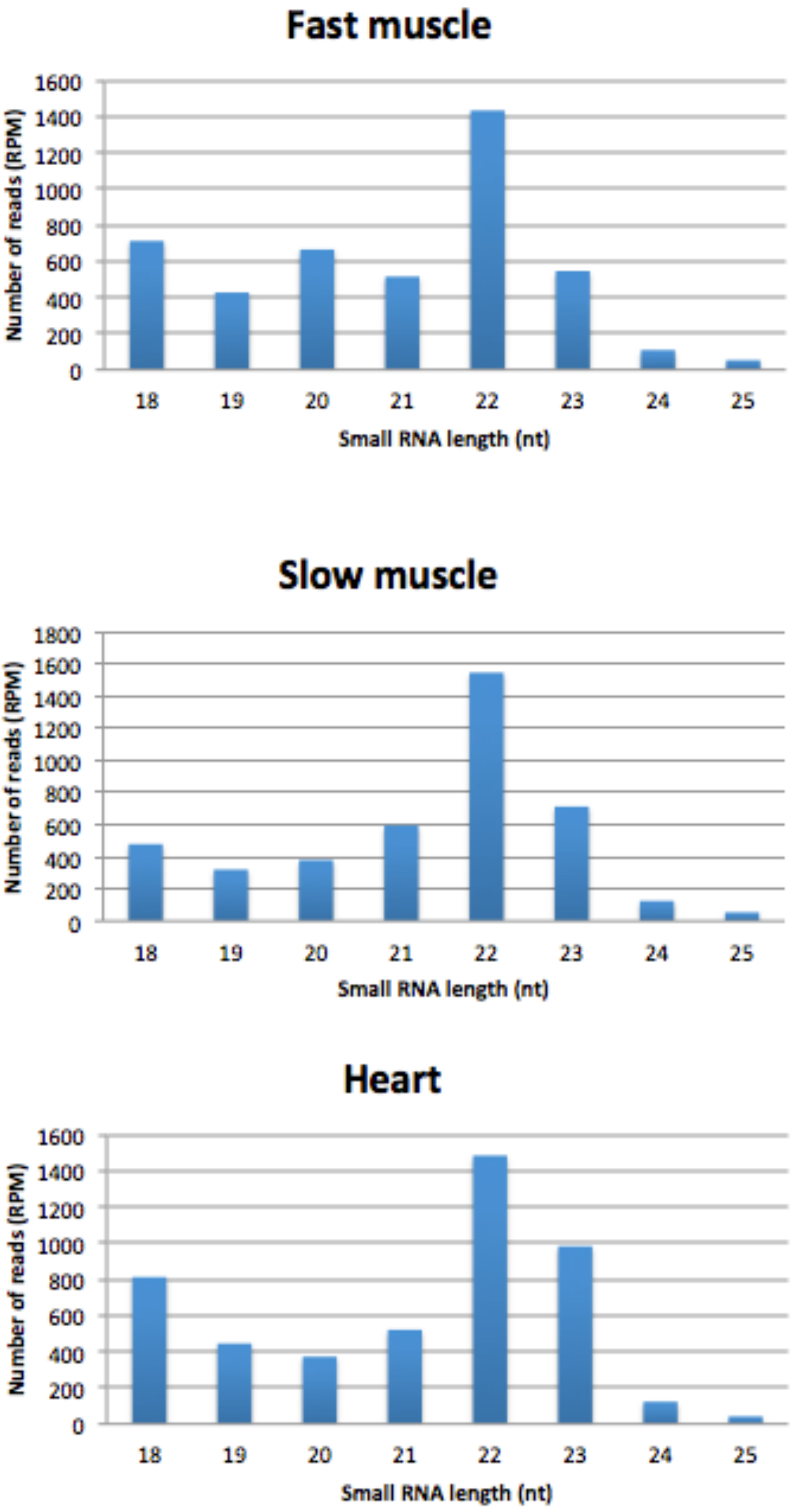

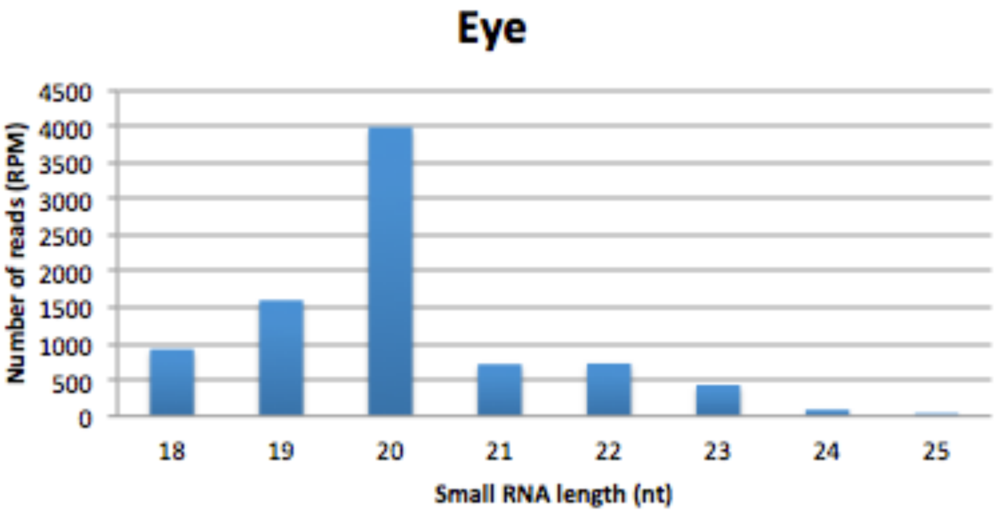
Eye
Number of reads (RPM)
4500
4000
3500
3000
2500
2000
1500
1000
500
0
18
19
20
21
22
23
24
25
Small RNA length (nt)

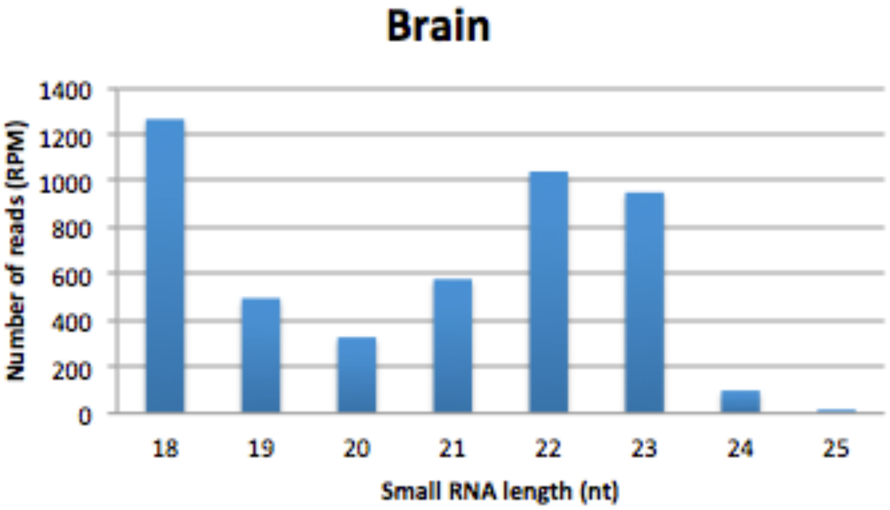
Brain
Number of reads (RPM)
1400
1200
1000
800
600
400
200
0
18
19
20
21
22
23
24
25
Small RNA length (nt)

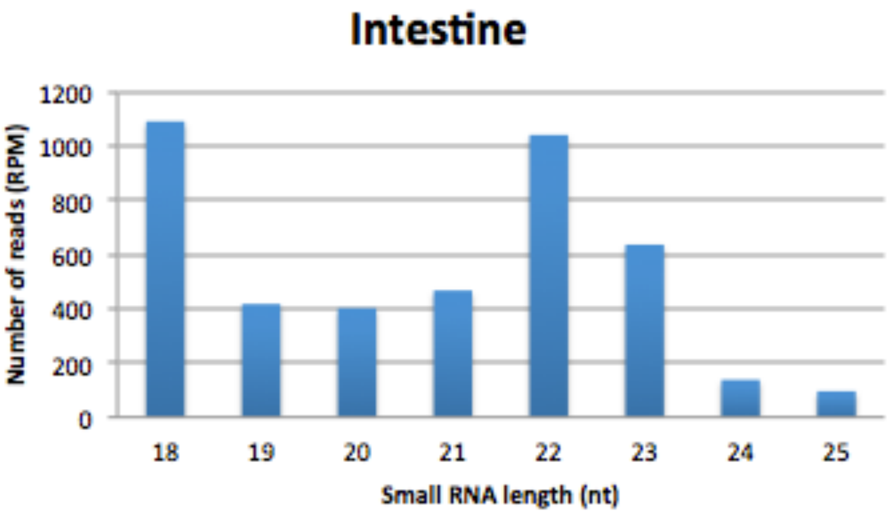
Intestine
Number of reads (RPM)
1200
1000
800
600
400
200
0
18
19
20
21
22
23
24
25
Small RNA length (nt)

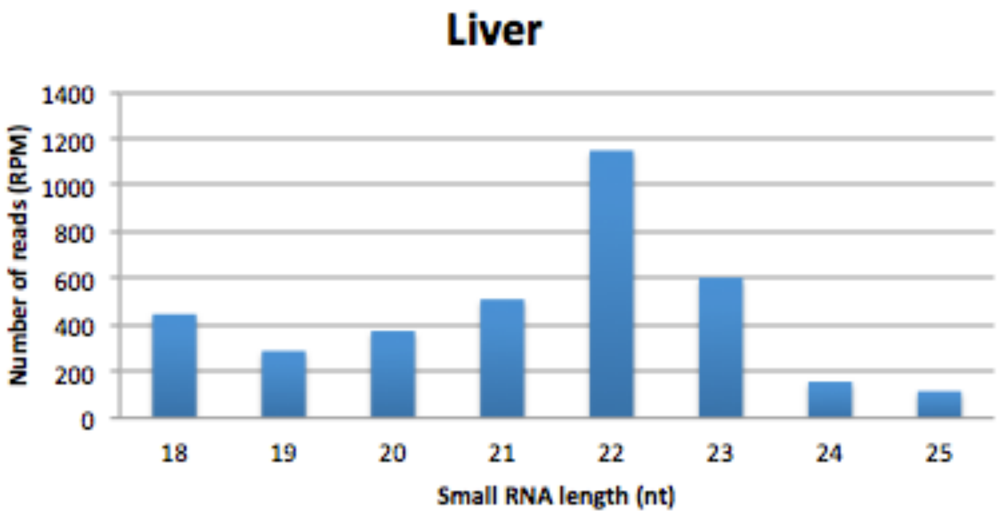
Liver
Number of reads (RPM)
1400
1200
1000
800
600
400
200
0
18
19
20
21
22
23
24
25
Small RNA length (nt)

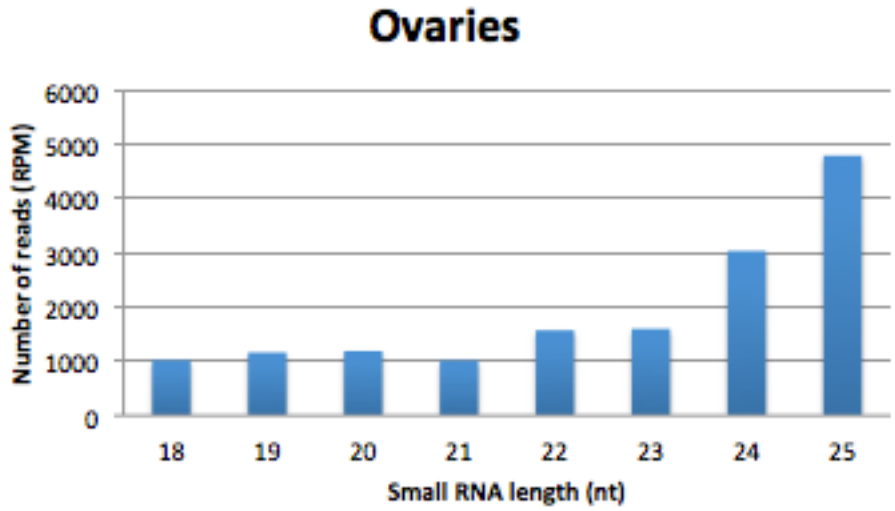
Ovaries
Number of reads (RPM)
6000
5000
4000
3000
2000
1000
0
18
19
20
21
22
23
24
25
Small RNA length (nt)

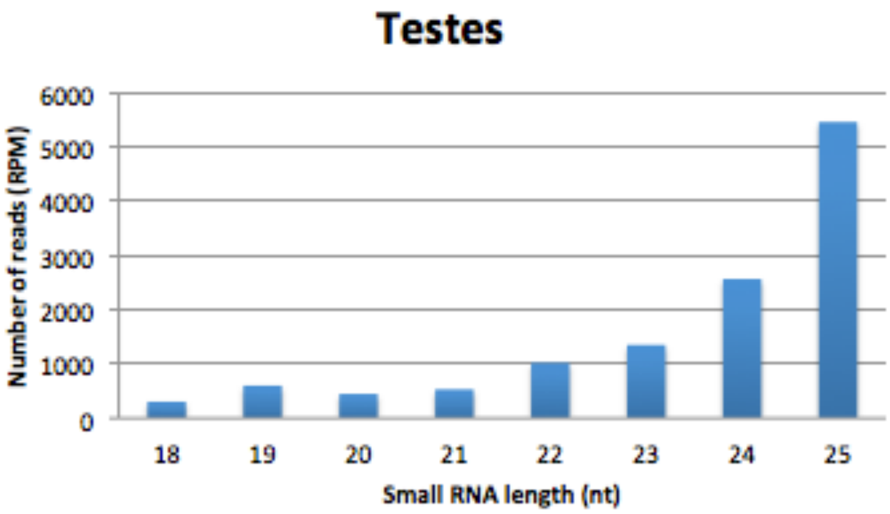
Testes
Number of reads (RPM)
6000
5000
4000
3000
2000
1000
0
18
19
20
21
22
23
24
25
Small RNA length (nt)
