# Supplementary material for: Deep sequencing, profiling and detailed annotation of microRNAs in Takifugu rubripes
Source: BMC Genomics. 2015 Jun 16;16(1):457. doi: 10.1186/s12864-015-1622-1 (PMC4469249; doi:10.1186/s12864-015-1622-1)
Supplement: Additional file 7: Figure S4. — Ratio of reads of each 18–31 nt RNA species identified in this study to total small RNA reads in each tissue. The ratios of miRNAs, putative miRNAs, mRNAs, tRNA/rRNA/snRNAs, other non-coding RNAs, repeats, reads that failed to align, others and unidentified small RNAs of 18–31 nt to total small RNA reads. [file 12864_2015_1622_MOESM7_ESM.pdf]

Additional file 7: Figure S4

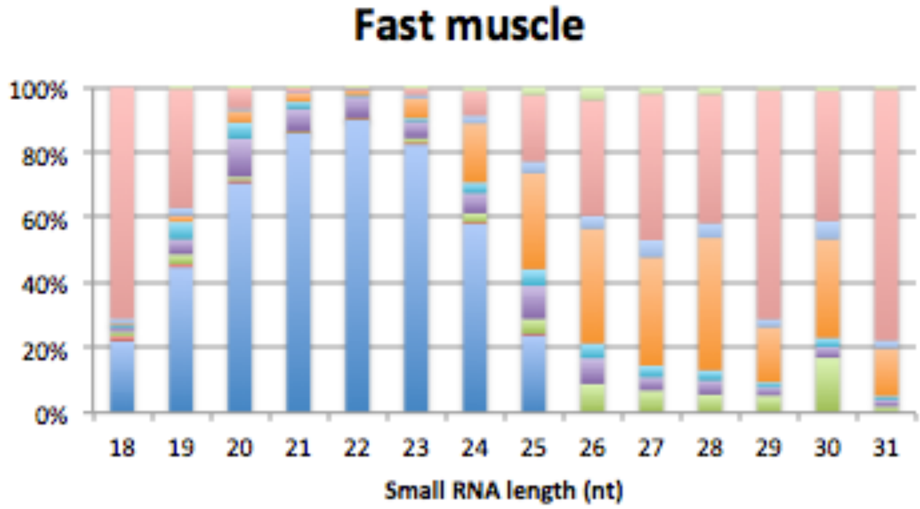

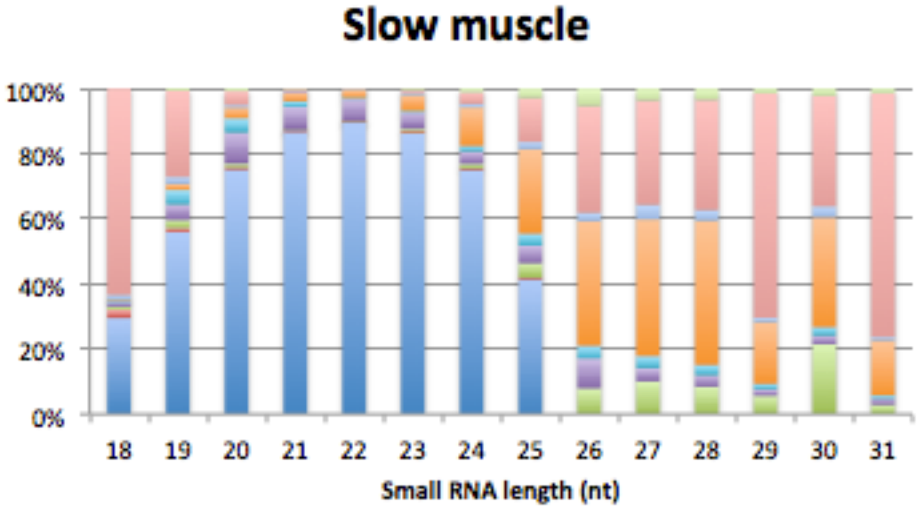

Heart

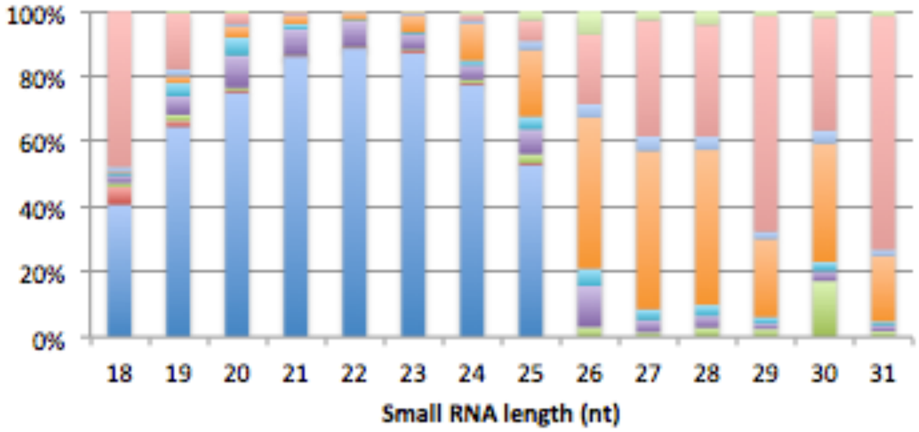

100%
80%
60%
40%
20%
0%
18
19
20
21
22
23
24
25
26
27
28
29
30
31
Small RNA length (nt)

Eye

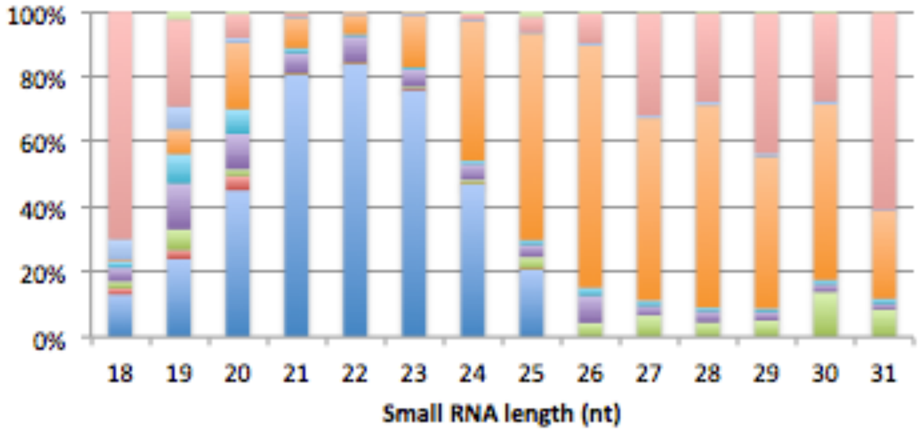

100%
80%
60%
40%
20%
0%
18
19
20
21
22
23
24
25
26
27
28
29
30
31
Small RNA length (nt)

Brain

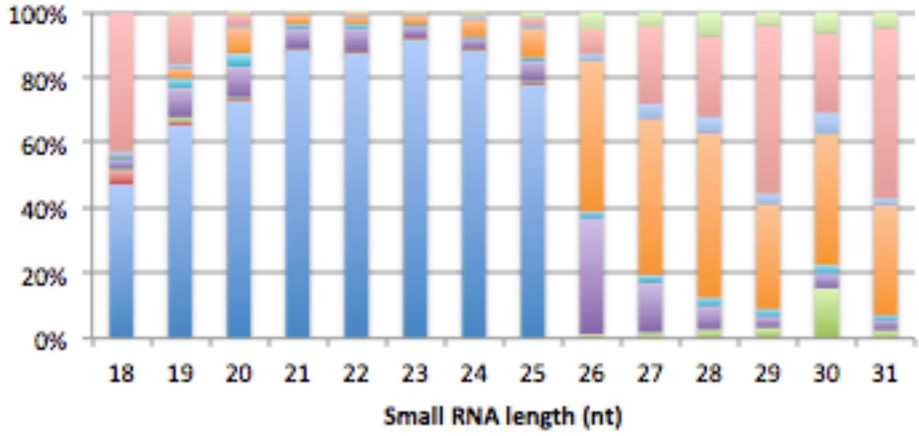

Intestine

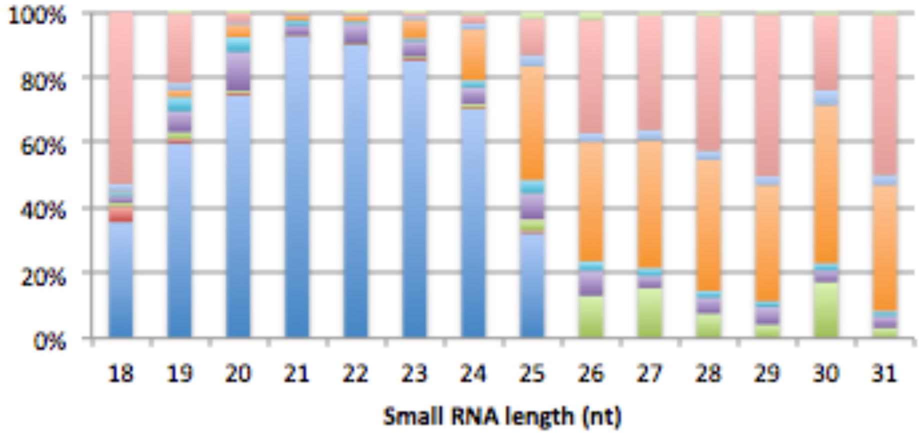

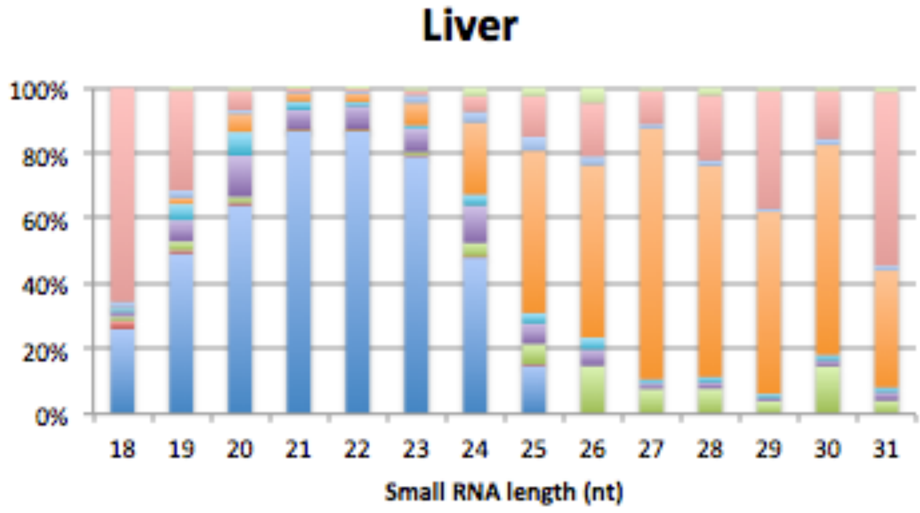
Liver
100%
80%
60%
40%
20%
0%
18
19
20
21
22
23
24
25
26
27
28
29
30
31
Small RNA length (nt)

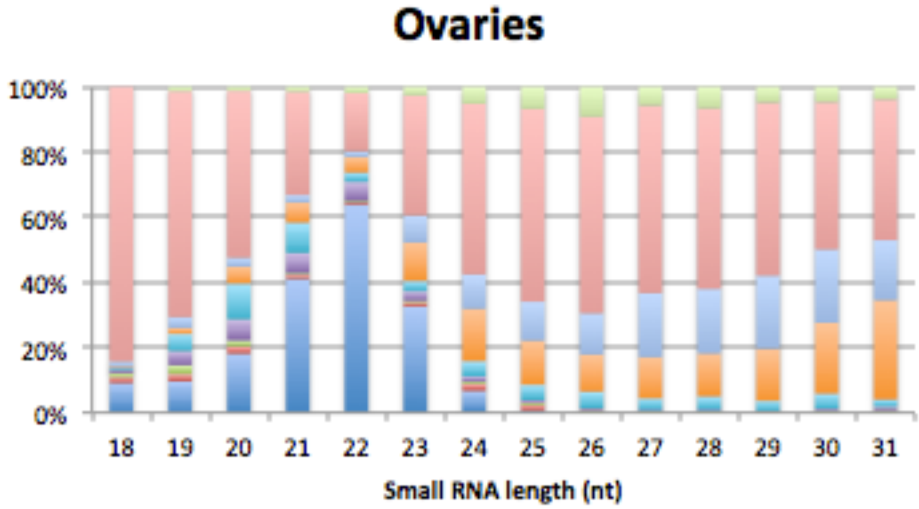
Ovaries
100%
80%
60%
40%
20%
0%
18
19
20
21
22
23
24
25
26
27
28
29
30
31
Small RNA length (nt)

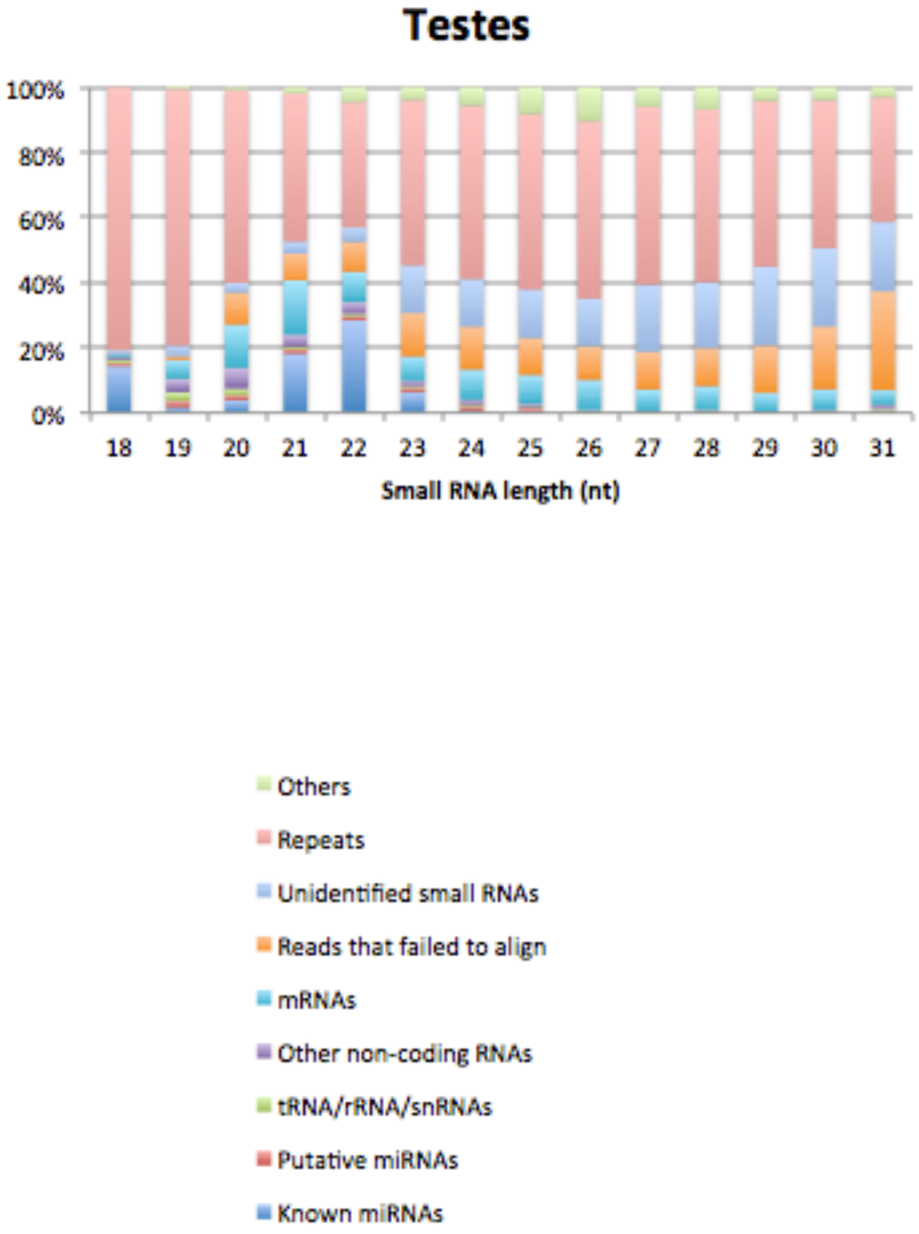
Testes
100%
80%
60%
40%
20%
0%
18
19
20
21
22
23
24
25
26
27
28
29
30
31
Small RNA length (nt)
Others
Repeats
Unidentified small RNAs
Reads that failed to align
mRNAs
Other non-coding RNAs
tRNA/rRNA/snRNAs
Putative miRNAs
Known miRNAs
