# Supplementary figures and images for: Deep sequencing, profiling and detailed annotation of microRNAs in Takifugu rubripes
Source: BMC Genomics. 2015 Jun 16;16(1):457. doi: 10.1186/s12864-015-1622-1 (PMC4469249; doi:10.1186/s12864-015-1622-1)

Additional file 8 : Figure S5

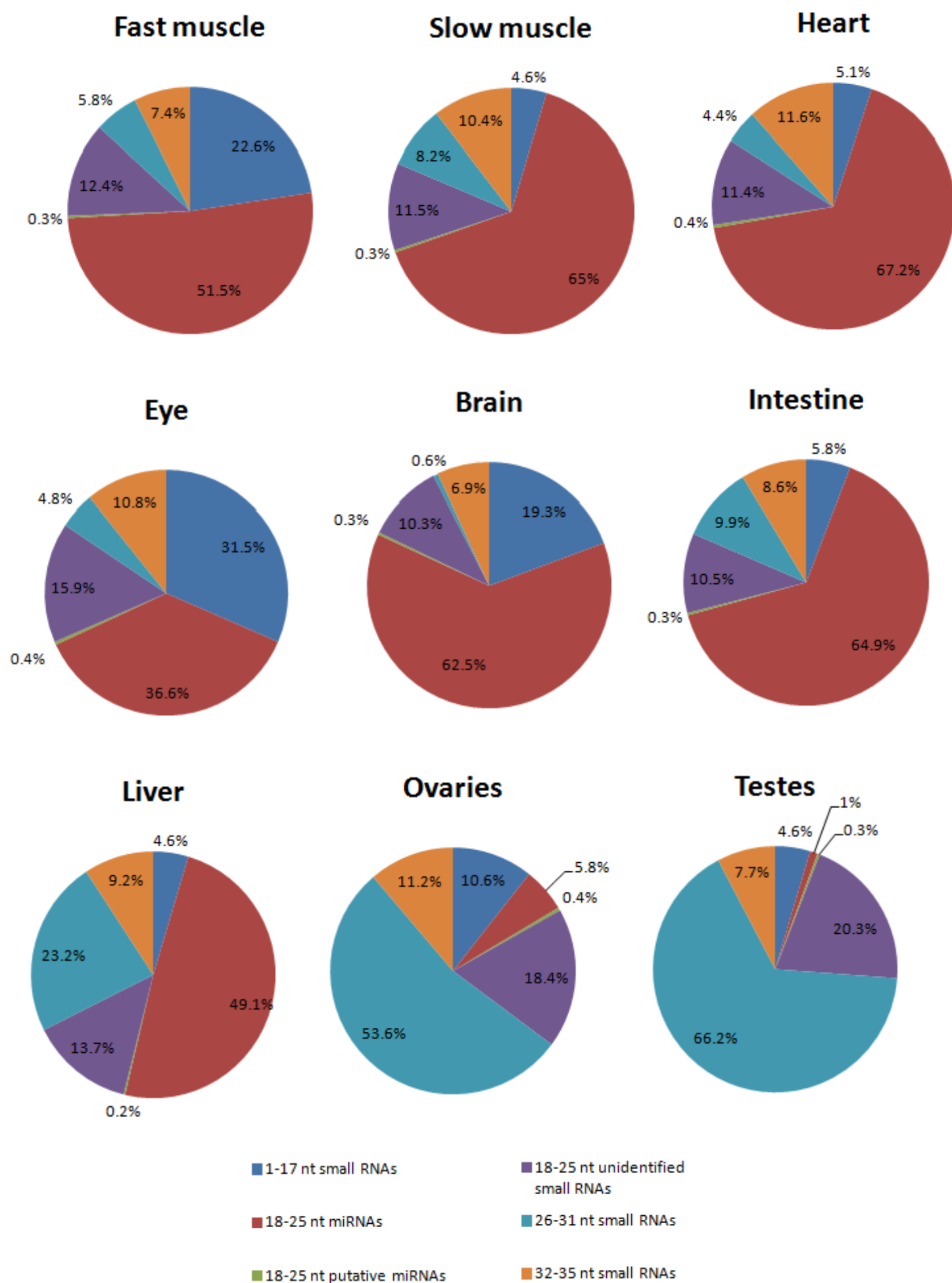

Supplement: Additional file 8: Figure S5. — miRNA proportions as percentages of 1–35 nt small RNAs. Proportions of 1–17 nt small RNAs, 18–25 nt miRNAs, 18–25 nt putative miRNAs, 18–25 nt unidentified small RNAs, 26–31 nt small RNAs, and 32–35 nt small RNAs are shown as percentages of total 1–35 nt small RNAs. [file 12864_2015_1622_MOESM8_ESM.pdf]
