# Supplementary material for: Deep sequencing, profiling and detailed annotation of microRNAs in Takifugu rubripes
Source: BMC Genomics. 2015 Jun 16;16(1):457. doi: 10.1186/s12864-015-1622-1 (PMC4469249; doi:10.1186/s12864-015-1622-1)

Additional file 10 : Figure S6

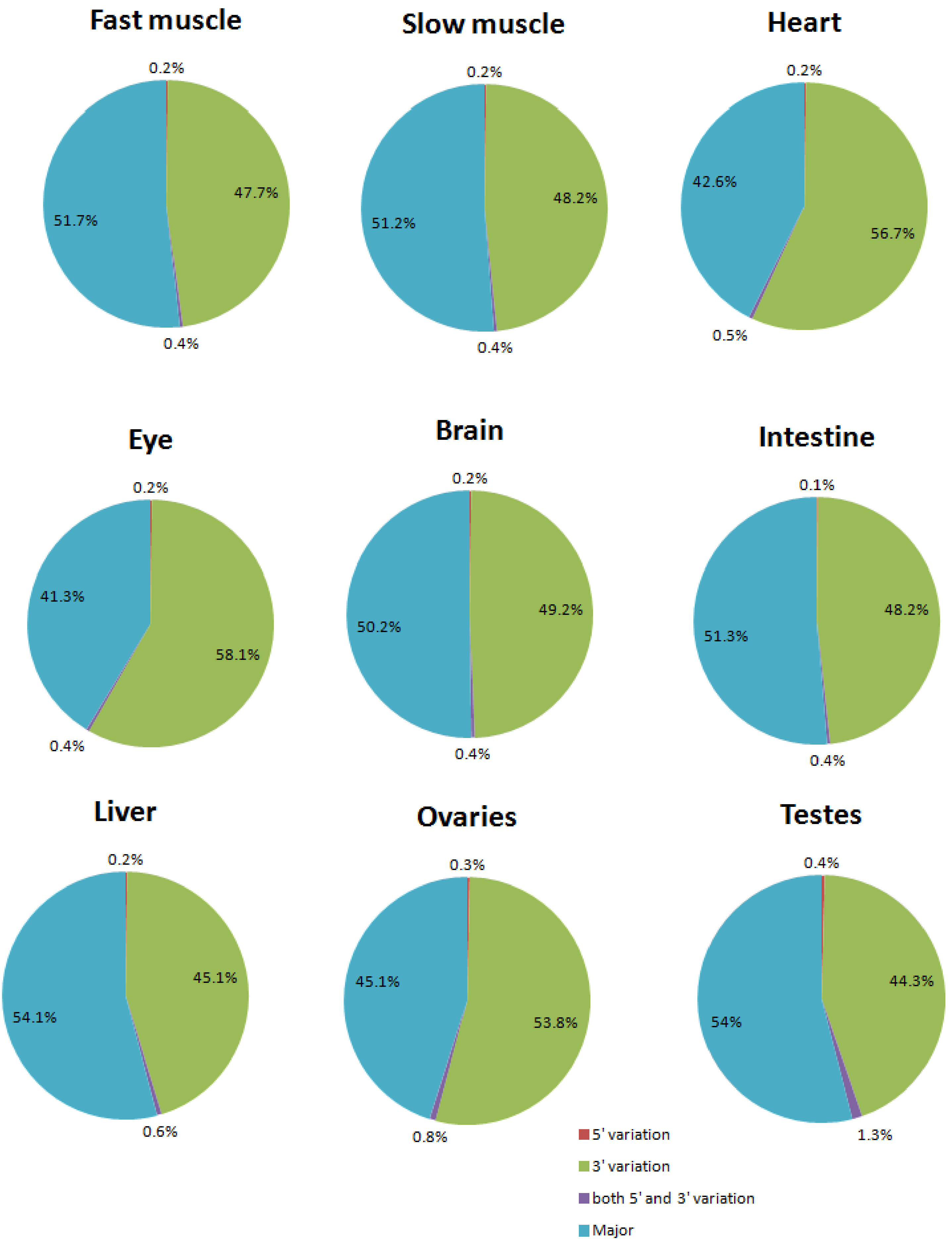

Supplement: Additional file 10: Figure S6. — Proportions of isomiR variation types as percentages of total fru-miR-145-5p in each tissue. Proportions of the isomiRs that exhibited nucleotide variation only in the 3′ region, the isomiRs that exhibited variation at both the 3′ and 5′ ends, the isomiRs that exhibited only 5′ variation, and the major sequence of the isomiRs are shown as percentages of total fru-miR-145-5p. [file 12864_2015_1622_MOESM10_ESM.pdf]
