# Supplementary material for: Deep sequencing, profiling and detailed annotation of microRNAs in Takifugu rubripes
Source: BMC Genomics. 2015 Jun 16;16(1):457. doi: 10.1186/s12864-015-1622-1 (PMC4469249; doi:10.1186/s12864-015-1622-1)
Supplement: Additional file 11: Figure S7. — IsomiRs of fru-miR-462-5p. The diagrams represent repertoires of fru-miR-462-5p isomiRs mapped to their miRNA precursors in each tissue. [file 12864_2015_1622_MOESM11_ESM.pdf]

Additional file 11 : Figure S7

Fast muscle

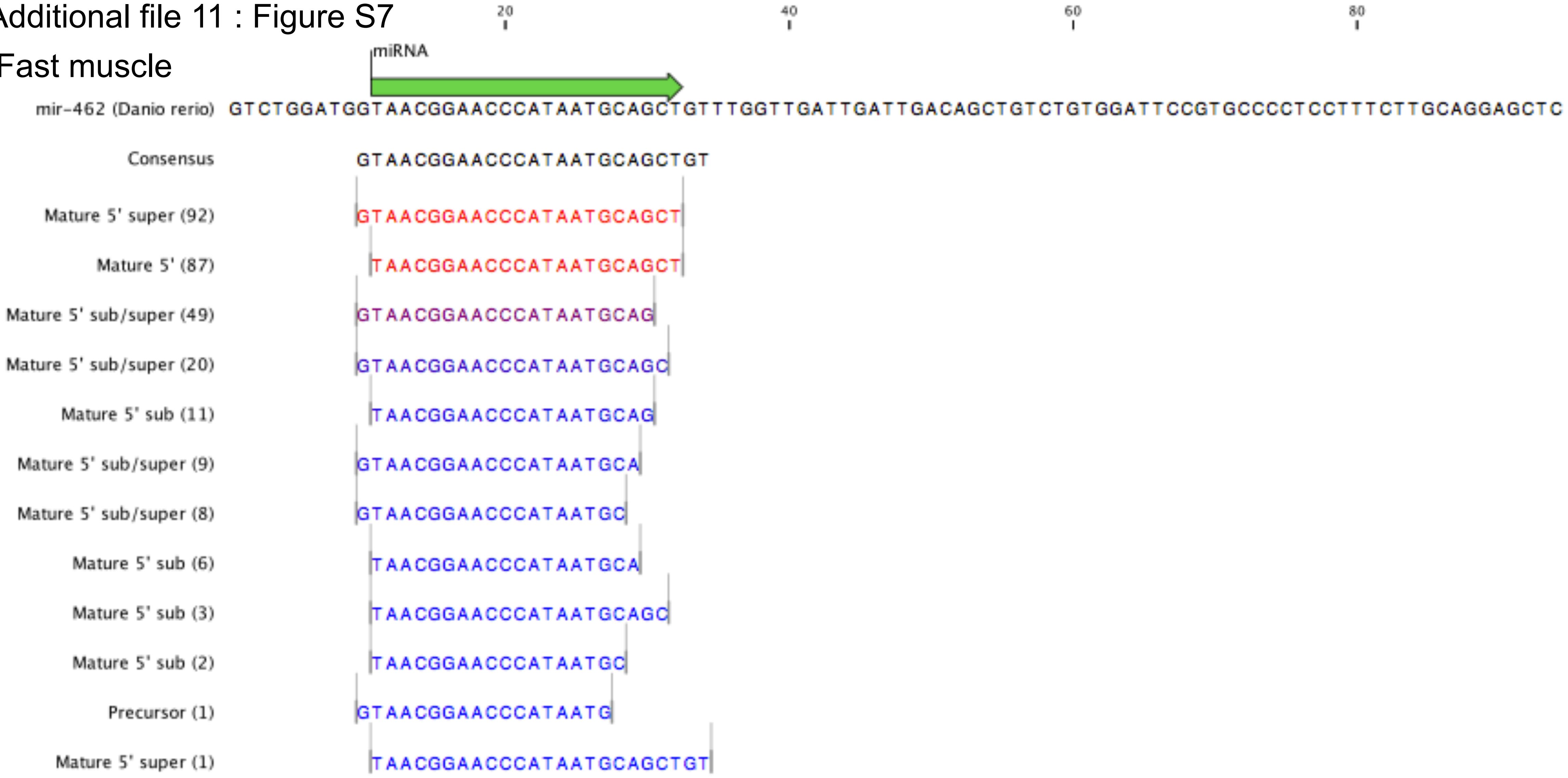

Slow muscle

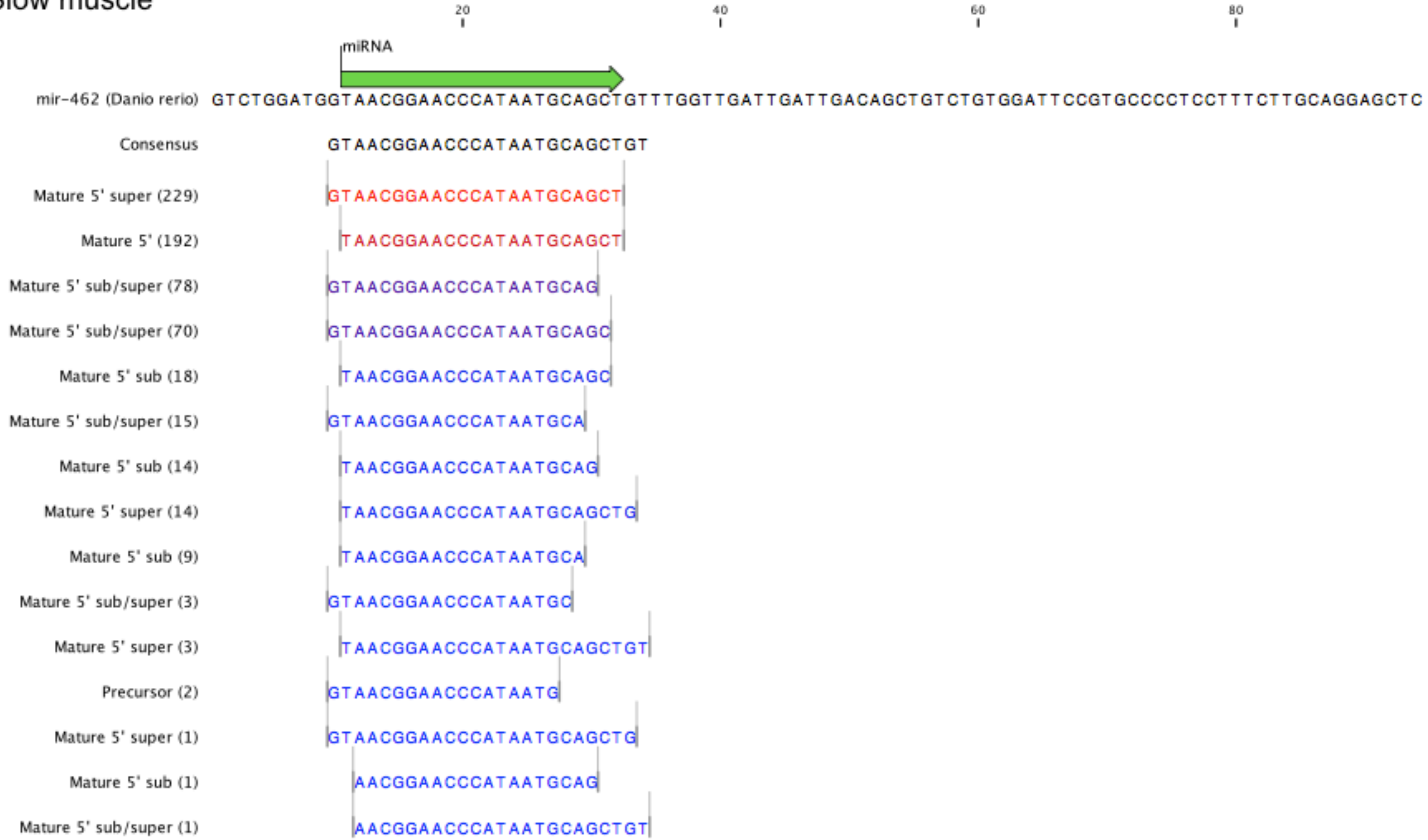
20
40
60
80
miRNA
mir-462 (Danio rerio) GTCTGGATGGTAACGGAACCCATAATGCAGCTGTTTGGTTGATTGATTGACAGCTGTCTGTGGATTCCGTGCCCCTCCTTTCTTGCAGGAGCTC
Consensus GTAACGGAACCCATAATGCAGCTGT
Mature 5' super (229) GTAACGGAACCCATAATGCAGCT
Mature 5' (192) TAACGGAACCCATAATGCAGCT
Mature 5' sub/super (78) GTAACGGAACCCATAATGCAG
Mature 5' sub/super (70) GTAACGGAACCCATAATGCAGC
Mature 5' sub (18) TAACGGAACCCATAATGCAGC
Mature 5' sub/super (15) GTAACGGAACCCATAATGCA
Mature 5' sub (14) TAACGGAACCCATAATGCAG
Mature 5' super (14) TAACGGAACCCATAATGCAGCTG
Mature 5' sub (9) TAACGGAACCCATAATGCA
Mature 5' sub/super (3) GTAACGGAACCCATAATGC
Mature 5' super (3) TAACGGAACCCATAATGCAGCTGT
Precursor (2) GTAACGGAACCCATAATG
Mature 5' super (1) GTAACGGAACCCATAATGCAGCTG
Mature 5' sub (1) AACGGAACCCATAATGCAG
Mature 5' sub/super (1) AACGGAACCCATAATGCAGCTGT

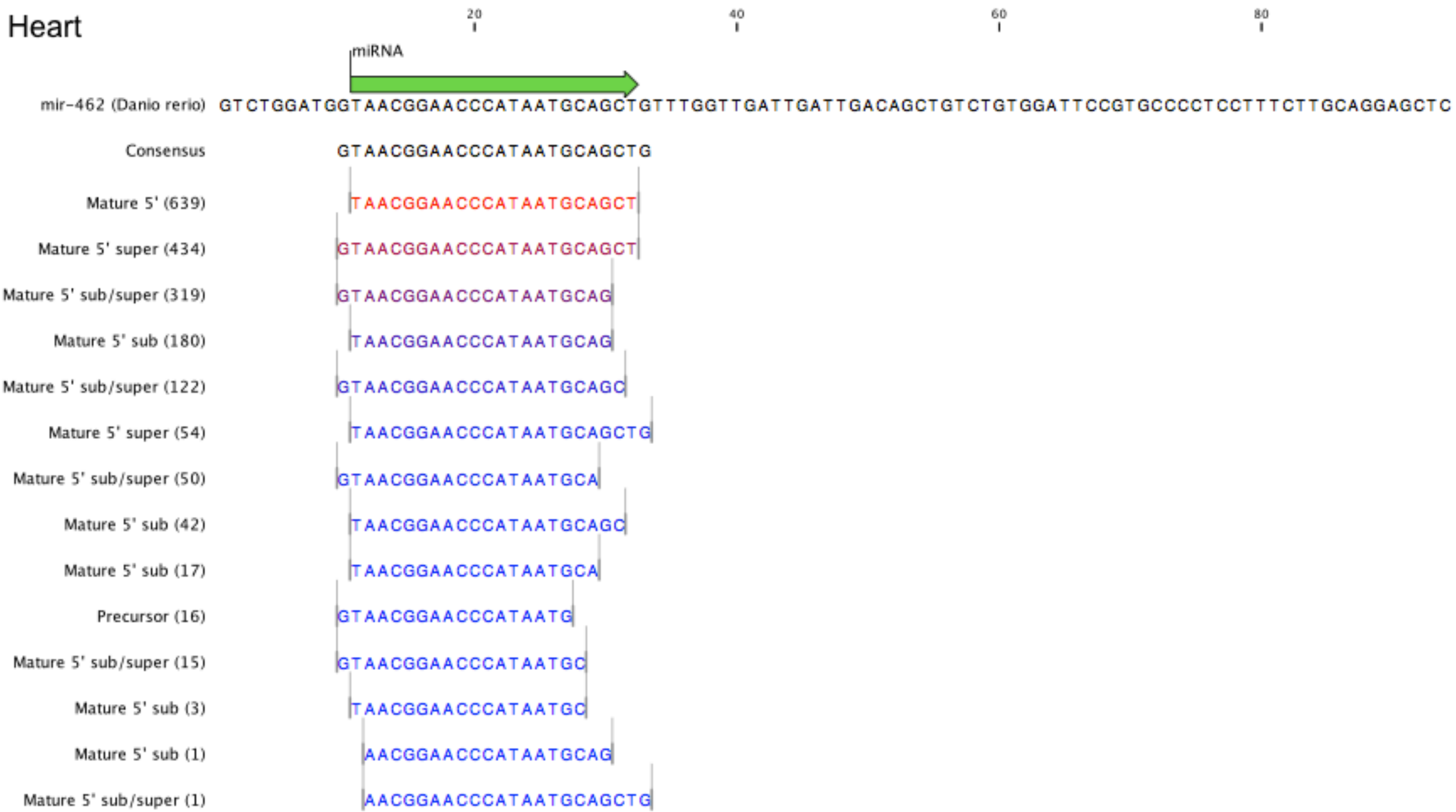
Heart
20
40
60
80
miRNA
mir-462 (Danio rerio) GTCTGGATGGTAACGGAACCCATAATGCAGCTGTTTGGTTGATTGATTGACAGCTGTCTGTGGATTCCGTGCCCCTCCTTTCTTGCAGGAGCTC
Consensus GTAACGGAACCCATAATGCAGCTG
Mature 5' (639) TAACGGAACCCATAATGCAGCT
Mature 5' super (434) GTAACGGAACCCATAATGCAGCT
Mature 5' sub/super (319) GTAACGGAACCCATAATGCAG
Mature 5' sub (180) TAACGGAACCCATAATGCAG
Mature 5' sub/super (122) GTAACGGAACCCATAATGCAGC
Mature 5' super (54) TAACGGAACCCATAATGCAGCTG
Mature 5' sub/super (50) GTAACGGAACCCATAATGCA
Mature 5' sub (42) TAACGGAACCCATAATGCAGC
Mature 5' sub (17) TAACGGAACCCATAATGCA
Precursor (16) GTAACGGAACCCATAATG
Mature 5' sub/super (15) GTAACGGAACCCATAATGC
Mature 5' sub (3) TAACGGAACCCATAATGC
Mature 5' sub (1) AACGGAACCCATAATGCAG
Mature 5' sub/super (1) AACGGAACCCATAATGCAGCTG

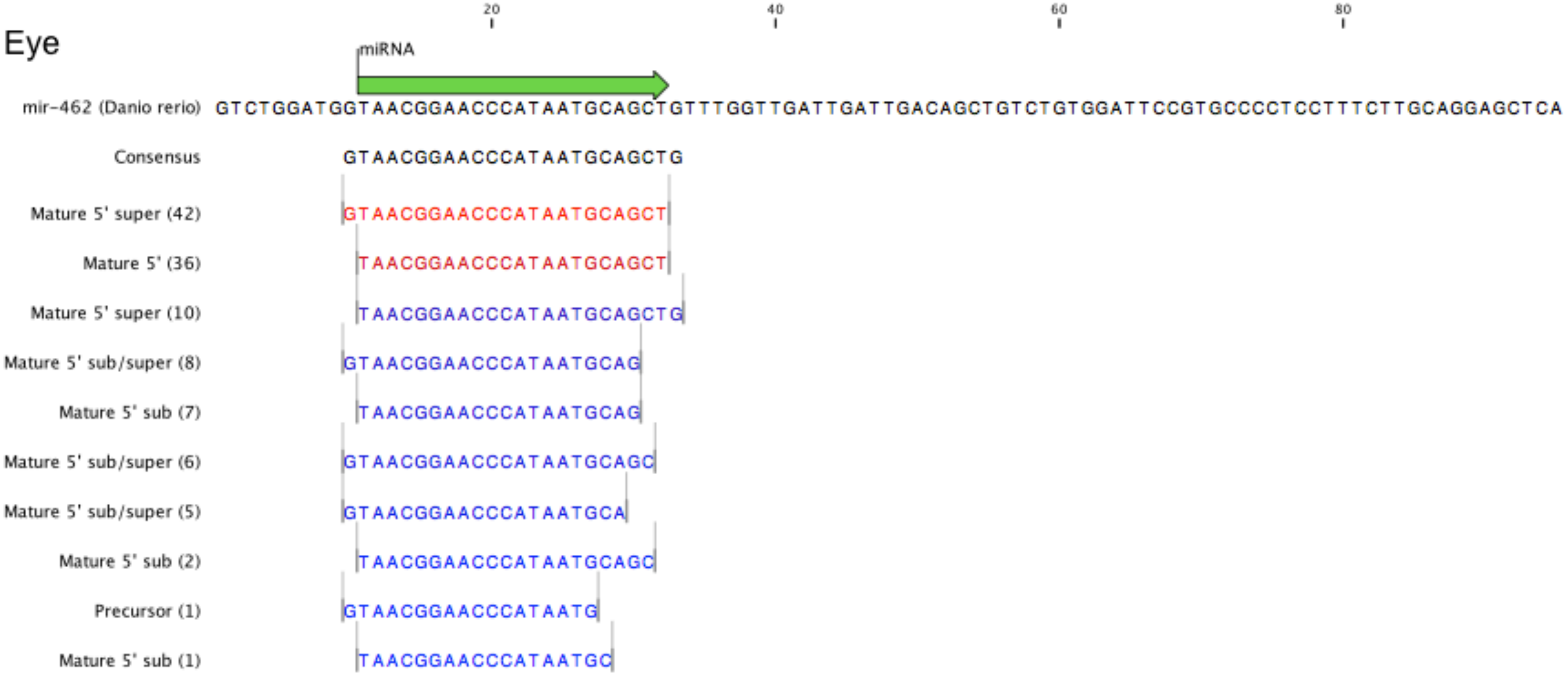
Eye
20
40
60
80
miRNA
mir-462 (Danio rerio) GTCTGGATGGTAACGGAACCCATAATGCAGCTGTTTGGTTGATTGATTGACAGCTGTCTGTGGATTCCGTGCCCCTCCTTTCTTGCAGGAGCTCA
Consensus GTAACGGAACCCATAATGCAGCTG
Mature 5' super (42) GTAACGGAACCCATAATGCAGCT
Mature 5' (36) TAACGGAACCCATAATGCAGCT
Mature 5' super (10) TAACGGAACCCATAATGCAGCTG
Mature 5' sub/super (8) GTAACGGAACCCATAATGCAG
Mature 5' sub (7) TAACGGAACCCATAATGCAG
Mature 5' sub/super (6) GTAACGGAACCCATAATGCAGC
Mature 5' sub/super (5) GTAACGGAACCCATAATGCA
Mature 5' sub (2) TAACGGAACCCATAATGCAGC
Precursor (1) GTAACGGAACCCATAATG
Mature 5' sub (1) TAACGGAACCCATAATGC

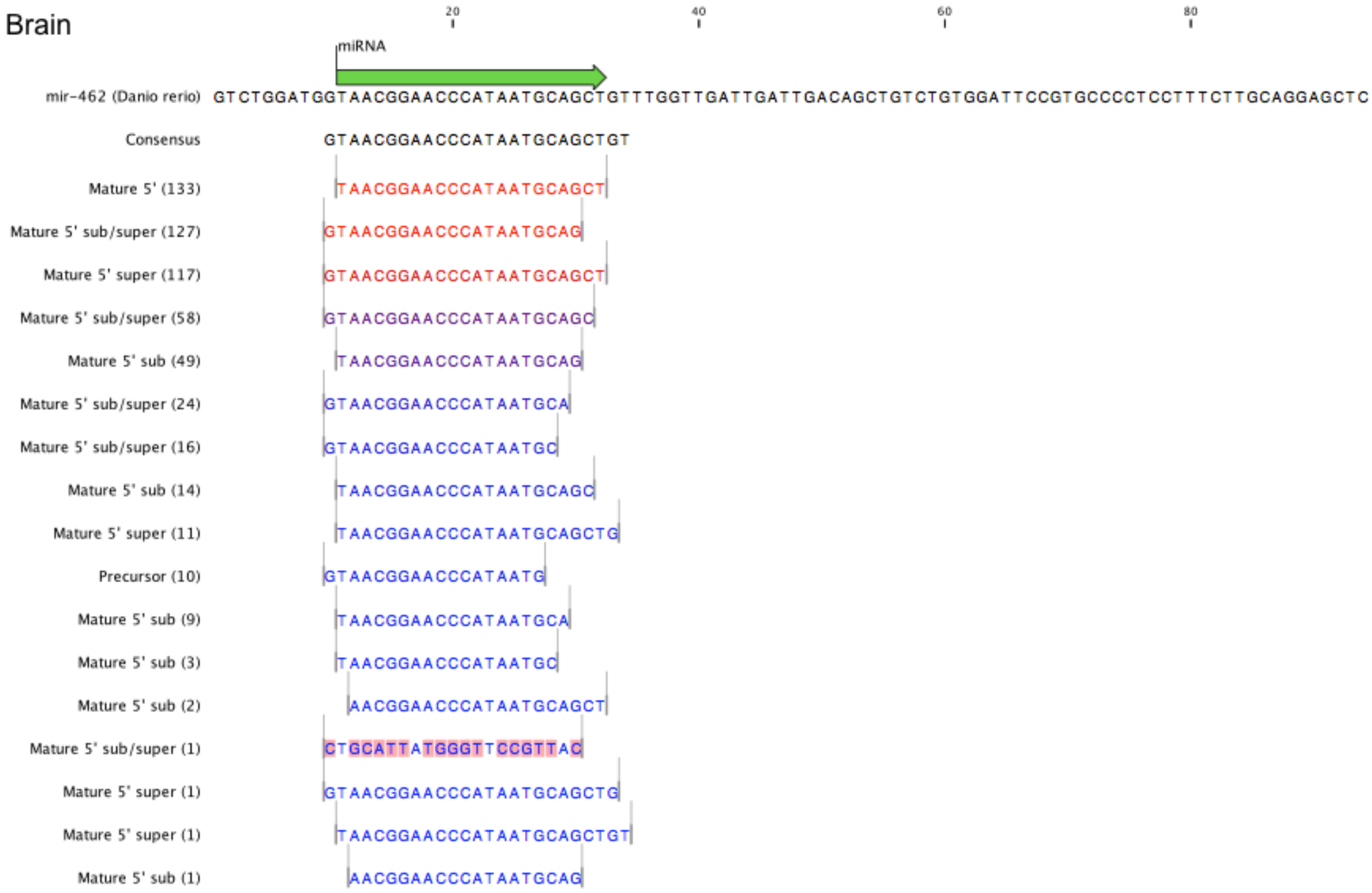
Brain
20
40
60
80
miRNA
mir-462 (Danio rerio) GTCTGGATGGTAACGGAACCCATAATGCAGCTGTTTGGTTGATTGATTGACAGCTGTCTGTGGATTCCGTGCCCCTCCTTTCTTGCAGGAGCTC
Consensus GTAACGGAACCCATAATGCAGCTGT
Mature 5' (133) TAACGGAACCCATAATGCAGCT
Mature 5' sub/super (127) GTAACGGAACCCATAATGCAG
Mature 5' super (117) GTAACGGAACCCATAATGCAGCT
Mature 5' sub/super (58) GTAACGGAACCCATAATGCAGC
Mature 5' sub (49) TAACGGAACCCATAATGCAG
Mature 5' sub/super (24) GTAACGGAACCCATAATGCA
Mature 5' sub/super (16) GTAACGGAACCCATAATGC
Mature 5' sub (14) TAACGGAACCCATAATGCAGC
Mature 5' super (11) TAACGGAACCCATAATGCAGCTG
Precursor (10) GTAACGGAACCCATAATG
Mature 5' sub (9) TAACGGAACCCATAATGCA
Mature 5' sub (3) TAACGGAACCCATAATGC
Mature 5' sub (2) AACGGAACCCATAATGCAGCT
Mature 5' sub/super (1) CTGCATTATGGGTTCCGTTAC
Mature 5' super (1) GTAACGGAACCCATAATGCAGCTG
Mature 5' super (1) TAACGGAACCCATAATGCAGCTGT
Mature 5' sub (1) AACGGAACCCATAATGCAG

Intestine

miRNA

mir-462 (Danio rerio) GTCTGGATGGTAACGGAACCCATAATGCAGCTGTTTGGTTGATTGATTGACAGCTGTCTGTGGATTCCGTGCCCCTCCTTTCTTGCAGGAGC

Consensus GTAACGGAACCCATAATGCAGCTGTT

Mature 5' super (6,161) GTAACGGAACCCATAATGCAGCT

Mature 5' (4,484) TAACGGAACCCATAATGCAGCT

Mature 5' sub/super (1,812) GTAACGGAACCCATAATGCAG

Mature 5' sub/super (1,568) GTAACGGAACCCATAATGCAGC

Mature 5' super (469) TAACGGAACCCATAATGCAGCTG

Mature 5' sub (393) TAACGGAACCCATAATGCAG

Mature 5' sub/super (289) GTAACGGAACCCATAATGCA

Mature 5' sub (240) TAACGGAACCCATAATGCAGC

Mature 5' sub/super (142) GTAACGGAACCCATAATGC

Precursor (119) GTAACGGAACCCATAATG

Mature 5' super (88) TAACGGAACCCATAATGCAGCTGT

Mature 5' sub (63) TAACGGAACCCATAATGCA

Mature 5' sub (33) AACGGAACCCATAATGCAGCT

Precursor (24) GGAACCCATAATGCAGCT

Mature 5' sub (20) TAACGGAACCCATAATGC

Mature 5' sub (9) AACGGAACCCATAATGCAG

Mature 5' super (5) TAACGGAACCCATAATGCAGCTGTT

Mature 5' sub/super (5) AACGGAACCCATAATGCAGCTGT

Mature 5' sub (5) ACGGAACCCATAATGCAGCT

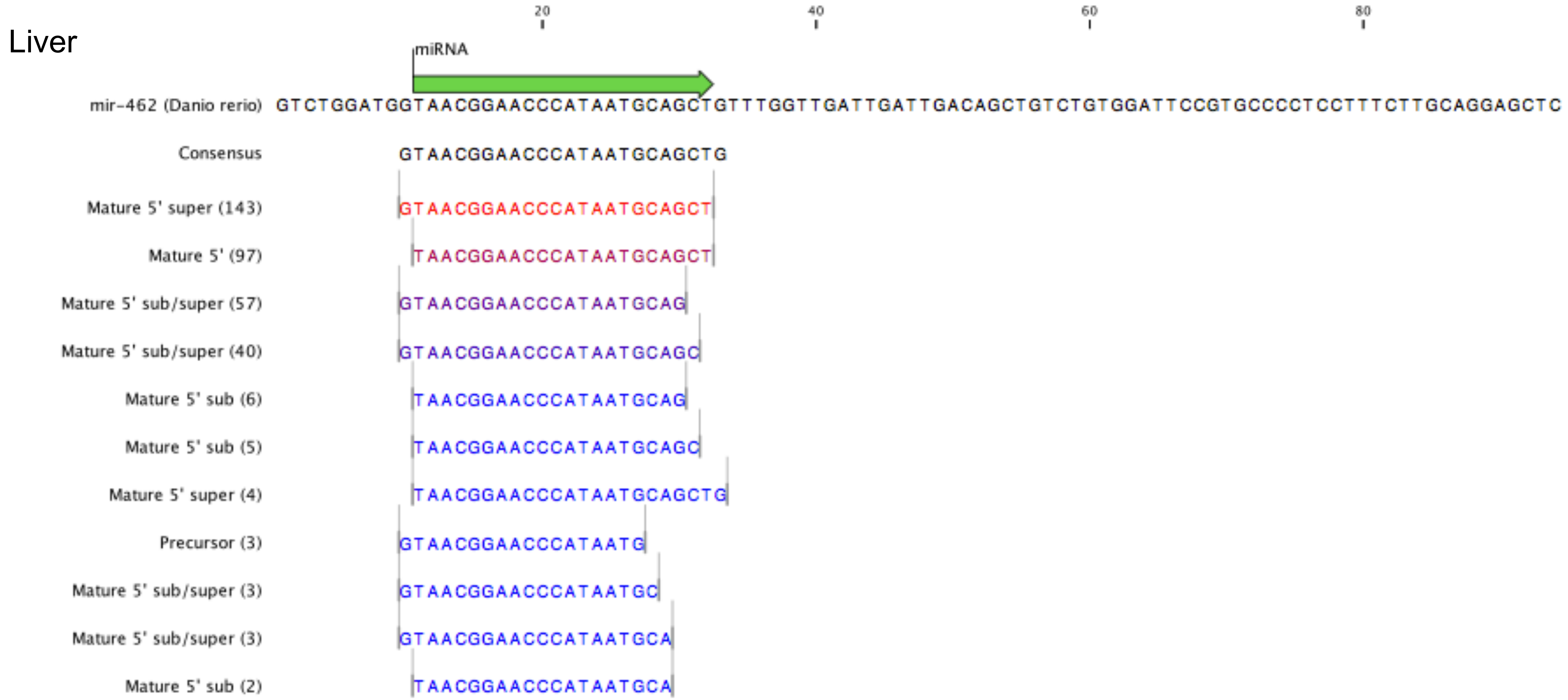
Liver
20
40
60
80
miRNA
mir-462 (Danio rerio) GTCTGGATGGTAACGGAACCCATAATGCAGCTGTTTGGTTGATTGATTGACAGCTGTCTGTGGATTCCGTGCCCCTCCTTTCTTGCAGGAGCTC
Consensus GTAACGGAACCCATAATGCAGCTG
Mature 5' super (143) GTAACGGAACCCATAATGCAGCT
Mature 5' (97) TAACGGAACCCATAATGCAGCT
Mature 5' sub/super (57) GTAACGGAACCCATAATGCAG
Mature 5' sub/super (40) GTAACGGAACCCATAATGCAGC
Mature 5' sub (6) TAACGGAACCCATAATGCAG
Mature 5' sub (5) TAACGGAACCCATAATGCAGC
Mature 5' super (4) TAACGGAACCCATAATGCAGCTG
Precursor (3) GTAACGGAACCCATAATG
Mature 5' sub/super (3) GTAACGGAACCCATAATGC
Mature 5' sub/super (3) GTAACGGAACCCATAATGCA
Mature 5' sub (2) TAACGGAACCCATAATGCA

Ovaries

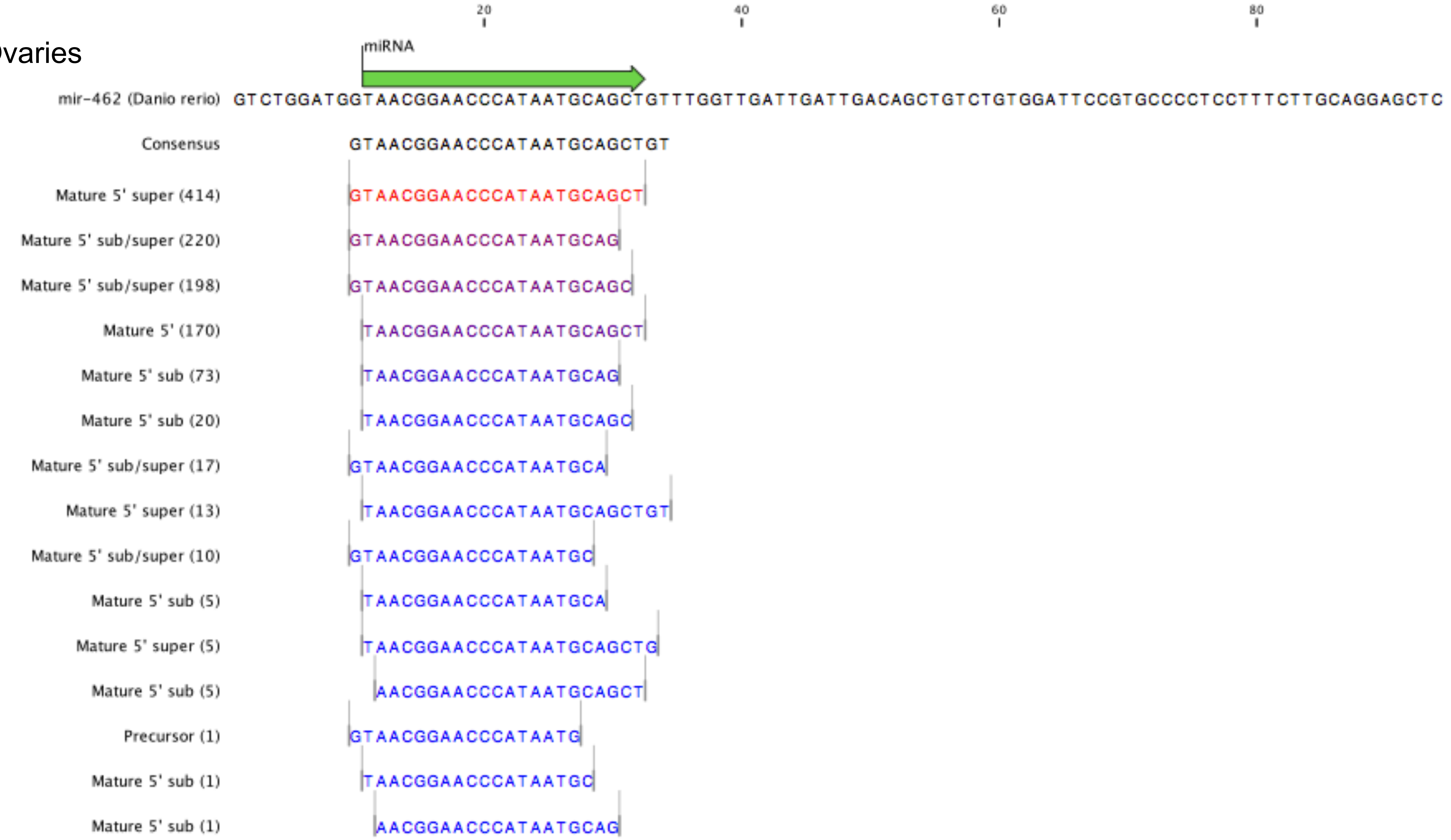
20
40
60
80
miRNA
mir-462 (Danio rerio) GTCTGGATGGTAACGGAACCCATAATGCAGCTGTTTGGTTGATTGATTGACAGCTGTCTGTGGATTCCGTGCCCCTCCTTTCTTGCAGGAGCTC
Consensus GTAACGGAACCCATAATGCAGCTGT
Mature 5' super (414) GTAACGGAACCCATAATGCAGCT
Mature 5' sub/super (220) GTAACGGAACCCATAATGCAG
Mature 5' sub/super (198) GTAACGGAACCCATAATGCAGC
Mature 5' (170) TAACGGAACCCATAATGCAGCT
Mature 5' sub (73) TAACGGAACCCATAATGCAG
Mature 5' sub (20) TAACGGAACCCATAATGCAGC
Mature 5' sub/super (17) GTAACGGAACCCATAATGCA
Mature 5' super (13) TAACGGAACCCATAATGCAGCTGT
Mature 5' sub/super (10) GTAACGGAACCCATAATGC
Mature 5' sub (5) TAACGGAACCCATAATGCA
Mature 5' super (5) TAACGGAACCCATAATGCAGCTG
Mature 5' sub (5) AACGGAACCCATAATGCAGCT
Precursor (1) GTAACGGAACCCATAATG
Mature 5' sub (1) TAACGGAACCCATAATGC
Mature 5' sub (1) AACGGAACCCATAATGCAG

Testes

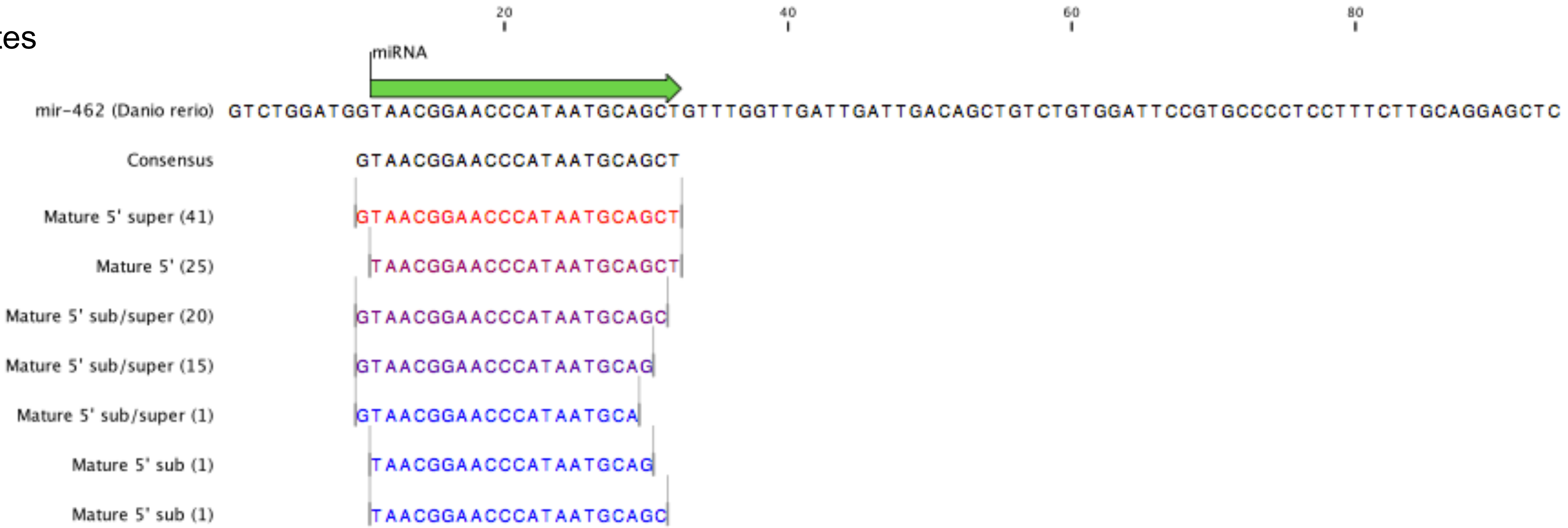
20
40
60
80
miRNA
mir-462 (Danio rerio)
GTCTGGATGGTAACGGAACCCATAATGCAGCTGTTTGGTTGATTGATTGACAGCTGTCTGTGGATTCCGTGCCCCTCCTTTCTTGCAGGAGCTC
Consensus
GTAACGGAACCCATAATGCAGCT
Mature 5' super (41)
GTAACGGAACCCATAATGCAGCT
Mature 5' (25)
TAACGGAACCCATAATGCAGCT
Mature 5' sub/super (20)
GTAACGGAACCCATAATGCAGC
Mature 5' sub/super (15)
GTAACGGAACCCATAATGCAG
Mature 5' sub/super (1)
GTAACGGAACCCATAATGCA
Mature 5' sub (1)
TAACGGAACCCATAATGCAG
Mature 5' sub (1)
TAACGGAACCCATAATGCAGC
