# Supplementary material for: Deep sequencing, profiling and detailed annotation of microRNAs in Takifugu rubripes
Source: BMC Genomics. 2015 Jun 16;16(1):457. doi: 10.1186/s12864-015-1622-1 (PMC4469249; doi:10.1186/s12864-015-1622-1)
Supplement: Additional file 12: Figure S8. — IsomiRs of fru-miR-101b-3p. The diagrams represent repertoires of fru-miR-101b-3p isomiRs mapped to their miRNA precursors in each tissue. [file 12864_2015_1622_MOESM12_ESM.pdf]

Additional file 12 : Figure S8

Fast muscle

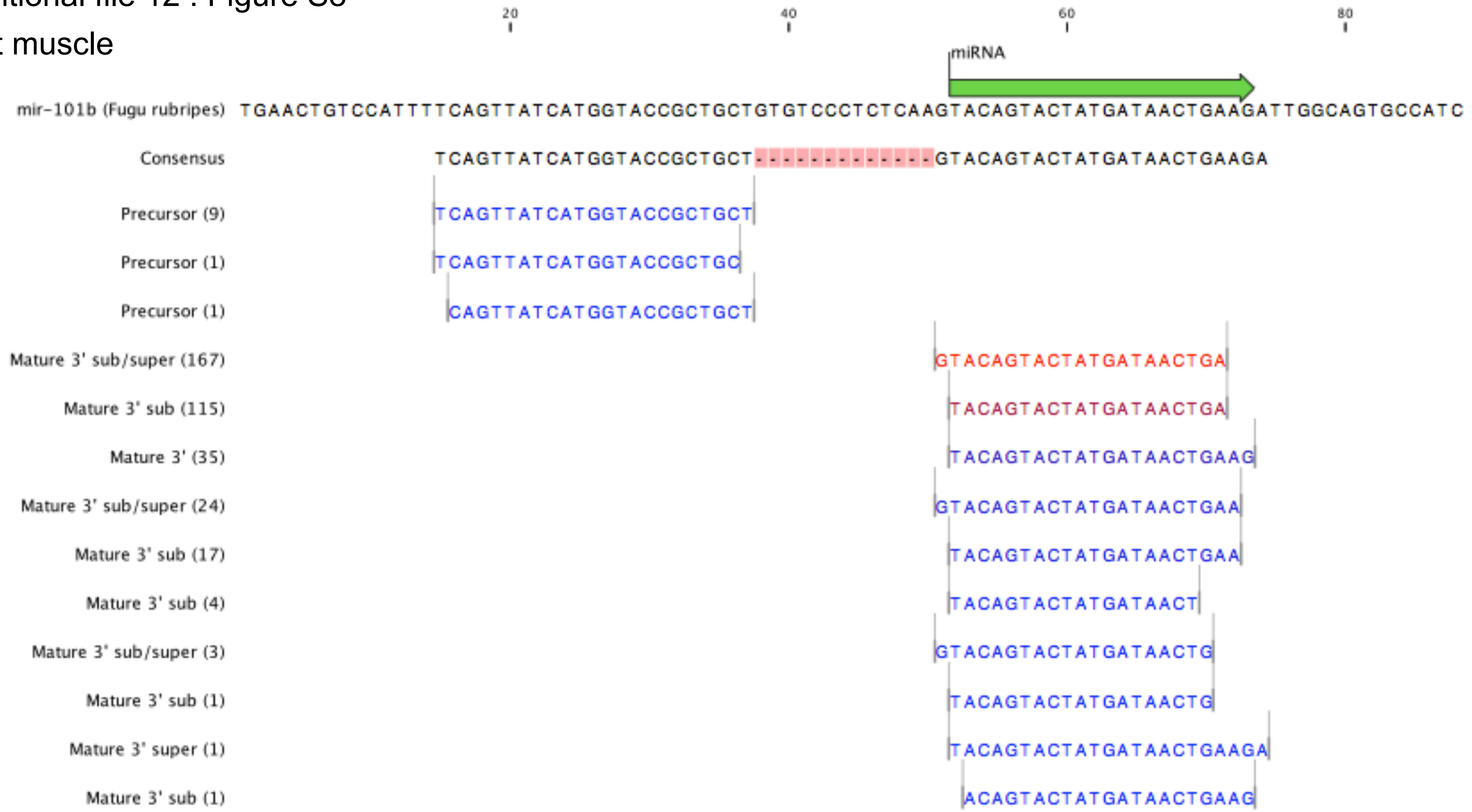

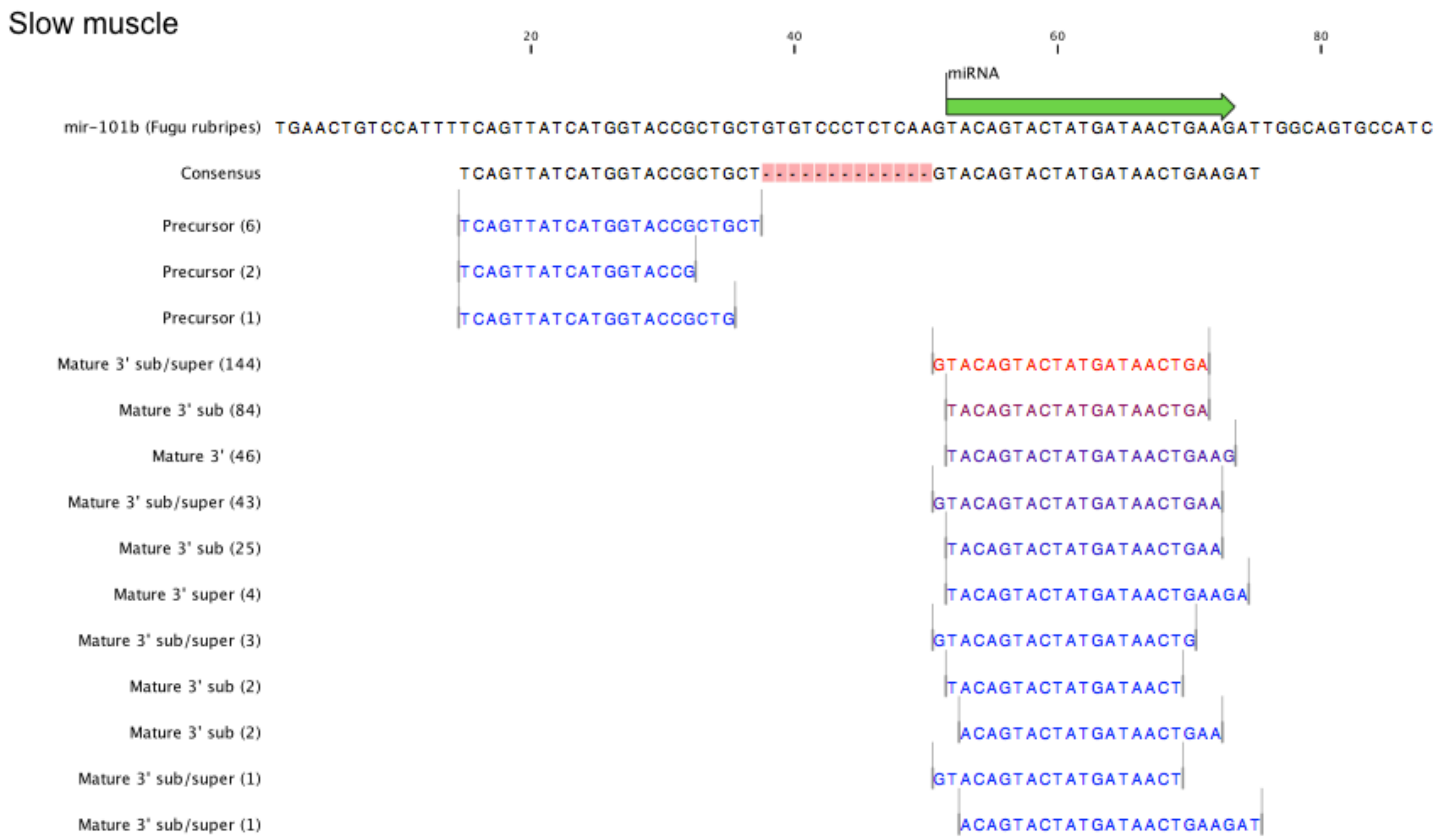
Slow muscle
20
40
60
80
miRNA
mir-101b (Fugu rubripes) TGAACTGTCCATTTTCAGTTATCATGGTACCGCTGCTGTGTCCCTCTCAAGTACAGTACTATGATAACTGAAGATTGGCAGTGCCATC
Consensus TCAGTTATCATGGTACCGCTGCT-------------GTACAGTACTATGATAACTGAAGAT
Precursor (6) TCAGTTATCATGGTACCGCTGCT
Precursor (2) TCAGTTATCATGGTACCG
Precursor (1) TCAGTTATCATGGTACCGCTG
Mature 3' sub/super (144) GTACAGTACTATGATAACTGA
Mature 3' sub (84) TACAGTACTATGATAACTGA
Mature 3' (46) TACAGTACTATGATAACTGAAG
Mature 3' sub/super (43) GTACAGTACTATGATAACTGAA
Mature 3' sub (25) TACAGTACTATGATAACTGAA
Mature 3' super (4) TACAGTACTATGATAACTGAAGA
Mature 3' sub/super (3) GTACAGTACTATGATAACTG
Mature 3' sub (2) TACAGTACTATGATAACT
Mature 3' sub (2) ACAGTACTATGATAACTGAA
Mature 3' sub/super (1) GTACAGTACTATGATAACT
Mature 3' sub/super (1) ACAGTACTATGATAACTGAAGAT

Heart

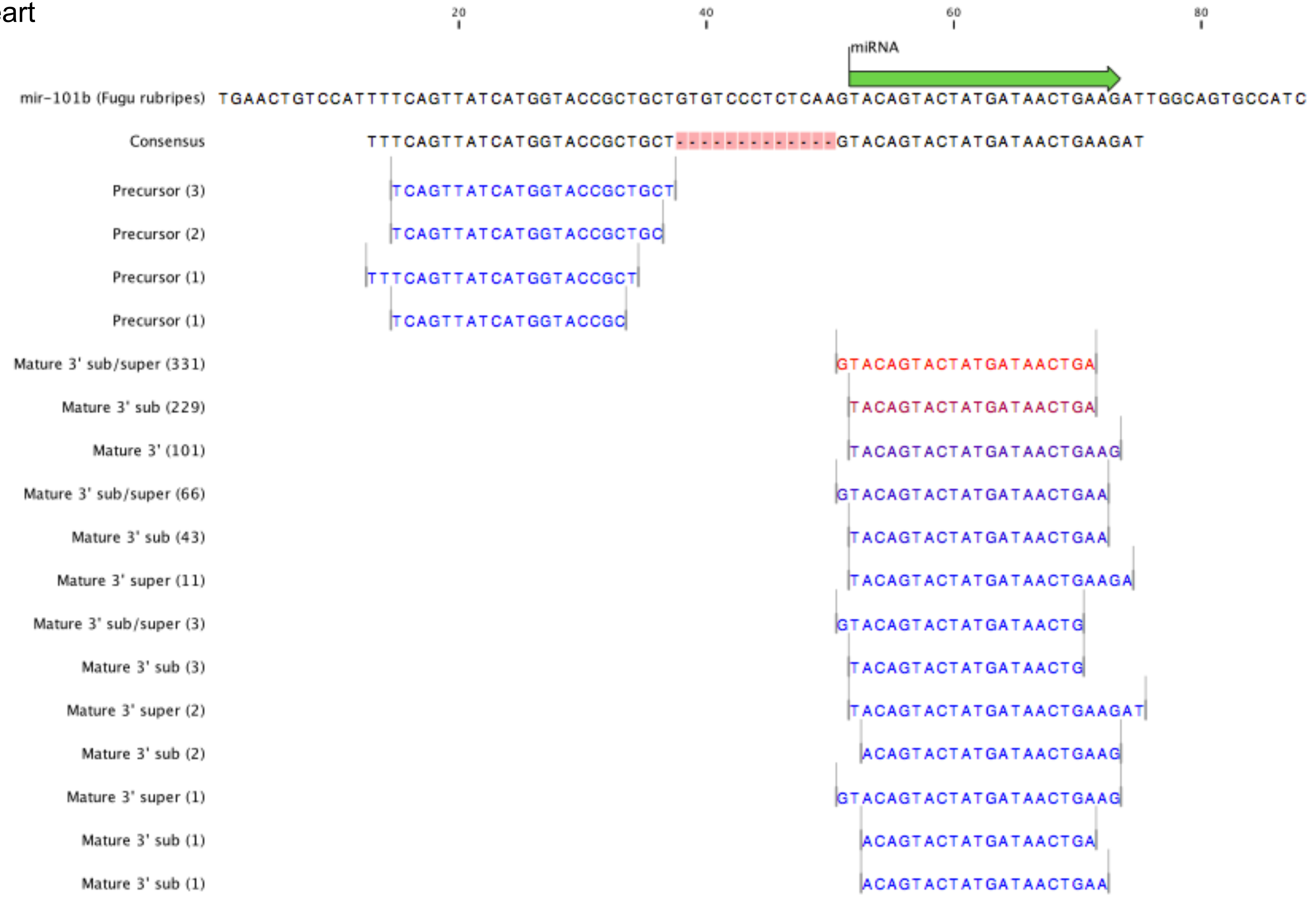

20
40
60
80
miRNA
mir-101b (Fugu rubripes) TGAACTGTCCATTTTCAGTTATCATGGTACCGCTGCTGTGTCCCTCTCAAGTACAGTACTATGATAACTGAAGATTGGCAGTGCCATC
Consensus TTTCAGTTATCATGGTACCGCTGCT-------------GTACAGTACTATGATAACTGAAGAT
Precursor (3) TCAGTTATCATGGTACCGCTGCT
Precursor (2) TCAGTTATCATGGTACCGCTGC
Precursor (1) TTTCAGTTATCATGGTACCGCT
Precursor (1) TCAGTTATCATGGTACCGC
Mature 3' sub/super (331) GTACAGTACTATGATAACTGA
Mature 3' sub (229) TACAGTACTATGATAACTGA
Mature 3' (101) TACAGTACTATGATAACTGAAG
Mature 3' sub/super (66) GTACAGTACTATGATAACTGAA
Mature 3' sub (43) TACAGTACTATGATAACTGAA
Mature 3' super (11) TACAGTACTATGATAACTGAAGA
Mature 3' sub/super (3) GTACAGTACTATGATAACTG
Mature 3' sub (3) TACAGTACTATGATAACTG
Mature 3' super (2) TACAGTACTATGATAACTGAAGAT
Mature 3' sub (2) ACAGTACTATGATAACTGAAG
Mature 3' super (1) GTACAGTACTATGATAACTGAAG
Mature 3' sub (1) ACAGTACTATGATAACTGA
Mature 3' sub (1) ACAGTACTATGATAACTGAA

Eye

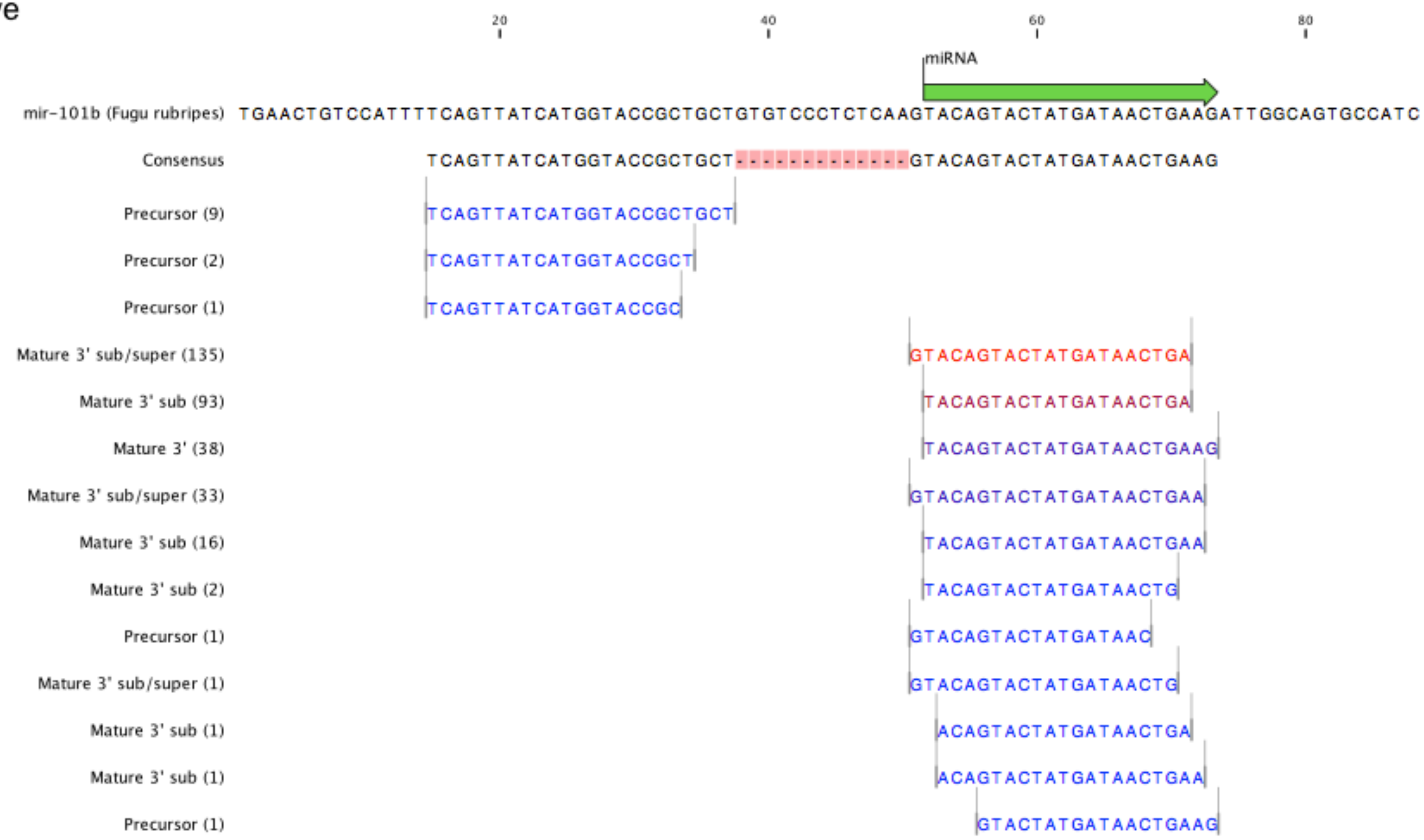

20
40
60
80
miRNA
mir-101b (Fugu rubripes) TGAACTGTCCATTTTCAGTTATCATGGTACCGCTGCTGTGTCCCTCTCAAGTACAGTACTATGATAACTGAAGATTGGCAGTGCCATC
Consensus TCAGTTATCATGGTACCGCTGCT-------------GTACAGTACTATGATAACTGAAG
Precursor (9) TCAGTTATCATGGTACCGCTGCT
Precursor (2) TCAGTTATCATGGTACCGCT
Precursor (1) TCAGTTATCATGGTACCGC
Mature 3' sub/super (135) GTACAGTACTATGATAACTGA
Mature 3' sub (93) TACAGTACTATGATAACTGA
Mature 3' (38) TACAGTACTATGATAACTGAAG
Mature 3' sub/super (33) GTACAGTACTATGATAACTGAA
Mature 3' sub (16) TACAGTACTATGATAACTGAA
Mature 3' sub (2) TACAGTACTATGATAACTG
Precursor (1) GTACAGTACTATGATAAC
Mature 3' sub/super (1) GTACAGTACTATGATAACTG
Mature 3' sub (1) ACAGTACTATGATAACTGA
Mature 3' sub (1) ACAGTACTATGATAACTGAA
Precursor (1) GTACTATGATAACTGAAG

Brain

	20 40 60 80
	miRNA
mir-101b (Fugu rubripes)	TGAACTGTCCATTTTCAGTTATCATGGTACCGCTGCTGTGTCCCTCTCAAGTACAGTACTATGATAACTGAAGATTGGCAGTGCCATC
Consensus	TCAGTTATCATGGTACCGCTGCT-------------GTACAGTACTATGATAACTGAAGA
Precursor (7)	TCAGTTATCATGGTACCGCTGCT
Precursor (4)	TCAGTTATCATGGTACCGCTGC
Precursor (2)	TCAGTTATCATGGTACCG
Precursor (2)	TCAGTTATCATGGTACCGCT
Precursor (1)	TCAGTTATCATGGTACCGCTG
Precursor (1)	CAGTTATCATGGTACCGCTGCT
Mature 3' sub (283)	TACAGTACTATGATAACTGA
Mature 3' sub/super (266)	GTACAGTACTATGATAACTGA
Mature 3' (85)	TACAGTACTATGATAACTGAAG
Mature 3' sub (66)	TACAGTACTATGATAACTGAA
Mature 3' sub/super (59)	GTACAGTACTATGATAACTGAA
Mature 3' super (11)	TACAGTACTATGATAACTGAAGA
Mature 3' sub (6)	TACAGTACTATGATAACTG
Mature 3' sub (5)	ACAGTACTATGATAACTGA
Precursor (3)	GTACAGTACTATGATAAC
Mature 3' sub/super (3)	GTACAGTACTATGATAACT
Mature 3' sub/super (3)	GTACAGTACTATGATAACTG
Mature 3' super (1)	GTACAGTACTATGATAACTGAAG
Mature 3' super (1)	GTACAGTACTATGATAACTGAAGA
Mature 3' sub (1)	TACAGTACTATGATAACT

Mature 3' sub/super (1)	ACAGTACTATGATAACTGAAGA
Precursor (1)	AGTACTATGATAACTGAAG
Precursor (1)	GTACTATGATAACTGAAGA

Intestine

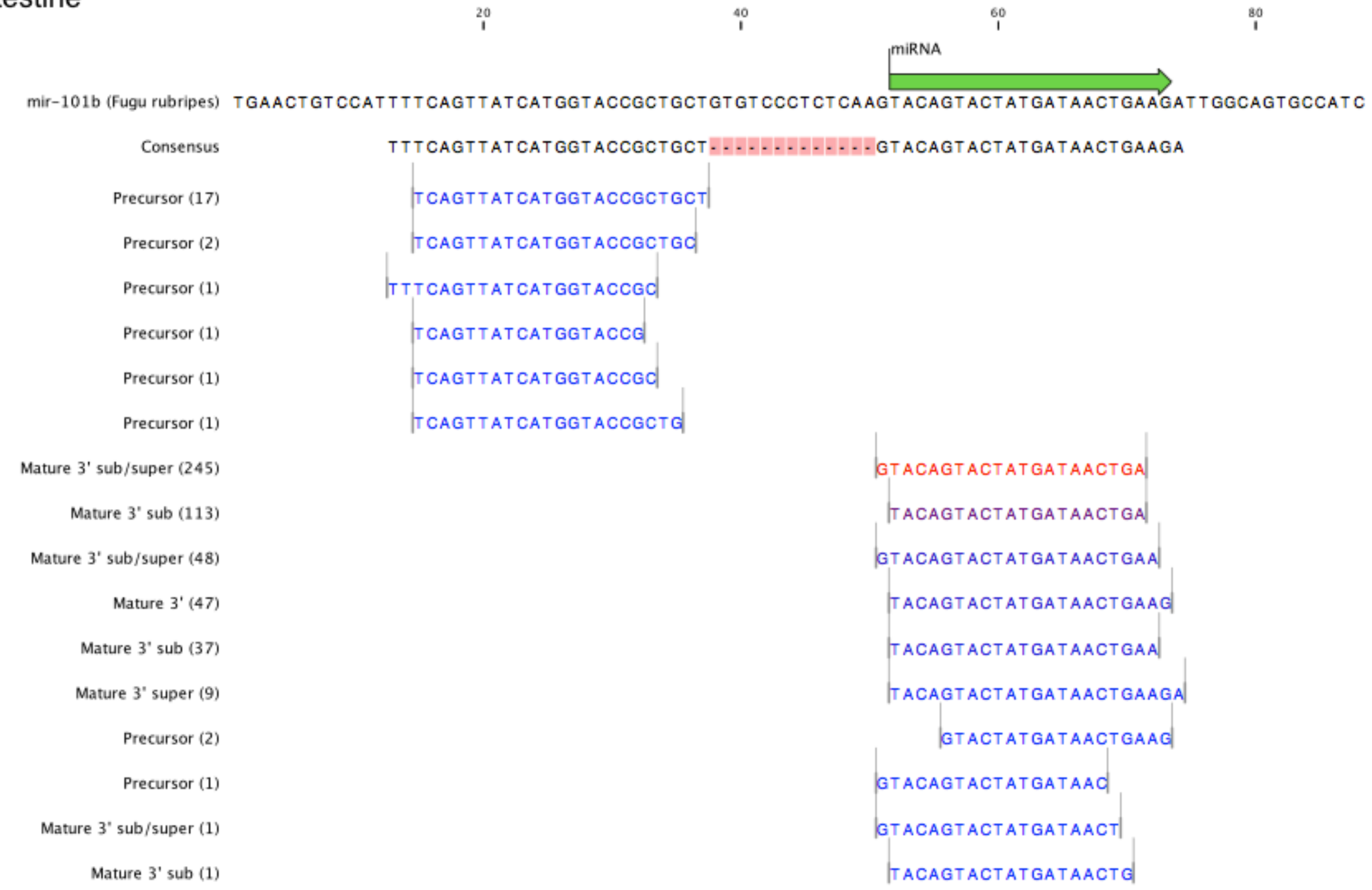

20
40
60
80
miRNA
mir-101b (Fugu rubripes) TGAACTGTCCATTTTCAGTTATCATGGTACCGCTGCTGTGTCCCTCTCAAGTACAGTACTATGATAACTGAAGATTGGCAGTGCCATC
Consensus TTTCAGTTATCATGGTACCGCTGCT-------------GTACAGTACTATGATAACTGAAGA
Precursor (17) TCAGTTATCATGGTACCGCTGCT
Precursor (2) TCAGTTATCATGGTACCGCTGC
Precursor (1) TTTCAGTTATCATGGTACCGC
Precursor (1) TCAGTTATCATGGTACCG
Precursor (1) TCAGTTATCATGGTACCGC
Precursor (1) TCAGTTATCATGGTACCGCTG
Mature 3' sub/super (245) GTACAGTACTATGATAACTGA
Mature 3' sub (113) TACAGTACTATGATAACTGA
Mature 3' sub/super (48) GTACAGTACTATGATAACTGAA
Mature 3' (47) TACAGTACTATGATAACTGAAG
Mature 3' sub (37) TACAGTACTATGATAACTGAA
Mature 3' super (9) TACAGTACTATGATAACTGAAGA
Precursor (2) GTACTATGATAACTGAAG
Precursor (1) GTACAGTACTATGATAAC
Mature 3' sub/super (1) GTACAGTACTATGATAACT
Mature 3' sub (1) TACAGTACTATGATAACTG

Liver

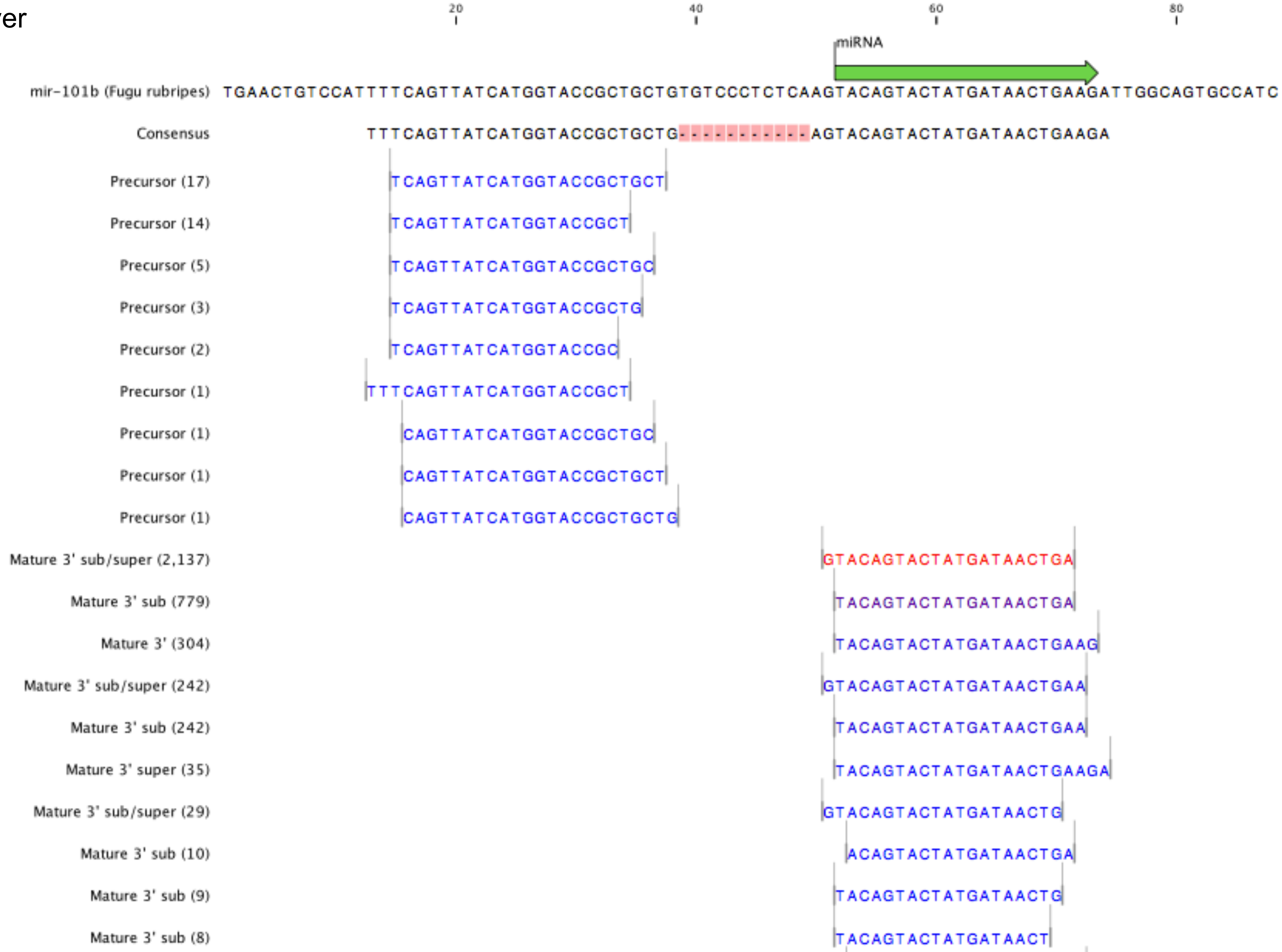
20
40
60
80
miRNA
mir-101b (Fugu rubripes) TGAACTGTCCATTTTCAGTTATCATGGTACCGCTGCTGTGTCCCTCTCAAGTACAGTACTATGATAACTGAAGATTGGCAGTGCCATC
Consensus TTTCAGTTATCATGGTACCGCTGCTG-----------AGTACAGTACTATGATAACTGAAGA
Precursor (17) TCAGTTATCATGGTACCGCTGCT
Precursor (14) TCAGTTATCATGGTACCGCT
Precursor (5) TCAGTTATCATGGTACCGCTGC
Precursor (3) TCAGTTATCATGGTACCGCTG
Precursor (2) TCAGTTATCATGGTACCGC
Precursor (1) TTTCAGTTATCATGGTACCGCT
Precursor (1) CAGTTATCATGGTACCGCTGC
Precursor (1) CAGTTATCATGGTACCGCTGCT
Precursor (1) CAGTTATCATGGTACCGCTGCTG
Mature 3' sub/super (2,137) GTACAGTACTATGATAACTGA
Mature 3' sub (779) TACAGTACTATGATAACTGA
Mature 3' (304) TACAGTACTATGATAACTGAAG
Mature 3' sub/super (242) GTACAGTACTATGATAACTGAA
Mature 3' sub (242) TACAGTACTATGATAACTGAA
Mature 3' super (35) TACAGTACTATGATAACTGAAGA
Mature 3' sub/super (29) GTACAGTACTATGATAACTG
Mature 3' sub (10) ACAGTACTATGATAACTGA
Mature 3' sub (9) TACAGTACTATGATAACTG
Mature 3' sub (8) TACAGTACTATGATAACT

Mature 3' sub (7)	ACAGTACTATGATAACTGAA
Mature 3' sub (7)	ACAGTACTATGATAACTGAAG
Precursor (5)	GTACAGTACTATGATAAC
Mature 3' sub/super (1)	AGTACAGTACTATGATAACTGA
Mature 3' sub/super (1)	GTACAGTACTATGATAACT
Mature 3' super (1)	GTACAGTACTATGATAACTGAAG
Mature 3' super (1)	GTACAGTACTATGATAACTGAAGA
Mature 3' sub (1)	ACAGTACTATGATAACTG

Ovaries

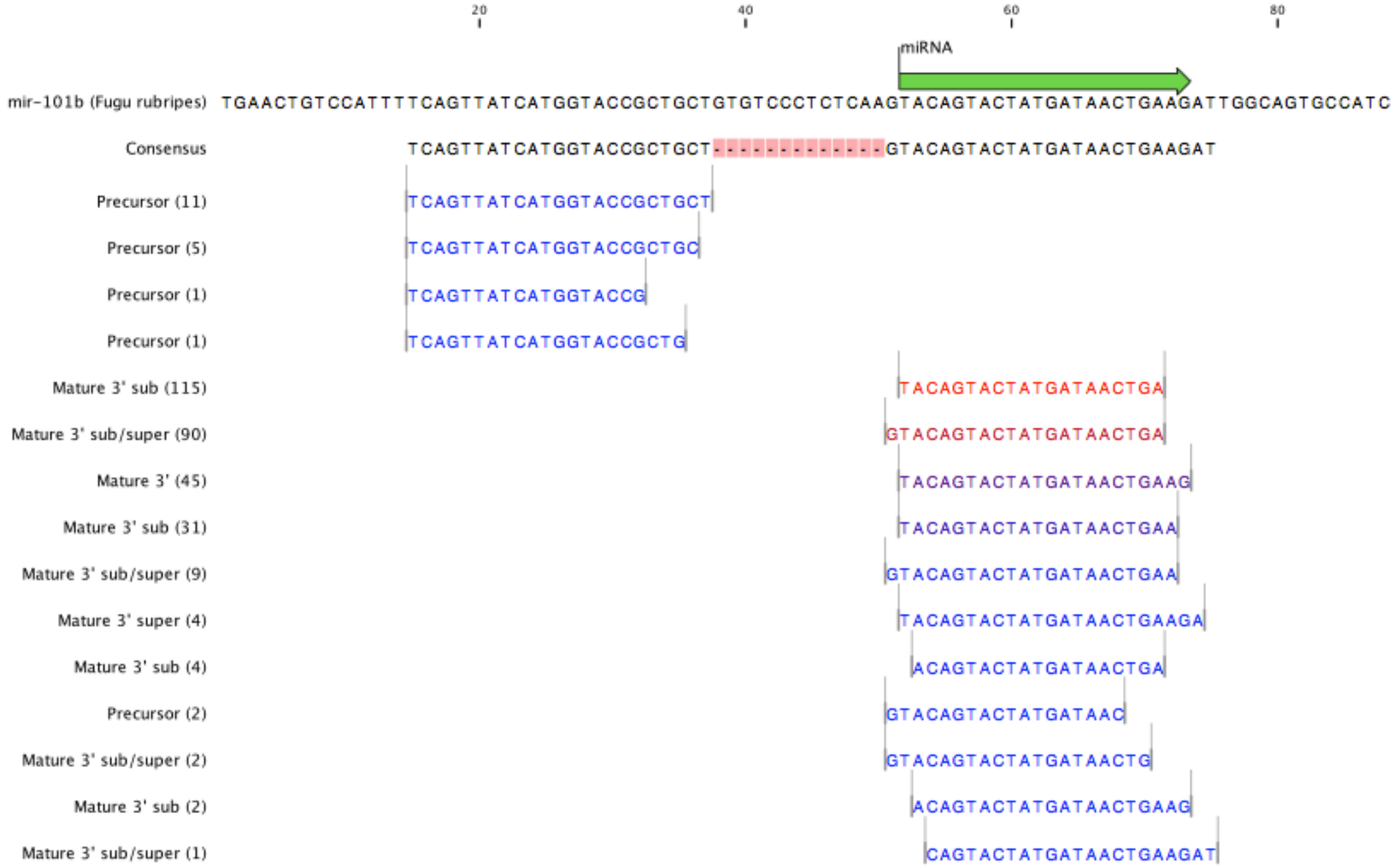
20
40
60
80
miRNA
mir-101b (Fugu rubripes) TGAACTGTCCATTTTCAGTTATCATGGTACCGCTGCTGTGTCCCTCTCAAGTACAGTACTATGATAACTGAAGATTGGCAGTGCCATC
Consensus TCAGTTATCATGGTACCGCTGCT-------------GTACAGTACTATGATAACTGAAGAT
Precursor (11) TCAGTTATCATGGTACCGCTGCT
Precursor (5) TCAGTTATCATGGTACCGCTGC
Precursor (1) TCAGTTATCATGGTACCG
Precursor (1) TCAGTTATCATGGTACCGCTG
Mature 3' sub (115) TACAGTACTATGATAACTGA
Mature 3' sub/super (90) GTACAGTACTATGATAACTGA
Mature 3' (45) TACAGTACTATGATAACTGAAG
Mature 3' sub (31) TACAGTACTATGATAACTGAA
Mature 3' sub/super (9) GTACAGTACTATGATAACTGAA
Mature 3' super (4) TACAGTACTATGATAACTGAAGA
Mature 3' sub (4) ACAGTACTATGATAACTGA
Precursor (2) GTACAGTACTATGATAAC
Mature 3' sub/super (2) GTACAGTACTATGATAACTG
Mature 3' sub (2) ACAGTACTATGATAACTGAAG
Mature 3' sub/super (1) CAGTACTATGATAACTGAAGAT

Testes

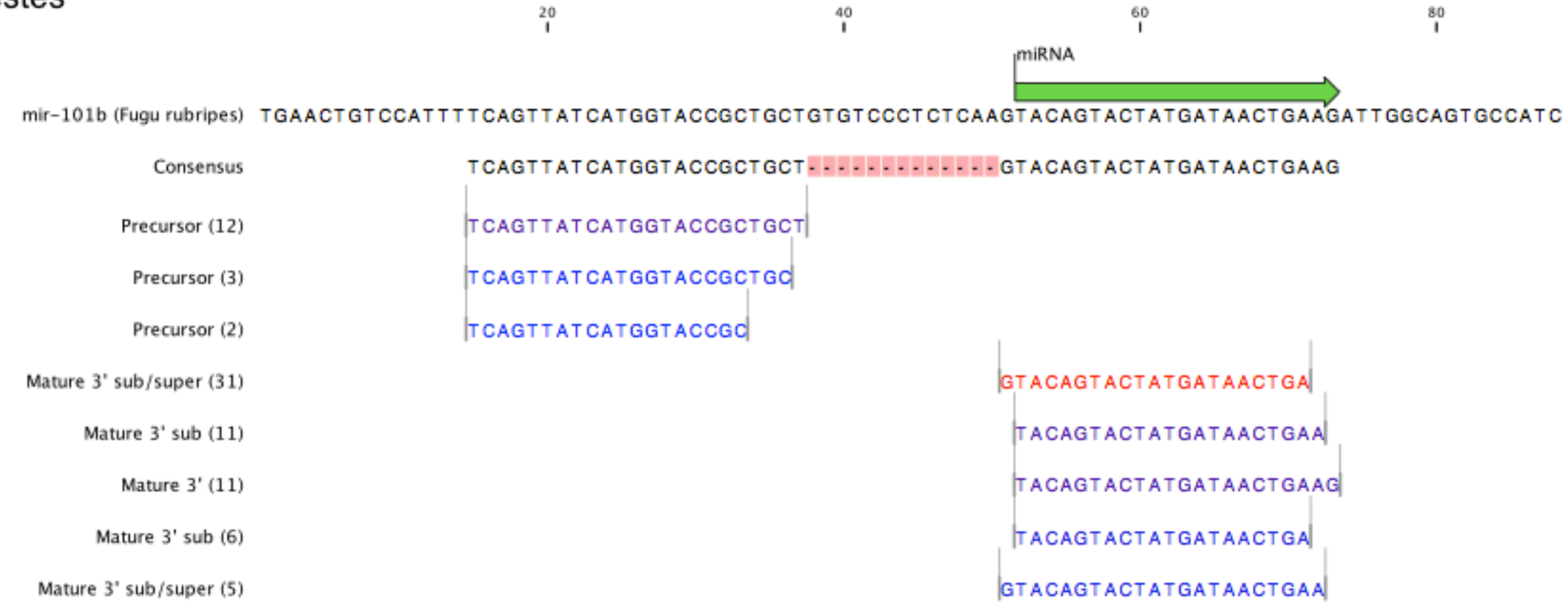
20
40
60
80
miRNA
mir-101b (Fugu rubripes) TGAACTGTCCATTTTCAGTTATCATGGTACCGCTGCTGTGTCCCTCTCAAGTACAGTACTATGATAACTGAAGATTGGCAGTGCCATC
Consensus TCAGTTATCATGGTACCGCTGCT-------------GTACAGTACTATGATAACTGAAG
Precursor (12) TCAGTTATCATGGTACCGCTGCT
Precursor (3) TCAGTTATCATGGTACCGCTGC
Precursor (2) TCAGTTATCATGGTACCGC
Mature 3' sub/super (31) GTACAGTACTATGATAACTGA
Mature 3' sub (11) TACAGTACTATGATAACTGAA
Mature 3' (11) TACAGTACTATGATAACTGAAG
Mature 3' sub (6) TACAGTACTATGATAACTGA
Mature 3' sub/super (5) GTACAGTACTATGATAACTGAA
