# Supplementary material for: Deep sequencing, profiling and detailed annotation of microRNAs in Takifugu rubripes
Source: BMC Genomics. 2015 Jun 16;16(1):457. doi: 10.1186/s12864-015-1622-1 (PMC4469249; doi:10.1186/s12864-015-1622-1)
Supplement: Additional file 13: Figure S9. — IsomiRs of fru-miR-133-3p. The diagrams represent repertoires of fru-miR-133-3p isomiRs mapped to their miRNA precursors in each tissue. [file 12864_2015_1622_MOESM13_ESM.pdf]

Additional file 13 : Figure S9

Fast muscle

mir-133 (Strongylocentrotus purpuratus)	TCCGGAAAATGTAGCGCTTTAGCTGGTAAACGGGAACCAAATCGTGAAGTAAATGCGGATTTGGTCCCCTTCAACCAGCCGTAGCA
Consensus	GATTTGGTCCCCTTCAACCAGCCGTAG
Mature 3' sub (3,858)	TTGGTCCCCTTCAACCAGCCGT
Mature 3' sub (1,686)	TTTGGTCCCCTTCAACCAGCC
Mature 3' (830)	TTTGGTCCCCTTCAACCAGCCGT
Mature 3' sub (562)	TGGTCCCCTTCAACCAGCCGT
Mature 3' sub (306)	TTGGTCCCCTTCAACCAGCC
Mature 3' sub (171)	TTTGGTCCCCTTCAACCAGCCG
Mature 3' sub (61)	TTGGTCCCCTTCAACCAGCCG
Mature 3' sub/super (36)	ATTTGGTCCCCTTCAACCAGCC
Mature 3' sub (25)	TGGTCCCCTTCAACCAGCC
Mature 3' sub (20)	TGGTCCCCTTCAACCAGCCG
Mature 3' super (14)	ATTTGGTCCCCTTCAACCAGCCGT
Mature 3' sub/super (5)	ATTTGGTCCCCTTCAACCAGCCG
Precursor (4)	GGTCCCCTTCAACCAGCCGT
Mature 3' sub/super (3)	TTGGTCCCCTTCAACCAGCCGTA
Mature 3' super (1)	GATTTGGTCCCCTTCAACCAGCCGT
Mature 3' super (1)	TTTGGTCCCCTTCAACCAGCCGTA
Mature 3' sub/super (1)	TTGGTCCCCTTCAACCAGCCGTAG
Mature 3' sub/super (1)	TGGTCCCCTTCAACCAGCCGTA
Precursor (1)	GTCCCCTTCAACCAGCCG

Slow muscle

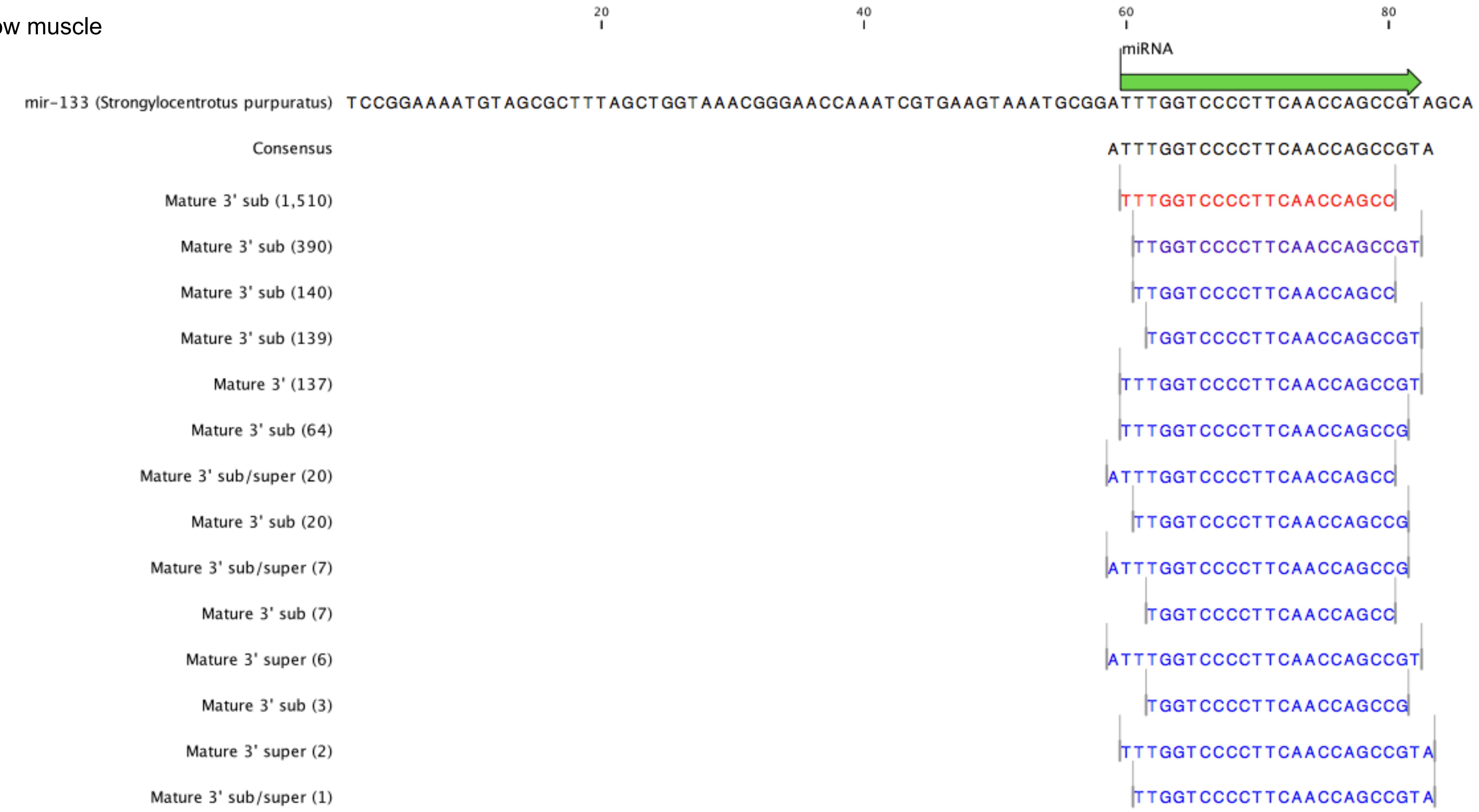
20
40
60
80
miRNA
mir-133 (Strongylocentrotus purpuratus) TCCGGAAAATGTAGCGCTTTAGCTGGTAAACGGGAACCAAATCGTGAAGTAAATGCGGATTTGGTCCCTTCAACCAGCCGTAGCA
Consensus ATTTGGTCCCTTCAACCAGCCGTA
Mature 3' sub (1,510) TTTGGTCCCTTCAACCAGCC
Mature 3' sub (390) TTGGTCCCTTCAACCAGCCGT
Mature 3' sub (140) TTGGTCCCTTCAACCAGCC
Mature 3' sub (139) TGGTCCCTTCAACCAGCCGT
Mature 3' (137) TTTGGTCCCTTCAACCAGCCGT
Mature 3' sub (64) TTTGGTCCCTTCAACCAGCCG
Mature 3' sub/super (20) ATTTGGTCCCTTCAACCAGCC
Mature 3' sub (20) TTGGTCCCTTCAACCAGCCG
Mature 3' sub/super (7) ATTTGGTCCCTTCAACCAGCCG
Mature 3' sub (7) TGGTCCCTTCAACCAGCC
Mature 3' super (6) ATTTGGTCCCTTCAACCAGCCGT
Mature 3' sub (3) TGGTCCCTTCAACCAGCCG
Mature 3' super (2) TTTGGTCCCTTCAACCAGCCGTA
Mature 3' sub/super (1) TTGGTCCCTTCAACCAGCCGTA

Heart

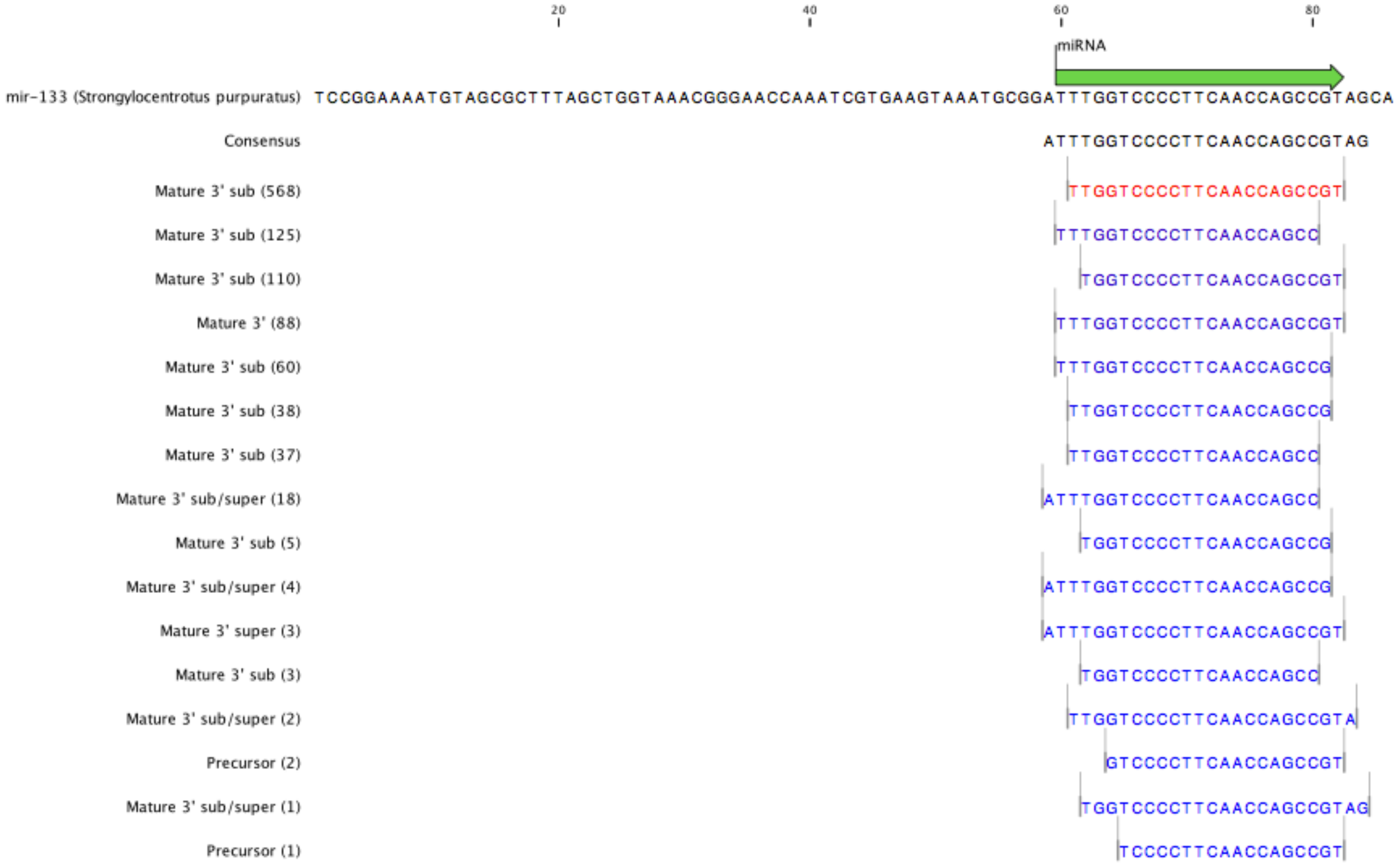
20
40
60
80
miRNA
mir-133 (Strongylocentrotus purpuratus) TCCGGAAAATGTAGCGCTTTAGCTGGTAAACGGGAACCAAATCGTGAAGTAAATGCGGATTTGGTCCCCTTCAACCAGCCGTAGCA
Consensus ATTTGGTCCCCTTCAACCAGCCGTAG
Mature 3' sub (568) TTGGTCCCCTTCAACCAGCCGT
Mature 3' sub (125) TTTGGTCCCCTTCAACCAGCC
Mature 3' sub (110) TGGTCCCCTTCAACCAGCCGT
Mature 3' (88) TTTGGTCCCCTTCAACCAGCCGT
Mature 3' sub (60) TTTGGTCCCCTTCAACCAGCCG
Mature 3' sub (38) TTGGTCCCCTTCAACCAGCCG
Mature 3' sub (37) TTGGTCCCCTTCAACCAGCC
Mature 3' sub/super (18) ATTTGGTCCCCTTCAACCAGCC
Mature 3' sub (5) TGGTCCCCTTCAACCAGCCG
Mature 3' sub/super (4) ATTTGGTCCCCTTCAACCAGCCG
Mature 3' super (3) ATTTGGTCCCCTTCAACCAGCCGT
Mature 3' sub (3) TGGTCCCCTTCAACCAGCC
Mature 3' sub/super (2) TTGGTCCCCTTCAACCAGCCGTA
Precursor (2) GTCCCCTTCAACCAGCCGT
Mature 3' sub/super (1) TGGTCCCCTTCAACCAGCCGTAG
Precursor (1) TCCCCTTCAACCAGCCGT

Eye

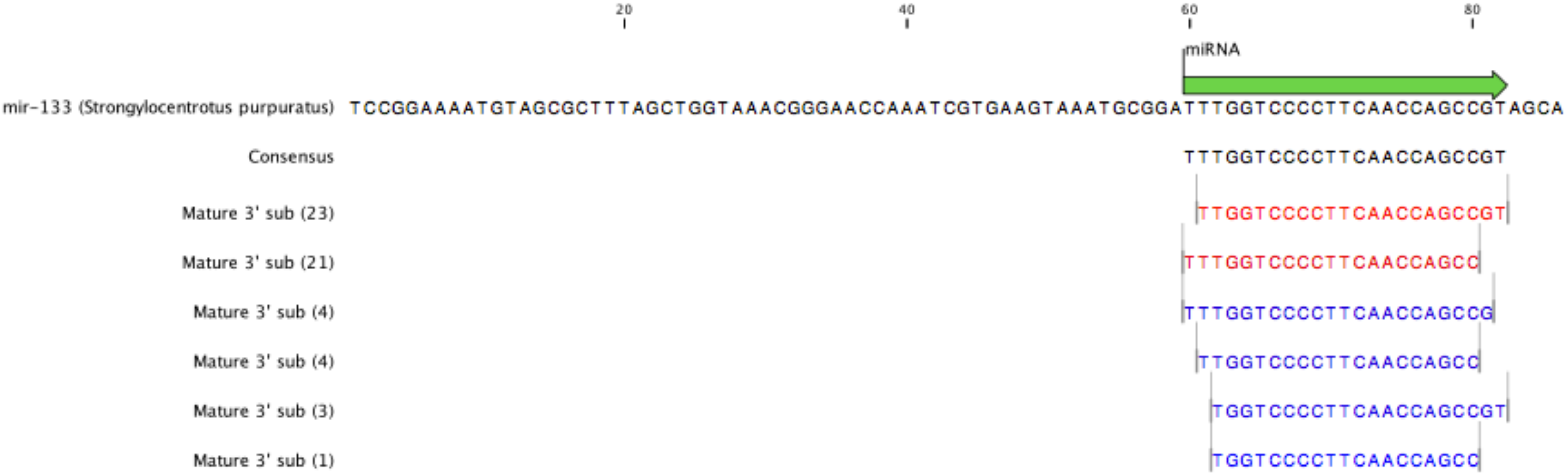

20
40
60
80
miRNA
mir-133 (Strongylocentrotus purpuratus) TCCGGAAAATGTAGCGCTTTAGCTGGTAAACGGGAACCAAATCGTGAAGTAAATGCGGATTTGGTCCCCTTCAACCAGCCGTAGCA
Consensus TTTGGTCCCCTTCAACCAGCCGT
Mature 3' sub (23) TTGGTCCCCTTCAACCAGCCGT
Mature 3' sub (21) TTTGGTCCCCTTCAACCAGCC
Mature 3' sub (4) TTTGGTCCCCTTCAACCAGCCG
Mature 3' sub (4) TTGGTCCCCTTCAACCAGCC
Mature 3' sub (3) TGGTCCCCTTCAACCAGCCGT
Mature 3' sub (1) TGGTCCCCTTCAACCAGCC

Brain

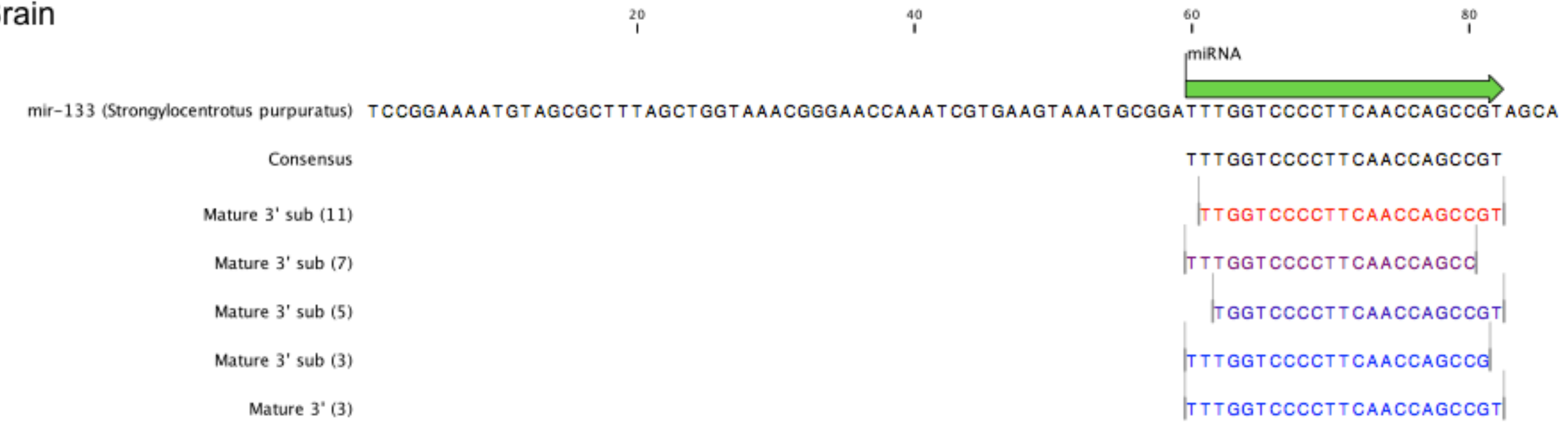

20
40
60
80
miRNA
mir-133 (Strongylocentrotus purpuratus) TCCGGAAAATGTAGCGCTTTAGCTGGTAAACGGGAACCAAATCGTGAAGTAAATGCGGATTTGGTCCCCTTCAACCAGCCGTAGCA
Consensus TTTGGTCCCCTTCAACCAGCCGT
Mature 3' sub (11) TTGGTCCCCTTCAACCAGCCGT
Mature 3' sub (7) TTTGGTCCCCTTCAACCAGCC
Mature 3' sub (5) TGGTCCCCTTCAACCAGCCGT
Mature 3' sub (3) TTTGGTCCCCTTCAACCAGCCG
Mature 3' (3) TTTGGTCCCCTTCAACCAGCCGT

Intestine

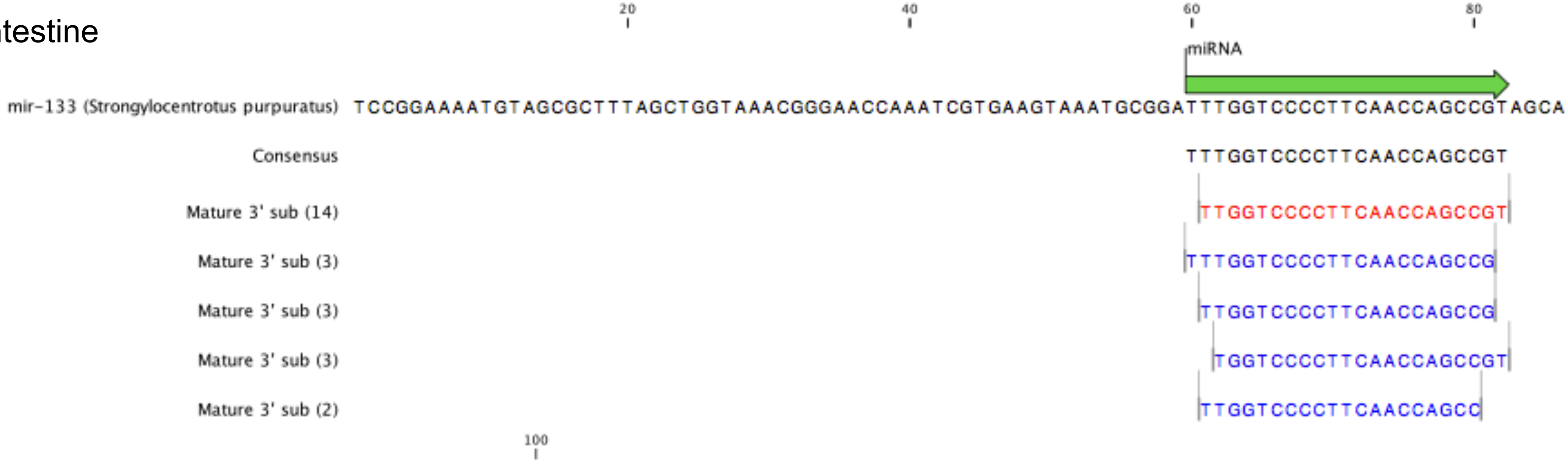

20
40
60
80
miRNA
mir-133 (Strongylocentrotus purpuratus) TCCGGAAAATGTAGCGCTTTAGCTGGTAAACGGGAACCAAATCGTGAAGTAAATGCGGATTTGGTCCCCTTCAACCAGCCGTAGCA
Consensus TTTGGTCCCCTTCAACCAGCCGT
Mature 3' sub (14) TTGGTCCCCTTCAACCAGCCGT
Mature 3' sub (3) TTTGGTCCCCTTCAACCAGCCG
Mature 3' sub (3) TTGGTCCCCTTCAACCAGCCG
Mature 3' sub (3) TGGTCCCCTTCAACCAGCCGT
Mature 3' sub (2) TTGGTCCCCTTCAACCAGCC
100

Liver

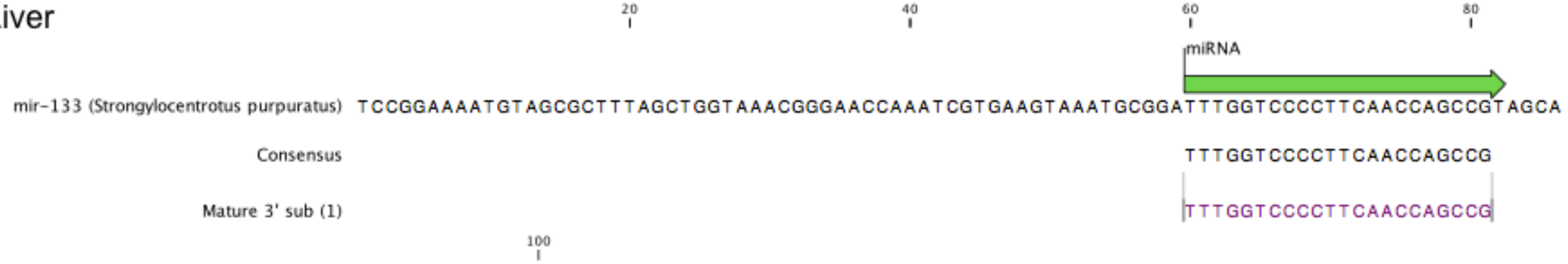
20
40
60
80
miRNA
mir-133 (Strongylocentrotus purpuratus) TCCGGAAAATGTAGCGCTTTAGCTGGTAAACGGGAACCAAATCGTGAAGTAAATGCGGATTTGGTCCCCTTCAACCAGCCGTAGCA
Consensus TTTGGTCCCCTTCAACCAGCCG
Mature 3' sub (1) TTTGGTCCCCTTCAACCAGCCG
100

Ovaries

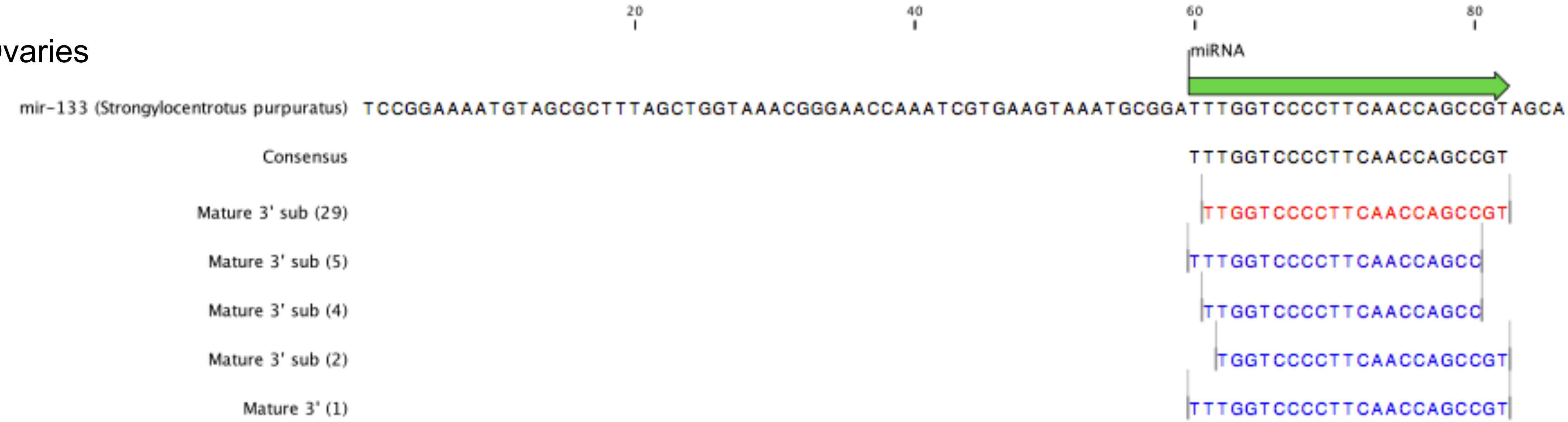
20
40
60
80
miRNA
mir-133 (Strongylocentrotus purpuratus) TCCGGAAAATGTAGCGCTTTAGCTGGTAAACGGGAACCAAATCGTGAAGTAAATGCGGATTTGGTCCCCTTCAACCAGCCGTAGCA
Consensus TTTGGTCCCCTTCAACCAGCCGT
Mature 3' sub (29) TTGGTCCCCTTCAACCAGCCGT
Mature 3' sub (5) TTTGGTCCCCTTCAACCAGCC
Mature 3' sub (4) TTGGTCCCCTTCAACCAGCC
Mature 3' sub (2) TGGTCCCCTTCAACCAGCCGT
Mature 3' (1) TTTGGTCCCCTTCAACCAGCCGT
